# Maternal Functional Hemodynamics in the Second Half of Pregnancy: A Longitudinal Study

**DOI:** 10.1371/journal.pone.0135300

**Published:** 2015-08-10

**Authors:** Åse Vårtun, Kari Flo, Tom Wilsgaard, Ganesh Acharya

**Affiliations:** 1 Women’s Health and Perinatology Research Group, Department of Clinical Medicine, Faculty of Health Sciences, UiT- The Arctic University of Norway and Department of Obstetrics and Gynecology, University Hospital of Northern Norway, Tromsø, Norway; 2 Department of Clinical Sciences, Intervention and Technology, Karolinska Institute, Stockholm, Sweden; 3 Department of Community Medicine, Faculty of Health Sciences, UiT- The Arctic University of Norway, Tromsø, Norway; University of Barcelona, SPAIN

## Abstract

**Objective:**

Cardiovascular response to passive leg raising (PLR) is useful in assessing preload reserve, but it has not been studied longitudinally during pregnancy. We aimed to investigate gestational age associated serial changes in maternal functional hemodynamics and establish longitudinal reference ranges for the second half of pregnancy.

**Materials and Methods:**

This was a prospective longitudinal study on 98 healthy pregnant women who were examined 3–5 times during 20–40 weeks of gestation (a total of 441 observations). Maternal cardiac function and systemic hemodynamics were assessed at baseline and 90 seconds after PLR using impedance cardiography (ICG). The main outcome measures were gestational age specific changes in ICG-derived variables of maternal cardiovascular function and functional hemodynamic response to PLR.

**Results:**

Hemodynamic response to PLR varied during pregnancy. PLR led to an insignificant increase in stroke volume during 20^+0^ to 31^+6^ weeks, but later in gestation the stroke volume was slightly lower at PLR compared to baseline. PLR caused no significant change in cardiac output between 20^+0^ and 23^+6^ weeks and a significant decrease after 24^+0^ weeks. A decrease in heart rate, mean arterial pressure, and cardiac contractility was observed during PLR throughout the second half of pregnancy. Systemic vascular resistance was reduced by PLR up to 32^+0^ weeks, but increased slightly thereafter.

**Conclusion:**

Healthy pregnant women appear to have limited preload reserve and reduced cardiac contractility, especially in the third trimester, which makes them vulnerable to fluid overload and cardiac failure.

## Introduction

Normal pregnancy is characterized by increased circulating blood volume [1–3], changes in maternal heart rate (HR), stroke volume (SV), cardiac output (CO) and systemic vascular resistance (SVR) [1,3–8]. The heart is slightly enlarged due to an increase in venous filling [9,10]. Hemodynamic alterations related to pregnancy are different in women who have normal pregnancy compared to those who develop pregnancy complications [11–14]. Some variables of maternal cardiovascular function may be abnormal long before the development of clinical symptoms [15–18], and could be used to stratify risk [19] and improve the management of complicated pregnancies [20].

Assessment of cardiovascular response to a physiological challenge, such as a reversible “auto-transfusion” by passive leg raising (PLR), has proved useful as a method of studying functional hemodyamics in critically ill patients [21,22]. It may be more useful to evaluate functional hemodynamics rather than just assessing the static measures of circulatory function for appropriately managing critically ill patients, including women with severe preeclampsia [20]. Transient volume load caused by PLR should lead to an increase in SV by the Frank-Starling mechanism in preload responsive individuals, and the change in SV as a result of PLR is an expression of preload reserve. However, this response is likely to be affected not only by the blood volume mobilised by PLR, but also by other factors, such as total circulating blood volume, baseline preload and cardiac contractility. We have previously shown that hemodynamic response to PLR at 20–24 weeks of gestation is similar to that observed in non-pregnant women [23]. Only a few studies with small sample size have previously investigated hormonal [24–26] and hemodynamic [27–29] responses to postural changes in healthy pregnant women. However, functional hemodynamic response to PLR has not been evaluated longitudinally in a pregnant population. Thus, we aimed to investigate gestational age associated serial changes in maternal cardiovascular response to PLR and establish reference ranges for assessing functional hemodynamics during the second half of pregnancy.

## Materials and Methods

This was a prospective longitudinal study design. The study was conducted from February 2010 to July 2013. Pregnant women attending the antenatal clinic for routine second trimester ultrasonography at 17–19 weeks of gestation were recruited to the study. They were informed about the study and invited to participate if they were >18 years, had no medical complications in the current pregnancy and the ultrasound scan showed a singleton pregnancy without any fetal or placental abnormality. Women with a previous history of pregnancy complications such as preeclampsia, gestational diabetes, intrauterine fetal growth restriction or preterm delivery were excluded.

Pregnant women were examined three to five times at approximately 4-weekly intervals, from 20 weeks of gestation until term (range 20^+1^–40^+5^ weeks). The examination took place in a quiet room with the room temperature maintained at approximately 22°C between 08:00–16:00 hours. Study participants were not fasting. Height was measured using an altimeter (Charder Electronic Co, Taichung City, Taiwan) during the first visit. Bodyweight was measured at each visit using an electronic scale (Soehnle, Leifheit AG, Nassau, Germany). Body mass index (BMI) was calculated as weight/height^2^ and the body surface area (BSA) was calculated as = 0.007184 x Height ^0.725^ x Weight ^0.425^ [30].

Parameters of maternal systemic hemodynamics were measured using impedance cardiography (ICG) (Philips Medical Systems, Androver, MA, USA). Baseline measurements were performed after 10 minutes of rest with the participant lying in a 45° supine semi-recumbent position on an electronically pivotable bed with a possibility of changing the position without any active movement by the study participant [23]. The study participants were instructed to keep quiet during ICG measurements. Four sensors were used to obtain ICG signal. Two sets of dual sensors were placed vertically on each side of the neck, and the other two dual sensors on each side of the chest in the mid-axillary line. A single operator (ÅV) examined all women using the same equipment and technique under identical conditions. Each pair of sensors consists of outer electrodes continuously sending and receiving a painless, low amplitude (1mA) electric signal through the thorax. The two inner electrodes detect and measure the change in impedance, which is directly related to the blood volume change in the thoracic aorta throughout the cardiac cycle.

A sphygmomanometer cuff placed on the left arm for blood pressure measurement was connected to the ICG machine. Woman’s height, present weight, and age were entered into the machine. Central venous pressure (CVP) and pulmonary artery occlusion pressure (PAOP) were pre-set at 4 and 8 mmHg, respectively. The following variables were directly measured and displayed on the ICG screen: heart rate (HR), stroke volume (SV), systolic blood pressure (SBP), diastolic blood pressure (DBP), pre-ejection period (PEP), left-ventricular ejection time (LVET), thoracic fluid content (TFC), velocity index (VI) and accelerated cardiac index (ACI). The following variables were calculated as follows: CO = SV x HR, cardiac index (CI) = CO/BSA, MAP = DBP + 1/3 (SBP-DBP), systemic vascular resistance (SVR) = ((MAP-CVP)/CO) x 80, systemic vascular resistance index (SVRI) = SVR/BSA, systolic time ratio (STR) = PEP/LVET, left cardiac work index (LCWI) = (MAP-PAOP) x CO/BSA x 0.0144. A repeatability study was performed in 20 women, each at a different gestational age, which showed a coefficient variation of 3.16% (95% CI, 1.23–5.08) for SV, 4.67% (95% CI, 1.96–7.38) for HR, 3.27% (95% CI, 2.16–4.37) for CO, and 2.65% (95% CI, 1.73–3.58) for SVR.

After obtaining the baseline measurements, the participant’s upper body was lowered to a supine position, and PLR was performed raising both legs to 45° by tilting the lower portion of the bed. The ICG measurements were recorded approximately 90 seconds after PLR. The cardiovascular response to PLR was calculated as percent change (∆ %) of the hemodynamic variables from baseline to PLR, i.e. (measurement during PLR–baseline measurement) / baseline measurement x 100%. The SV ∆ % was considered as an estimate for preload reserve.

### Statistical analysis

Data analysis was performed using IBM SPSS statistics (SPSS software, version 21.0, Inc., Chicago, IL, USA) and SAS version 9.3 (SAS Institute INC., Cary, NC, USA). The number of study participants required to establish normal reference values was estimated to be approximately 100 based on the assumption that 20 observations per gestational week (i.e. a total of 400 observations between 20–40 weeks) would be sufficient to calculate reference intervals with adequate precision [31]. Categorical variables are presented as n (%) and continuous variables as mean (±SD) or median (range) as appropriate. Comparison between baseline values and values obtained during PLR was performed using paired t-test. Statistical significance was set to p<0.05. Assumption of normality was checked for each variable and logarithmic or power transformations were performed to achieve normal distribution when required. Fractional polynomials were used to obtain best fitting curves in relation to gestational age accommodating for nonlinear associations. We applied multilevel regression modelling using proc mixed in SAS to investigate gestational age associated changes in functional hemodynamics and estimate the reference percentiles [32,33] accounting for possible dependency between repeated measures. We fitted individual observations as a linear function for the fractional polynomial term of time, i.e. the gestational age. We included a random intercept term for each individual and a random slope was used for the fractional polynomial term of the gestational age.

### Ethics statement

All study participants gave informed written consent. The study was approved by the Regional Committee for Medical and Health Research Ethics–North Norway (Ref.nr. 2010/575-2. Date of approval: 10.02.2010).

## Results

A total of 102 pregnant women consented to this longitudinal study. Of these, 25 women had also participated in a cross-sectional study investigating differences in functional hemodynamics between pregnant and non-pregnant women [23]. Four women were excluded from analysis because their medical records revealed that they had hypertensive disorders in previous pregnancies. Data from 98 study participants (441 observations) were used for the final statistical analysis. The number of participants examined during pregnancy was 98 (20–24), 87 (24–28), 90 (28–32), 80 (32–36) and 82 women (>36) weeks of gestation. The baseline characteristics of the study population and data on pregnancy outcome are given in Table 1. 99% of the women were non-smokers. The mean weight gain was 9.1 Kg (12.7%), from booking to the last examination.

**Table 1:** Baseline characteristics of the study population (N = 98).

Parameter	Result
**Maternal**	
Age (years)	29 (range 19–39)
Body mass index at first examination (Kg/m^2^) (20–24 weeks)	25.67 (± 3.62)
Body surface area (m^2^)	1.79 (± 0.14)
Nullipara (n%)	43 (44)
Mean arterial pressure at baseline, first examination (mmHg) (20–24 weeks)	79.92 (± 6.97)
**Fetal**	
Gestational age at birth (weeks)	40 (32–42)
Birth weight (g)	3520 (± 480)
Placental weight (g)*	607 (± 119)
5-minute Apgar score**	10 (2–10)
Umbilical artery pH#	7.24 (± 0.09)
Umbilical artery base excess (mmol/L)#	-3.90 (± 3.77)

Data presented as n (%), median (range) or mean (± SD) as appropriate.

# n = 50

*4 missing values

**1 missing value.

Eight women were delivered by CS (three due to prolonged labour, four due to fetal distress and one due to breech presentation and a history of previous CS).Three women delivered preterm (<37 weeks): two of them had spontaneous vaginal delivery at 33^+3^ and 36^+5^ weeks, respectively and one had an emergency caesarean section (CS) due to fetal distress assosciated with placental abruption at 32^+4^weeks. In another three women labour was induced due to postdate.

Two neonates had Apgar score <7 at five minutes. A total of eight neonates were transferred to the neonatal care unit for observation or treatment. One of them, delivered preterm due to placental abruption, had intraventricular haemorrhage leading to neurodevelopmental delay and hydrocephalus requiring ventriculo-peritoneal shunt. The remaining infants were discharged home in good condition.

**Fig 1:**
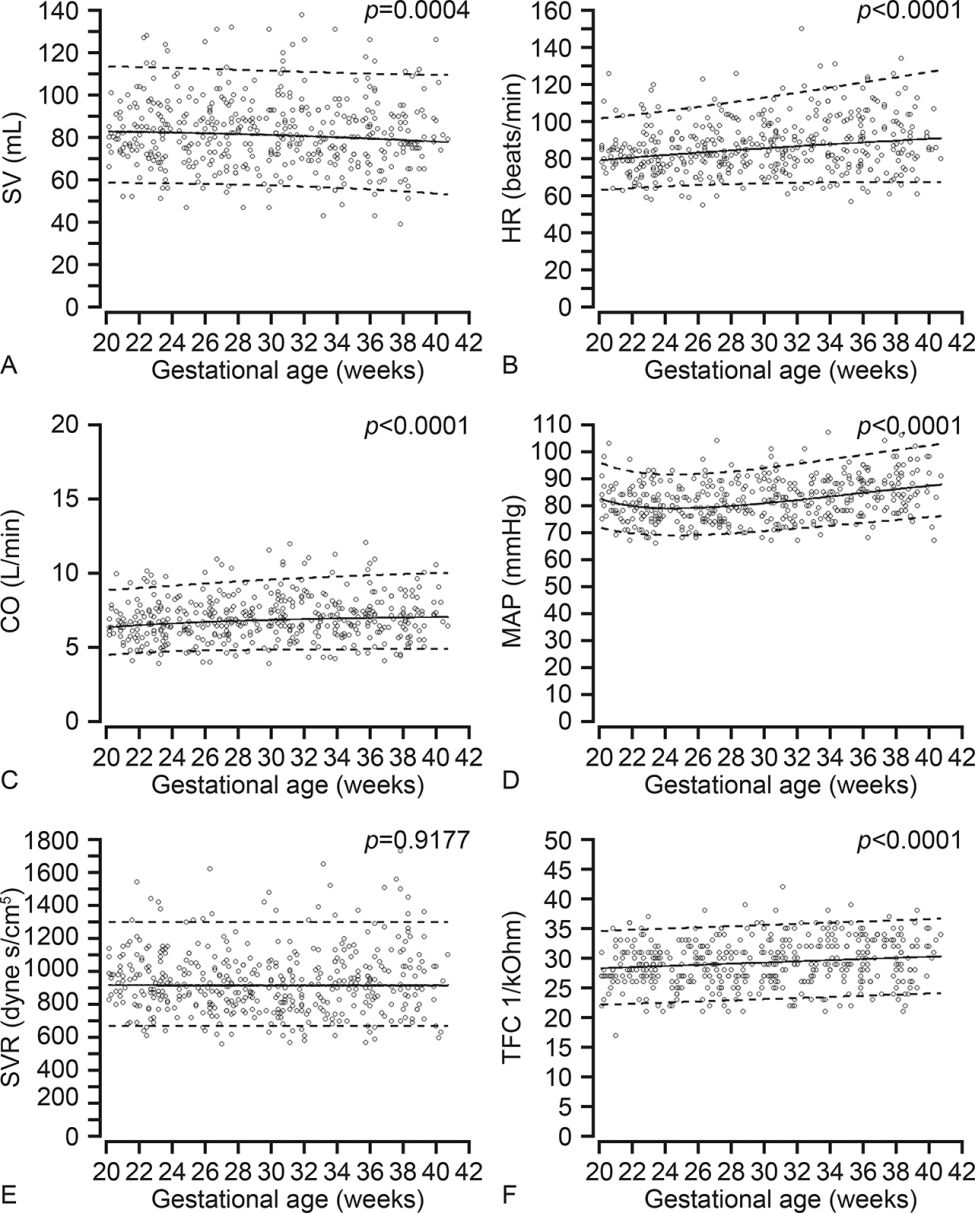
Longitudinal changes in maternal systemic blood flow, blood pressure, vascular resistance and thoracic fluid content during the second half of pregnancy. A. SV, stroke volume; B. HR, heart rate; C. CO, cardiac output; D. MAP, mean arterial pressure; E. SVR, systemic vascular resistance and F. TFC, thoracic fluid content.

**Fig 2:**
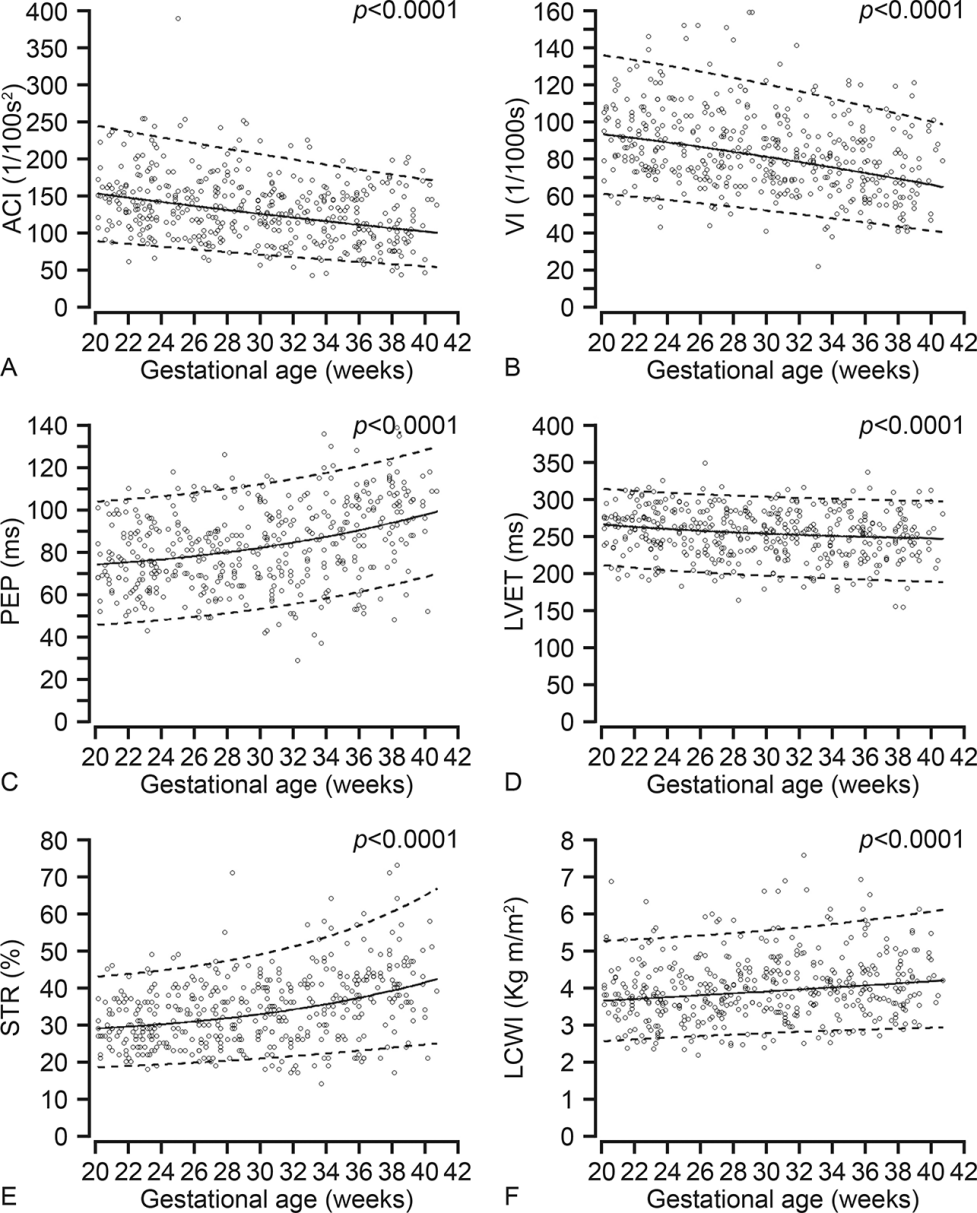
Longitudinal changes in parameters describing maternal cardiac contractility and work measured using impedance cardiography. A. ACI, acceleration index; B. VI, velocity index; C. PEP, pre-ejection period; D. LVET, left ventricular ejection time; E. STR, systolic time ratio and F. LCWI, left ventricular work index.

In S1 Table the individual measurements of maternal systemic hemodynamic variables including baseline characteristics and pregnancy outcome are presented. The variables describing maternal systemic hemodynamics and cardiac function measured by ICG at baseline during the second half of pregnancy are presented in Figs 1 and 2 as the mean values with their respective 5^th^ and 95^th^ percentiles. Gestational age specific reference values for all these variables are presented in S2–S13 Tables. The mean differences (delta-values) between values measured at baseline and PLR are presented in Table 2.

**Table 2:** Mean differences between hemodynamic variables measured by impedance cardiography at baseline and 90 seconds after passive leg raising during the second half of pregnancy.

Variables	GA (weeks)	p-value	GA (weeks)	p-value	GA (weeks)	p-value	GA (weeks)	p-value	GA (weeks)	p-value
	20–24		24–28		28–32		32–36		> 36	
SV	1.14 (-0.31;2.59)	0.121	0.72 (-0.94;2.39)	0.390	-1.01 (-2.83;0.81)	0.272	-1.80 (-4.20;0.60)	0.139	-1.00 (-3.44;1.44)	0.418
CO	-0.04 (-0.16;0.08)	0.496	-0.16 (-0.29;-0.03)	0.017	-0.31 (-0.46;-0.16)	<0.001	-0.40 (-0.57;-0.23)	<0.001	-0.37 (-0.57;-0.17)	<0.001
CI	-0.02 (-0.09;0.04)	0.487	-0.08 (-0.16;-0.01)	0.022	-0.16 (-0.24;-0.08)	<0.001	-0.22 (-0.31;-0.13)	<0.001	-0.20 (-0.30;-0.09)	<0.001
HR	-2.54 (-3.52;-1.56)	<0.001	-2.52 (-3.79;-1.24)	<0.001	-3.62 (-4.97;-2.28)	<0.001	-2.81 (-4.87;-0.75)	0.008	-5.05 (-7.03;-3.07)	<0.001
BPS	-3.92 (-4.92;-2.91)	<0.001	-2.22 (-3.23;-1.21)	<0.001	-2.69 (-3.92;-1.46)	<0.001	-2.35 (-3.50;-1.20)	<0.001	-1.61 (-2.77;-0.45)	0.007
BPD	-4.93 (-5.73;-4.13)	<0.001	-3.63 (-4.52;-2.73)	<0.001	-3.73 (-4.51;-2.95)	<0.001	-4.46 (-5.23;-3.70)	<0.001	-4-07 (-4.96;-3.19)	<0.001
MAP	-4.59 (-5.34;-3.83)	<0.001	-3.20 (-3.93;-2.47)	<0.001	-3.42 (-4.16;-2.68)	<0.001	-3.78 (-4.53;-3.02)	<0.001	-3.27 (-4.06;-2.48)	<0.001
SVR	-50.51 (-70.48;-30.54)	<0.001	-23.72 (-45.90;-1.54)	0.036	-3.44 (-23.01;16.13)	0.727	4.50 (-19.86;28.86)	0.714	5.37 (-19.10;29.83)	0.664
SVRI	-90.61 (-125.83;-55.40)	<0.001	-40.70 (-80.22;-1.18)	0.044	-5.78 (-40.98;29.43)	0.745	7.88 (-36.98;52.73)	0.728	11.59 (-35.06;58.23)	0.622
TFC	1.04 (0.73;1.35)	<0.001	1.59 (0.54;2.63)	0.003	0.78 (0.37;1.19)	<0.001	1.00 (0.56;1.44)	<0.001	0.78 (0.46;1.10)	<0.001
ACI	-6.08 (-11.12;-1.05)	0.018	-5.61(-14.34;3.12)	0.205	-9.79 (-14.81;-4.77)	<0.001	-13.39 (-18.62;-8.15)	<0.001	-10.71 (-16.81;-4.61)	0.001
LCWI	-0.28 (-0.36;-0.20)	<0.001	-0.25 (-0.34;-0.17)	<0.001	-0.37 (-0.48;-0.27)	<0.001	-0.43 (-0.55;-0.32)	<0.001	-0.37 (-0.50;-0.24)	<0.001
PEP	-7.80 (-9.72;-5.87)	<0.001	-4.66 (-7.20;-2.11)	<0.001	-0.34 (-3.23;2.54)	0.813	1.75 (-1.88;5.38)	0.340	-3.24 (-7.43;0.94)	0.127
LVET	10.80 (3.41;18.18)	0.005	13.75 (7.53;19.97)	<0.001	5.39 (-1.80;12.57)	0.140	6.68 (-0.82;14.17)	0.080	5.34 (-1.99;12.68)	0.151
VI	-6.67 (-8.97;-4.36)	<0.001	-6.70 (-10.59;-2.81)	0.001	-5.60 (-8.16;-3.04)	<0.001	-8.15 (-11.33;-4.97)	<0.001	-5.62 (-8.37;-2.87)	<0.001
STR	-4.06 (-5.23;-2.89)	<0.001	-3.34 (-4.64;-2.05)	<0.001	-0.63 (-2.44;1.17)	0.488	-0.08 (-2.31;2.16)	0.947	-2.41 (-4.86;0.03)	0.053

Data are presented as mean values for the difference (delta-values) between the measurements recorded at PLR and baseline (95% confidence intervals). p <0.05 was considered as significant. GA, gestational age; SV, stroke volume (ml); CO, cardiac output (L/min); cardiac index (L/min/m^2^); HR, heart rate (beats/min); BPS, systolic blood pressure (mm Hg); BPD, diastolic blood pressure (mm Hg); MAP, mean arterial pressure (mm Hg); SVR, systemic vascular resistance (dyne s/cm^5^); SVRI, systemic vascular resistance index (dyne s m^2^/cm^5^); TFC, thoracic fluid content (1/kOhm); ACI, acceleration index (1/100 s^2^); LCWI, left ventricular work index (Kg m/m^2^); PEP, pre-ejection period (ms); LVET, left ventricular ejection time (ms); VI, velocity index (1/1000s) and STR, systolic time ratio (%).

The mean values of variables describing cardiac function and systemic hemodynamics measured at baseline and PLR during different gestational ages are presented in S14 Table. The mean quantitative effect of modified preload on cardiac function induced by PLR varied by gestation, and varied among individual women at different gestations.

At baseline, the SV increased from 83.0±15.83 ml during 20–24 weeks to a maximum of 84.6±16.74 ml at 28–32 weeks, and then decreased to 81.7±16.62 ml during 36–40 weeks. The CO was 6.58±1.34 L/min at 20–24 weeks, increased to 7.14 ±1.46 L/min (8.5%) at 28–32 weeks, and then decreased to 7.03±1.57 L/min at 32–36 weeks and 7.11 L/min at 36–40 weeks. The HR and TFC increased steadily from 82/min to 90/min and from 28.63/kOhm to 30.16/kOhm, respectively during 20–40 weeks. The blood pressure was lowest at 24–28 weeks, and then there was a steady increase towards term. The SVR was lowest (899 dyne s/cm^5^) at 28–32 weeks, thereafter it increased slightly to 971 dyne s/cm^5^ at term. The LCWI was 3.81 Kg m/m^2^ at 20–24 weeks and increased by 10.5% to 4.21 Kg m/m^2^ close to term. The PEP increased from 76ms to 95ms (24.3%), and the LVET decreased from 260ms to 247ms (4.9%) from 20 weeks to term, and as a result the STR increased by 32.8% during the same period. The ACI and VI decreased by 25.1% and 21.3%, respectively from 20 weeks to term.

**Fig 3:**
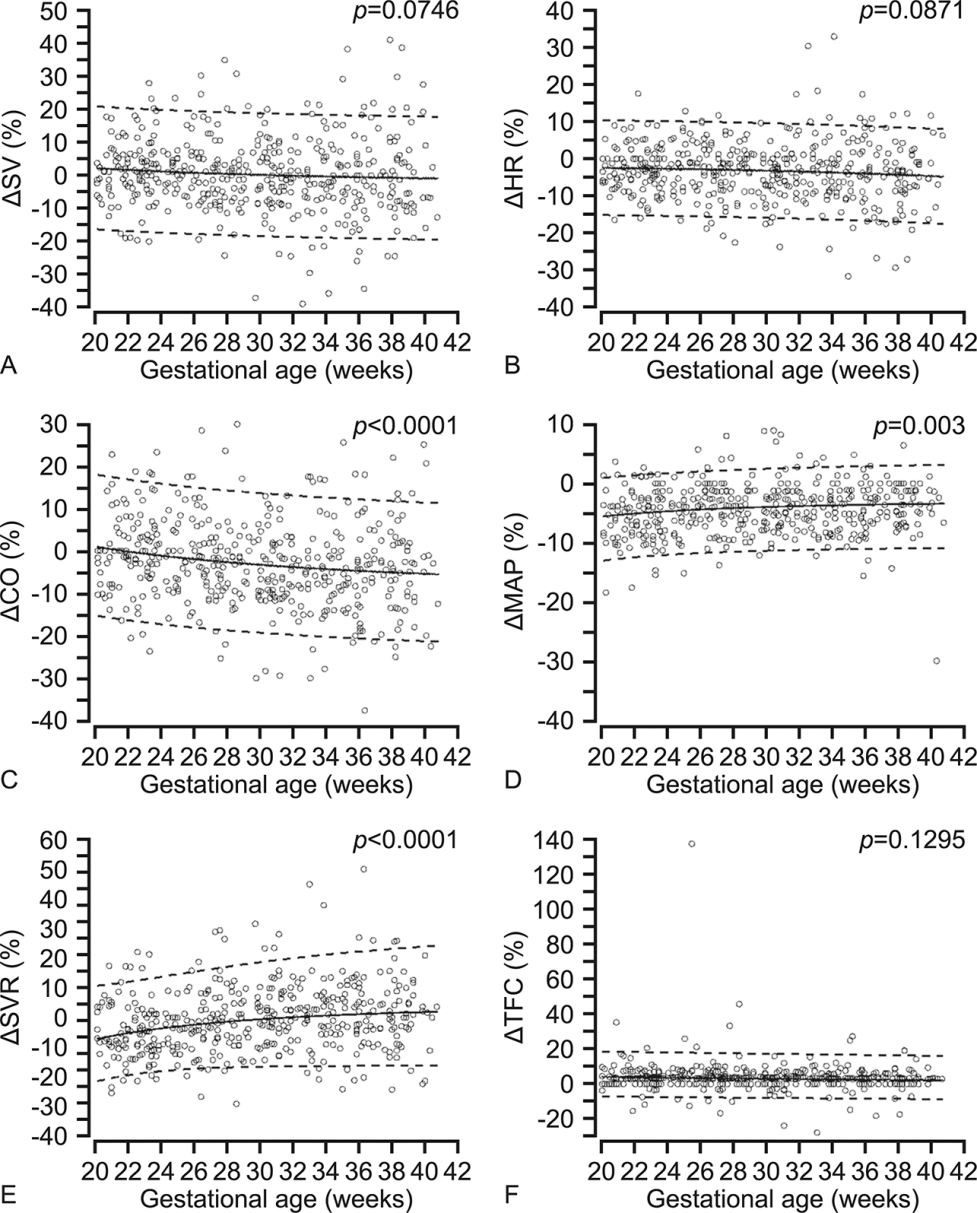
Gestational age specific longitudinal reference ranges of the percent change (∆%) in maternal systemic blood flow, blood pressure, vascular resistance and thoracic fluid content measured from baseline to passive leg raising. The solid line represents the mean and the interrupted lines represent the 5^th^ and 95^th^ percentiles. A. ∆SV, stroke volume; B. ∆HR, heart rate; C. ∆CO, cardiac output; D. ∆MAP, mean arterial pressure; E. ∆SVR, systemic vascular resistance and F. ∆TFC, thoracic fluid content.

**Fig 4:**
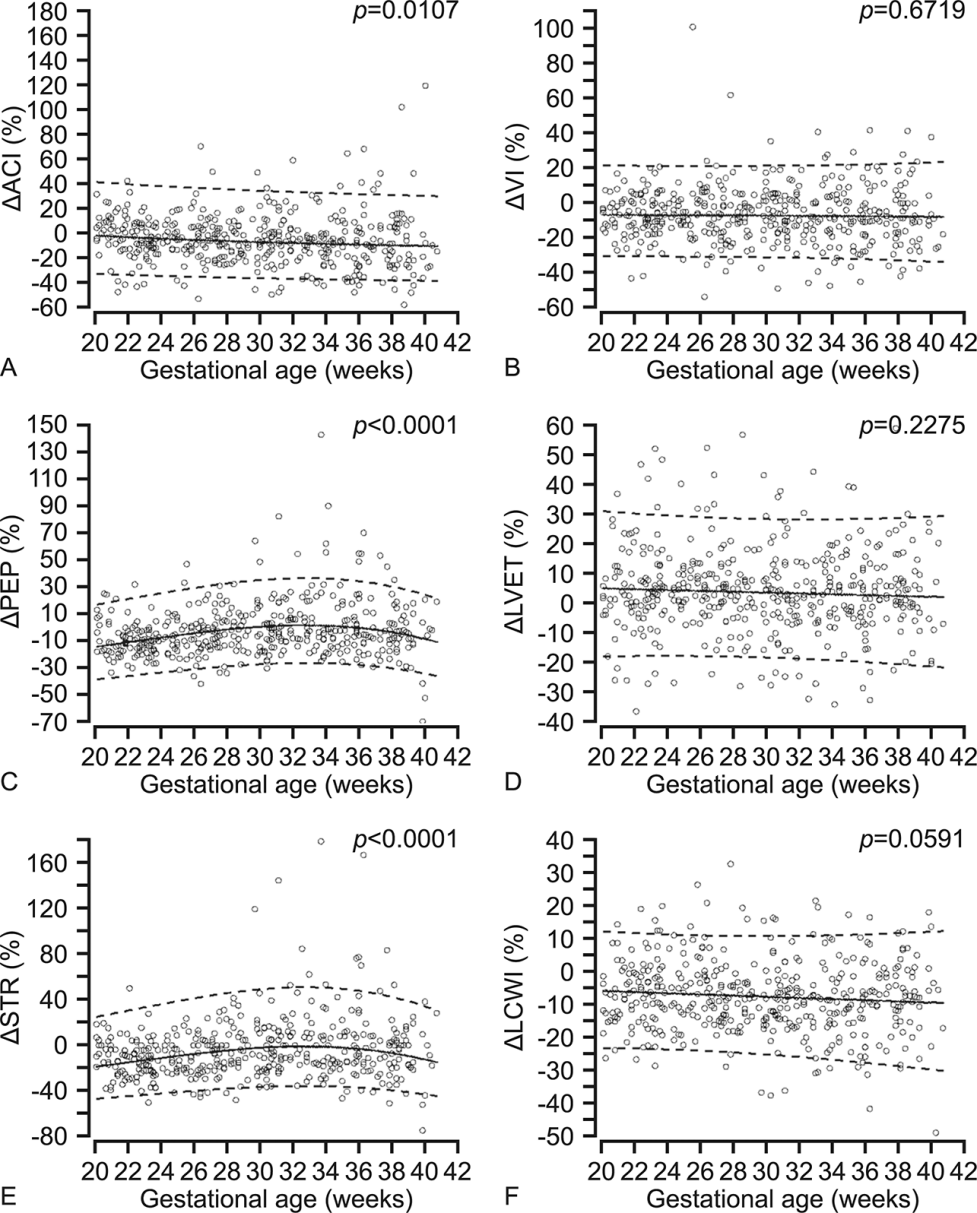
Gestational age specific longitudinal reference ranges of the percent change (∆%) in maternal cardiac contractility and work from baseline to passive leg raising. The solid line represents the mean and the interrupted lines represent the 5^th^ and 95^th^ percentiles. ∆ACI, acceleration index; B. ∆VI, velocity index; C. ∆PEP, pre-ejection period; D. ∆LVET, left ventricular ejection time; E. ∆STR, systolic time ratio and F. ∆LCWI, left ventricular work index.

Longitudinal changes in cardiovascular response to PLR are presented as gestational age specific reference ranges (mean % change of the measured variables from baseline to PLR with their respective 5^th^ and 95^th^ percentiles) in Figs 3 and 4. There was no significant association between the gestational age and % change in SV (p = 0.075) or HR (p = 0.087) from baseline to PLR. PLR led to a small insignificant increase in SV during 20^+0^–31^+6^ weeks, but later in gestation the SV was slightly lower at PLR compared to baseline. The HR was 2.5–4.7% lower during PLR compared to baseline. The % change in CO was associated with gestational age (p<0.0001). It was similar at baseline and PLR at 20^+0^–23^+6^ weeks but was significantly decreased by PLR from 1.2% at 24 weeks to 5.4% at term. PLR induced 4% increase in TFC at 20–26 weeks, 3% at 27–37 weeks and 2% at 38–40 weeks compared to baseline, but the change was not associated with the gestational age (p = 0.130). The blood pressure was reduced by PLR, and the % change in BPS (p = 0.002), BPD (p = 0.033), and MAP (p = 0.003) was significantly associated with gestational age with lower differences between values measured at baseline and PLR with increasing gestation. The change in SVR was significantly (p<0.0001) associated with gestational age. SVR was decreased by PLR until 32 weeks, but increased slightly from 32 weeks onwards.

The % changes of ACI (p = 0.011), PEP (p<0.0001) and STR (p<0.0001) from baseline to PLR were significantly associated with the gestational age, but the % change in VI (p = 0.672) and LVET (p = 0.228) were not. During PLR, ACI decreased from 2% at 20 weeks to 10% at term compared to baseline values. The LVET was slightly increased by PLR (5.1% at 20 weeks compared to 2.3% at 40 weeks). The % reduction in VI from baseline to PLR was stable (7–8%) throughout the second half of pregnancy, but the reduction in STR varied (1–19%). The PEP was decreased significantly by PLR until 28 weeks, then increased very slightly until 36 weeks and decreased again towards term. The LCWI decreased during PLR by 5.9% at 20 weeks compared to 9.4% at term.

## Discussion

We investigated hemodynamic response to PLR in healthy pregnant women from mid-gestation to term using noninvasive ICG. Cardiovascular response to PLR varied during 20–40 weeks of gestation. The functional hemodynamic response to PLR was positive during 20^+0^–31^+6^ weeks, but negative during 32^+0^–40^+0^ weeks, but the changes in stroke volume caused by PLR were of small magnitude (-0.8 to 2.3%) and not significant. The HR was reduced by PLR leading to a progressive reduction in CO during PLR compared to baseline in the third trimester of pregnancy. PLR caused a reduction in blood pressure, cardiac contractility and cardiac work compared to baseline throughout the second half of pregnancy.

There are no published studies of functional hemodynamics in normal pregnancy investigating cardiovascular response to PLR longitudinally to compare our results with. However, the baseline measurements of cardiovascular function obtained during the second half of pregnancy in this study were similar to those reported previously by us [7] and others [8] using similar equipment and methodology. A study performed on pregnant women at term [34] reported no significant difference between CO measured at baseline and during PLR in the semi-recumbent or left lateral decubitus positions, but a decrease in CO during PLR in the right-lateral decubitus position. This was a cross-sectional study that used a different method (pulse contour analysis) and device (ccNexfin; Edwards Lifesciences, Irvine, CA) to estimate the CO. However, their results, as ours, indicate that pregnant women are not able to increase CO during PLR in late gestation. Some investigators have previously examined cardiovascular response to orthostatic stress in pregnant women using a variety of methods and postural changes [25,27,29,35,36]. Higher values of SV and CO are generally reported in left lateral compared to supine [27,37] or standing [36] position. However, the results from those studies cannot be directly compared with that of our study as they are likely to be related to autonomic response rather than change in preload.

Gestational age related reduction in ACI and VI, prolongation of PEP and shortening of LVET suggested a decrease in baseline cardiac contractility with advancing gestation. This is in line with Estensen et al. [38] who found reduced the left ventricular contractility during pregnancy based on reduced left ventricle ejection fraction. Geva et al. [39] also reported a transient decrease in contractility in the second trimester using serial echocardiographic measurements during and after pregnancy. In our study cardiac contractility and cardiac work was not improved by increasing preload by PLR in pregnant women as evidenced by the reduction in ACI, VI, STR and LCWI.

We have previously compared the effect of PLR between non-pregnant and pregnant women at 22–24weeks of gestation in a cross-sectional study and found that they respond similarly to transient volume load [23]. However, the results of our current longitudinal study indicate that under normal physiologic conditions the cardiovascular response to PLR is different in the third trimester compared to earlier in pregnancy. We found that in pregnant women, the heart is not able respond to increased preload by increasing stroke volume after 32 weeks. Reduced contractility could be one of the mechanisms leading to inability of the heart to increase its SV in response to auto-fluid challenge. We also found that the PLR increased total SVR after 32 weeks despite decreasing the blood pressure, indicating reduced ability of heart to increase CO in late pregnancy.

Our study participants were all healthy women with low risk pregnancies, and in a normal state of hydration. It has been shown that the increase in CO in response to PLR is larger after withdrawal of blood in healthy subjects [28], and the response is shown to be of higher magnitude in preload responsive volume depleted preeclamptic women [20]. In our previous study, we found that healthy non-pregnant women have a relatively low preload reserve [23] compared to that reported in volume depleted patients [40,41]. Furthermore, we found that the TFC increased only by 2–4% during PLR in pregnant women, suggesting that the amount of fluid transferred to the central circulation is small.

Another explanation for the negative response to PLR could be related to inability to increase preload by PLR in the third trimester due to uterine compression of the inferior vena cava (IVC) and compromised venous return. However, IVC compression does not occur very often even in late pregnancy. A study comparing the diameter of the IVC in the supine position and the left lateral tilt at term found that 25% of women had the largest IVC diameter in the supine position [42]. Even in case of IVC occlusion, the venous return to the heart is directed via collaterals (ascending lumbar veins) maintaining its preload [43]. Furthermore, we have previously found a good agreement between ICG measurements performed in left lateral and supine semi-recumbent positions in 20 women in late gestation [7].

Our study provides longitudinal reference ranges for the assessment of functional hemodynamics in pregnant women. We used noninvasive ICG, which is well validated for the hemodynamic assessment of pregnant women [44–49]. Compared to echocardiography, it is operator-independent, quick and user friendly, which makes it ideally, suited for bedside functional hemodynamic assessment. Although the accuracy of CO measurement in pregnant women using ICG has been questioned in the past, new generation of bioimpedance devices are reported to be accurate, and provide reliable measurement of maternal hemodynamics [50]. All participants were examined by a single operator using the same equipment under identical conditions. There were no dropouts after recruitment, and the follow up was complete. However, our study has some limitations. As we did not examine the participants before pregnancy, during the first half of gestation or postpartum, the reference ranges are established only for the second half of pregnancy. Our study population consisted of white European women, and the results might not be applicable to other ethnic groups. There could be heterogeneity in hemodynamics status related to age [51]. It has been shown previously that maternal cardiac function may be affected by parity [52]. Parous women appear to have higher CO and lower SVR compared to nulliparous women. Our study included healthy pregnant women aged 19–39 years, but did not evaluate separately the results from multiparous versus nulliparous women.

Functional hemodynamic evaluation using PLR has been shown to be useful in predicting preload responsiveness and guiding fluid therapy. However, it would be important to take into account the individual as well as gestational age associated physiological variations in quantitative effect of modified preload throughout the pregnancy. A recent study in preeclamptic women with oliguria showed that it is possible to predict fluid responsiveness using PLR [20]. It is known that pregnant women with severe preeclampsia and oliguria are at risk of developing pulmonary oedema and cardiac failure [53,54]. PLR could possibly be used to stratify women into those who would benefit from volume expansion or fluid restriction, and monitor as well as guide fluid and antihypertensive therapy.

## Conclusion

In summary, our study provides longitudinal reference ranges for functional hemodynamic assessment of pregnant women during the second half of pregnancy. Healthy pregnant women appear to have limited preload reserve and reduced cardiac contractility, especially in the third trimester, which makes them vulnerable to fluid overload and cardiac failure.

## Supporting Information

S1 Table Individual measurements of maternal systemic hemodynamic variables during second half of pregnancy with baseline characteristics and pregnancy outcome. (_A)—measured at baseline position and (_B)—measured 90 seconds after passive leg raising.
(PDF)

S2 Table Longitudinal reference ranges for the maternal stroke volume (ml) during second half of pregnancy. (DOCX)

S3 Table Longitudinal reference ranges for the maternal heart rate (beats/min) during second half of pregnancy. (DOCX)

S4 Table Longitudinal reference ranges for the maternal cardiac output (L/min) during second half of pregnancy. (DOCX)

S5 Table Longitudinal reference ranges for the maternal mean arterial pressure (mmHg) during second half of pregnancy. (DOCX)

S6 Table Longitudinal reference ranges for the maternal systemic vascular resistance (dyne s/cm^5^) during second half of pregnancy. (DOCX)

S7 Table Longitudinal reference ranges for the maternal thoracic fluid content (1/kOhm) during second half of pregnancy. (DOCX)

S8 Table Longitudinal reference ranges for the maternal acceleration index (1/100 s^2^) during second half of pregnancy. (DOCX)

S9 Table Longitudinal reference ranges for the maternal velocity index (1/1000s) during second half of pregnancy. (DOCX)

S10 Table Longitudinal reference ranges for the maternal pre-ejcetion period (ms) during second half of pregnancy. (DOCX)

S11 Table Longitudinal reference ranges for the maternal left ventricular ejection time (ms) during second half of pregnancy. (DOCX)

S12 Table Longitudinal reference ranges for the maternal systolic time ratio (%) during second half of pregnancy. (DOCX)

S13 Table Longitudinal reference ranges for the maternal left ventricular work index (Kg m/m^2^) during second half of pregnancy. (DOCX)

S14 Table Hemodynamic variables measured by impedance cardiography at baseline and 90 seconds after passive leg raising during the second half of pregnancy. Data are presented as mean values for baseline and PLR (± standard deviation). GA, gestational age; SV, stroke volume (ml); HR, heart rate (beats/min); CO, cardiac output (L/min); MAP, mean arterial pressure (mmHg); SVR, systemic vascular resistance (dyne s/cm^5^); TFC, thoracic fluid content (1/kOhm); ACI, acceleration index (1/100 s^2^); VI, velocity index (1/1000s); PEP, pre-ejection period (ms); LVET, left ventricular ejection time (ms); STR, systolic time ratio (%) and LCWI, left ventricular work index (Kg m/m^2^).
(DOCX)
