# Supplementary material for: Maternal Functional Hemodynamics in the Second Half of Pregnancy: A Longitudinal Study
Source: PLoS One. 2015 Aug 10;10(8):e0135300. doi: 10.1371/journal.pone.0135300 (PMC4530890; doi:10.1371/journal.pone.0135300)
Supplement: S1 Table — (_A)—measured at baseline position and (_B)—measured 90 seconds after passive leg raising. (PDF) [file pone.0135300.s001.pdf]

No	Age	Weeks	Days	BMI	BSA	CO_A
1	29,00	20,00	2,00	23,18	1,87	7,60
		24,00	2,00	31,99	1,90	7,40
		30,00	4,00	32,74	1,92	8,10
		35,00	1,00	33,87	1,95	8,20
		39,00	1,00	35,38	1,98	7,40
2	27,00	20,00	2,00	25,56	1,63	7,80
		23,00	2,00	26,00	1,64	7,60
		28,00	2,00	27,20	1,67	6,60
		32,00	3,00	28,00	1,70	8,40
		36,00	,00	28,40	1,71	7,70
3	19,00	20,00	1,00	29,02	1,86	6,90
		27,00	4,00	30,12	1,89	6,90
		31,00	1,00	30,85	1,91	6,10
		35,00	1,00	30,85	1,91	6,20
		38,00	1,00	32,00	1,94	6,20
4	21,00	20,00	1,00	24,74	1,78	6,10
		26,00	4,00	26,45	1,79	5,90
		30,00	2,00	26,22	1,83	6,80
		34,00	3,00	26,93	1,85	6,80
5	31,00	22,00	1,00	25,86	1,82	5,00
		28,00	1,00	26,57	1,84	6,40
		31,00	6,00	27,99	1,88	8,30
		36,00	,00	29,76	1,93	7,50
		40,00	,00	31,18	1,97	7,30
6	30,00	22,00	5,00	24,24	1,72	4,70
		27,00	4,00	24,98	1,75	6,70
		31,00	4,00	25,34	1,76	5,40
		35,00	5,00	26,45	1,79	7,00
		39,00	2,00	27,18	1,81	5,50
7	32,00	20,00	6,00	23,14	1,69	5,70
		24,00	6,00	23,51	1,70	6,20
		30,00	5,00	24,24	1,72	7,20
		34,00	6,00	25,71	1,77	7,50
		38,00	3,00	26,45	1,79	7,10
8	28,00	20,00	4,00	34,63	1,96	9,90
		24,00	3,00	36,51	2,01	8,40
		28,00	3,00	38,01	2,04	9,30
		33,00	4,00	39,14	2,07	7,10
		38,00	,00	39,90	2,08	7,30
9	28,00	20,00	3,00	24,24	1,72	5,90
		24,00	3,00	24,98	1,75	7,60
		28,00	3,00	26,81	1,80	7,40
		33,00	5,00	27,92	1,83	7,10
		37,00	1,00	28,28	1,84	6,30
10	30,00	23,00	3,00	22,49	1,75	6,80
		27,00	4,00	23,18	1,77	7,50
		31,00	6,00	23,53	1,79	6,40
		35,00	6,00	24,57	1,82	6,60
		39,00	6,00	25,26	1,84	7,10

11	37,00	22,00	6,00	25,80	1,94	7,00
		27,00	2,00	27,10	1,99	7,50
		31,00	2,00	27,43	2,00	9,00
		36,00	2,00	28,08	2,01	7,20
		39,00	2,00	28,41	2,02	6,40
12	30,00	21,00	1,00	28,13	1,75	6,50
		25,00	,00	29,30	1,78	6,70
		27,00	2,00	29,69	1,79	6,80
		31,00	5,00	31,25	1,83	6,30
		35,00	6,00	31,64	1,84	6,40
13	23,00	22,00	3,00	31,41	2,07	10,10
		26,00	3,00	32,08	2,09	7,90
		30,00	3,00	33,75	2,13	8,50
		34,00	4,00	34,75	2,16	9,20
		38,00	4,00	35,75	2,19	8,80
14	24,00	22,00	3,00	22,15	1,74	5,20
		25,00	3,00	22,49	1,75	5,00
		35,00	2,00	22,49	1,75	4,60
15	28,00	20,00	6,00	24,61	1,65	6,30
		25,00	,00	24,61	1,65	5,90
		29,00	6,00	25,00	1,66	6,40
		34,00	,00	25,39	1,67	6,40
		37,00	2,00	26,56	1,71	7,60
16	38,00	22,00	,00	22,66	1,60	4,80
		25,00	6,00	23,44	1,62	4,00
		31,00	1,00	25,00	1,66	5,20
		36,00	,00	25,78	1,68	5,60
17	23,00	20,00	4,00	29,36	1,83	7,40
		24,00	5,00	30,49	1,86	6,20
		28,00	5,00	31,62	1,89	7,30
		32,00	6,00	32,74	1,92	7,80
		35,00	2,00	33,50	1,94	7,00
18	30,00	21,00	3,00	27,68	1,91	9,30
		25,00	3,00	28,72	1,94	7,80
		29,00	5,00	29,07	1,95	9,00
		33,00	5,00	29,76	1,97	10,00
		36,00	,00	30,10	1,98	9,30
19	36,00	20,00	5,00	23,88	1,80	6,40
		24,00	5,00	24,91	1,83	6,30
		29,00	,00	25,95	1,86	6,80
		33,00	1,00	25,95	1,86	8,60
		38,00	5,00	27,34	1,90	7,60
20	27,00	20,00	6,00	24,09	1,77	7,20
		24,00	6,00	25,51	1,81	7,20
		28,00	6,00	26,22	1,83	7,20
		32,00	2,00	26,57	1,84	8,40
		38,00	3,00	28,34	1,89	5,30
21	29,00	21,00	3,00	23,34	1,66	4,80
		24,00	5,00	23,34	1,66	6,10
		29,00	1,00	24,09	1,68	6,10

		32,00	6,00	24,46	1,69	6,40
22	22,00	20,00	6,00	21,72	1,77	6,70
		24,00	5,00	22,10	1,78	6,20
		28,00	5,00	22,39	1,79	5,80
		32,00	6,00	23,05	1,82	7,20
		38,00	,00	24,39	1,86	6,30
23	26,00	22,00	4,00	21,08	1,59	7,50
		28,00	5,00	22,20	1,63	6,00
		33,00	,00	22,58	1,64	6,70
		38,00	6,00	24,84	1,70	7,50
24	31,00	22,00	6,00	34,01	2,04	8,50
		26,00	5,00	34,00	2,04	8,10
		30,00	5,00	34,72	2,06	8,40
		39,00	,00	36,85	2,12	9,00
25	32,00	21,00	6,00	21,30	1,63	4,80
		26,00	2,00	22,00	1,66	4,00
		30,00	2,00	22,77	1,68	5,00
		32,00	6,00	23,14	1,69	5,00
		37,00	6,00	23,88	1,71	4,40
26	26,00	22,00	5,00	30,12	1,94	7,10
		26,00	6,00	30,50	1,95	6,90
		30,00	3,00	31,89	1,99	8,20
		37,00	6,00	31,89	1,99	7,10
27	23,00	23,00	4,00	37,88	2,29	9,70
		27,00	4,00	38,90	2,31	10,30
		31,00	6,00	38,86	2,31	9,50
		38,00	4,00	38,86	2,31	8,40
28	33,00	23,00	1,00	27,76	2,01	8,00
		26,00	6,00	28,70	2,04	8,80
		31,00	1,00	30,04	2,07	8,20
		37,00	2,00	31,02	2,10	8,40
29	26,00	22,00	6,00	29,76	1,93	9,30
		26,00	2,00	30,47	1,95	8,30
		30,00	4,00	31,18	1,97	9,20
		36,00	3,00	33,30	2,03	8,00
30	30,00	23,00	3,00	30,10	1,98	6,30
		27,00	1,00	30,80	2,00	8,50
		30,00	3,00	31,14	2,01	8,70
		36,00	2,00	32,87	2,06	7,10
31	25,00	23,00	5,00	24,30	1,94	8,40
		27,00	3,00	24,62	1,96	6,90
		30,00	5,00	24,93	1,96	6,90
		35,00	6,00	26,51	2,02	7,10
32	21,00	20,00	5,00	30,11	1,85	6,80
		25,00	5,00	31,99	1,90	7,80
		30,00	5,00	34,25	1,95	7,60
		35,00	1,00	35,76	1,99	7,40
		38,00	1,00	38,39	2,05	8,40
33	33,00	23,00	2,00	23,51	1,87	6,20
		27,00	4,00	23,84	1,88	6,60

		31,00	2,00	24,49	1,90	6,40
		34,00	1,00	24,82	1,91	6,90
34	29,00	23,00	3,00	25,34	1,76	5,00
		27,00	3,00	25,71	1,77	5,50
		31,00	3,00	26,45	1,79	5,30
		34,00	1,00	27,55	1,82	7,60
35	28,00	23,00	5,00	24,57	1,82	6,40
		27,00	2,00	24,91	1,83	6,10
		31,00	2,00	25,26	1,84	8,00
		34,00	2,00	25,26	1,84	7,60
		39,00	1,00	26,64	1,88	7,20
36	30,00	22,00	5,00	24,61	1,73	6,60
		26,00	2,00	25,71	1,77	7,60
		30,00	2,00	27,18	1,81	7,80
		33,00	2,00	28,28	1,84	8,50
		38,00	2,00	29,38	1,87	5,60
37	20,00	23,00	1,00	27,64	1,87	7,40
		26,00	2,00	27,64	1,87	7,10
		30,00	5,00	28,34	1,89	6,80
		33,00	5,00	28,34	1,89	8,10
		38,00	1,00	29,05	1,91	6,80
38	28,00	21,00	3,00	29,36	1,83	6,70
		24,00	5,00	30,11	1,85	7,40
		28,00	3,00	31,24	1,88	7,10
		31,00	4,00	31,99	1,90	8,90
39	30,00	21,00	,00	25,56	1,98	5,30
		24,00	2,00	25,56	1,98	8,30
		28,00	2,00	25,88	1,99	7,30
		31,00	1,00	26,20	2,01	11,90
		36,00	2,00	27,77	2,06	10,90
40	37,00	21,00	2,00	29,41	1,96	8,00
		23,00	5,00	29,76	1,97	6,90
		27,00	5,00	31,14	2,01	7,70
		30,00	5,00	32,18	2,04	10,50
		35,00	5,00	33,56	2,07	7,20
41	33,00	22,00	5,00	23,51	1,70	6,50
		25,00	,00	23,51	1,70	4,70
		28,00	6,00	24,24	1,72	5,30
		31,00	6,00	24,24	1,72	6,50
		36,00	4,00	24,98	1,75	6,00
42	26,00	21,00	,00	26,45	1,79	6,50
		25,00	,00	26,81	1,80	6,30
		27,00	6,00	27,55	1,82	5,10
		33,00	,00	28,28	1,84	7,00
43	33,00	23,00	5,00	33,98	1,89	7,50
		27,00	5,00	34,77	1,91	8,50
		30,00	4,00	35,55	1,93	7,00
		35,00	5,00	36,72	1,96	7,20
44	31,00	23,00	2,00	22,09	1,87	6,10
		28,00	,00	23,04	1,90	6,70

		33,00	,00	23,36	1,91	6,90
45	32,00	22,00	4,00	28,65	1,85	6,60
		27,00	2,00	30,12	1,89	7,30
		32,00	1,00	31,59	1,93	8,70
		38,00	1,00	32,69	1,96	8,80
46	31,00	22,00	2,00	32,74	1,92	7,00
		26,00	6,00	33,50	1,94	6,80
		30,00	6,00	34,63	1,96	8,10
		36,00	6,00	36,13	2,00	8,90
47	29,00	21,00	,00	22,84	1,76	6,90
		25,00	1,00	23,88	1,80	6,00
		28,00	6,00	24,91	1,83	7,60
		34,00	6,00	26,64	1,88	6,50
		38,00	6,00	27,34	1,90	8,30
48	28,00	23,00	2,00	28,28	1,84	7,70
		27,00	3,00	29,75	1,88	8,40
		31,00	2,00	30,12	1,89	7,70
		37,00	2,00	31,96	1,94	8,40
49	25,00	23,00	5,00	20,31	1,52	5,20
		27,00	3,00	21,48	1,56	5,80
		30,00	4,00	21,83	1,62	6,40
		36,00	4,00	24,61	1,65	6,40
50	25,00	23,00	6,00	26,22	1,83	6,40
		26,00	2,00	26,57	1,84	7,20
		30,00	,00	27,99	1,88	8,60
		36,00	,00	29,41	1,92	6,80
51	35,00	23,00	3,00	21,83	1,62	5,40
		25,00	6,00	21,83	1,62	5,50
		29,00	1,00	22,58	1,64	6,50
		35,00	,00	23,34	1,66	6,40
		39,00	,00	24,09	1,68	5,50
52	24,00	23,00	3,00	24,09	1,68	4,80
		28,00	2,00	25,59	1,73	5,70
		34,00	2,00	26,72	1,76	5,60
		38,00	2,00	27,10	1,77	7,00
53	30,00	23,00	4,00	20,31	1,52	5,80
		28,00	3,00	22,27	1,58	6,60
		34,00	3,00	23,83	1,63	5,90
		38,00	1,00	26,17	1,69	6,70
54	25,00	23,00	4,00	21,11	1,70	6,60
		28,00	4,00	21,80	1,73	4,70
		34,00	3,00	22,49	1,75	4,90
		38,00	3,00	23,18	1,77	5,70
55	29,00	23,00	1,00	26,72	1,76	6,70
		27,00	6,00	27,48	1,78	6,80
		33,00	6,00	28,23	1,80	6,60
		37,00	6,00	28,60	1,81	6,60
56	28,00	23,00	,00	28,52	1,76	7,60
		27,00	2,00	28,91	1,77	6,40
		33,00	6,00	30,08	1,80	7,80

		37,00	2,00	31,25	1,83	7,10
57	26,00	23,00	1,00	23,51	1,70	8,00
		26,00	2,00	24,24	1,72	8,00
		32,00	2,00	26,08	1,78	11,00
		36,00	2,00	27,92	1,83	6,70
58	25,00	22,00	6,00	20,07	1,67	6,10
		26,00	4,00	20,76	1,69	6,60
		30,00	,00	21,45	1,71	7,50
		35,00	,00	22,15	1,74	7,30
		38,00	6,00	23,20	1,77	7,10
59	29,00	23,00	4,00	29,38	1,87	5,60
		26,00	4,00	30,12	1,89	5,80
		33,00	1,00	31,96	1,94	4,30
60	32,00	22,00	,00	28,72	1,94	7,80
		24,00	1,00	29,07	1,95	8,20
		30,00	5,00	31,49	2,02	10,70
		35,00	5,00	32,18	2,04	12,00
61	29,00	21,00	4,00	21,67	1,64	6,10
		25,00	4,00	22,04	1,66	5,60
		30,00	2,00	22,04	1,66	6,00
		35,00	2,00	22,77	1,68	5,90
62	39,00	21,00	6,00	27,18	1,81	7,40
		27,00	,00	28,28	1,84	10,50
		32,00	,00	29,38	1,87	8,80
		36,00	2,00	30,12	1,89	8,70
		40,00	1,00	30,85	1,91	9,00
63	32,00	23,00	1,00	22,27	1,58	3,90
		27,00	6,00	23,05	1,61	4,40
		32,00	2,00	23,44	1,62	4,30
		35,00	6,00	23,83	1,63	4,60
64	33,00	22,00	1,00	26,81	1,80	7,30
		27,00	1,00	27,55	1,82	5,80
		31,00	1,00	27,92	1,83	6,40
		36,00	1,00	27,92	1,83	7,40
65	29,00	23,00	6,00	23,88	1,80	6,30
		27,00	6,00	24,22	1,81	6,90
		31,00	6,00	24,91	1,83	6,10
		35,00	6,00	25,95	1,86	7,50
		39,00	6,00	26,64	1,88	8,00
66	26,00	21,00	5,00	26,64	1,88	6,60
		25,00	6,00	26,64	1,91	6,70
		30,00	1,00	27,68	1,91	7,50
		34,00	1,00	28,37	1,93	8,00
		38,00	,00	28,03	1,92	7,60
67	25,00	23,00	3,00	26,08	1,78	6,50
		27,00	,00	26,81	1,80	6,40
		35,00	,00	28,65	1,85	5,10
		38,00	2,00	29,75	1,88	5,00
68	21,00	22,00	2,00	30,69	2,09	9,60
		26,00	5,00	32,00	2,13	10,90

		29,00	6,00	32,65	2,15	11,50
		33,00	6,00	34,61	2,20	11,20
		38,00	6,00	36,57	2,25	9,60
69	32,00	21,00	5,00	23,53	1,79	7,00
		25,00	1,00	24,57	1,82	8,00
		29,00	,00	24,91	1,83	8,30
		33,00	6,00	25,61	1,85	7,90
		37,00	6,00	26,64	1,88	8,90
70	29,00	20,00	2,00	21,45	1,71	6,30
		24,00	3,00	22,15	1,74	5,90
		28,00	5,00	23,18	1,77	6,90
		33,00	6,00	24,22	1,81	6,20
		38,00	2,00	24,91	1,83	5,20
71	31,00	20,00	1,00	21,45	1,60	5,80
		25,00	2,00	22,58	1,64	4,80
		29,00	4,00	22,96	1,65	4,90
		33,00	4,00	23,71	1,67	4,30
		37,00	4,00	24,84	1,70	4,50
72	28,00	22,00	6,00	22,77	1,68	6,10
		28,00	,00	24,24	1,72	6,50
		30,00	6,00	24,61	1,73	6,40
		35,00	,00	25,71	1,77	7,50
73	29,00	22,00	5,00	24,06	1,85	9,80
		28,00	,00	25,06	1,88	8,80
		31,00	6,00	24,73	1,87	8,00
		36,00	1,00	24,73	1,87	8,70
		39,00	6,00	25,73	1,90	7,20
74	25,00	21,00	6,00	22,68	1,72	6,20
		26,00	3,00	22,68	1,74	7,00
		30,00	6,00	24,09	1,77	7,10
		34,00	5,00	24,80	1,79	6,60
		38,00	5,00	25,51	1,81	6,40
75	22,00	21,00	2,00	25,78	1,68	5,50
		25,00	6,00	26,95	1,72	6,30
		29,00	2,00	26,95	1,72	6,20
76	27,00	20,00	1,00	22,49	1,75	6,30
		24,00	3,00	23,18	1,77	6,90
		28,00	2,00	23,88	1,80	7,90
		32,00	3,00	24,57	1,82	6,80
		36,00	3,00	25,26	1,84	6,00
77	30,00	21,00	6,00	24,49	1,90	8,20
		25,00	6,00	24,82	1,91	8,90
		31,00	6,00	26,45	1,96	8,40
		35,00	6,00	27,73	1,97	10,20
		39,00	2,00	27,43	2,00	10,00
78	22,00	22,00	4,00	22,96	1,65	4,60
		27,00	2,00	23,34	1,66	4,60
		32,00	5,00	25,59	1,73	6,00
		36,00	5,00	27,10	1,77	6,20
		40,00	5,00	28,23	1,80	6,40

79	34,00	22,00	4,00	26,64	1,88	7,40
		26,00	5,00	26,64	1,88	6,40
		33,00	3,00	26,99	1,89	6,40
		37,00	3,00	27,34	1,90	6,90
		40,00	3,00	27,68	1,91	6,70
80	32,00	21,00	6,00	24,98	1,75	5,80
		26,00	4,00	24,98	1,75	7,30
		31,00	4,00	26,08	1,78	5,90
		35,00	6,00	26,81	1,80	7,10
		39,00	,00	27,92	1,83	6,10
81	30,00	20,00	6,00	25,56	1,98	6,10
		25,00	,00	26,20	2,01	5,90
		30,00	,00	26,83	2,03	6,60
		34,00	,00	27,14	2,04	5,80
		37,00	6,00	28,09	2,07	5,70
82	36,00	22,00	3,00	33,63	2,18	9,40
		26,00	,00	35,27	2,22	10,00
		32,00	,00	37,22	2,27	10,60
		36,00	,00	38,20	2,30	10,60
		40,00	,00	38,83	2,31	10,50
83	36,00	21,00	3,00	22,39	1,79	5,70
		25,00	4,00	22,39	1,79	6,50
		30,00	3,00	23,05	1,82	5,50
		34,00	3,00	23,72	1,84	5,70
		38,00	2,00	24,73	1,87	6,60
84	29,00	21,00	,00	21,23	1,47	5,10
		24,00	6,00	22,48	1,51	4,60
		29,00	6,00	22,89	1,52	4,80
		33,00	6,00	23,73	1,54	5,90
		37,00	6,00	24,56	1,57	4,50
85	26,00	21,00	4,00	23,12	1,56	4,10
		25,00	4,00	23,94	1,59	4,60
		29,00	6,00	23,94	1,59	3,90
		33,00	6,00	25,15	1,62	4,20
		36,00	6,00	25,15	1,62	4,10
86	36,00	23,00	2,00	28,52	1,76	4,90
		28,00	6,00	29,69	1,79	7,60
		32,00	6,00	30,08	1,80	6,30
		36,00	6,00	31,25	1,83	6,30
87	26,00	23,00	4,00	25,59	1,73	5,10
		28,00	5,00	26,72	1,76	7,10
		32,00	6,00	27,85	1,79	5,70
		36,00	6,00	28,98	1,82	5,20
88	32,00	23,00	3,00	24,22	1,81	5,80
		29,00	2,00	24,91	1,83	7,70
		33,00	1,00	24,91	1,83	7,10
		36,00	5,00	26,30	1,87	8,00
		40,00	2,00	26,30	1,87	8,00
89	35,00	21,00	5,00	23,18	1,77	5,60
		26,00	3,00	24,57	1,82	7,50

		29,00	6,00	25,26	1,84	6,40
		32,00	5,00	26,30	1,87	7,30
		35,00	4,00	26,64	1,88	7,40
90	33,00	22,00	6,00	29,69	1,79	5,90
		26,00	6,00	30,08	1,80	5,70
		30,00	6,00	30,08	1,80	6,80
		34,00	6,00	31,25	1,83	6,40
91	24,00	23,00	2,00	23,05	1,61	4,50
		27,00	2,00	23,83	1,63	5,80
		32,00	4,00	25,00	1,66	5,30
		36,00	2,00	25,78	1,68	5,00
92	33,00	22,00	,00	28,70	1,90	6,70
		26,00	,00	29,41	1,92	7,30
		30,00	,00	30,47	1,95	7,30
		34,00	,00	31,53	1,98	7,20
		38,00	,00	31,90	1,99	7,20
93	35,00	22,00	4,00	24,06	1,85	6,50
		26,00	1,00	24,06	1,85	6,50
		31,00	1,00	24,39	1,86	5,40
		36,00	,00	25,39	1,89	7,00
94	30,00	23,00	,00	23,34	1,66	5,50
		27,00	3,00	24,09	1,68	5,90
		31,00	2,00	24,46	1,69	5,60
		35,00	2,00	25,59	1,73	6,60
		39,00	2,00	25,97	1,74	5,00
95	32,00	21,00	6,00	30,39	1,72	5,40
		26,00	1,00	31,22	1,74	5,20
		30,00	,00	32,47	1,77	6,40
		34,00	2,00	33,71	1,80	5,80
		38,00	2,00	34,13	1,80	5,60
96	34,00	22,00	3,00	26,08	1,78	5,90
		26,00	3,00	26,45	1,79	5,30
		30,00	3,00	27,55	1,82	6,60
97	25,00	22,00	1,00	26,17	1,69	5,60
		27,00	1,00	27,34	1,73	5,20
		31,00	1,00	28,13	1,75	6,20
		34,00	5,00	28,91	1,77	5,80
		37,00	5,00	29,30	1,78	5,60
98	24,00	21,00	,00	22,04	1,66	6,30
		25,00	3,00	22,77	1,68	7,30
		30,00	3,00	23,88	1,71	7,40
		33,00	3,00	24,61	1,73	7,70

CI_A	HR_A	BPS_A	BPD_A	MAP_A	SVR_A	SVRI_A
4,10	75,00	129,00	83,00	98,00	980,00	1840,00
3,90	69,00	118,00	74,00	89,00	920,00	1750,00
4,20	73,00	118,00	70,00	86,00	810,00	1550,00
4,20	77,00	119,00	85,00	96,00	890,00	1740,00
3,70	80,00	123,00	91,00	102,00	1060,00	2110,00
4,70	111,00	119,00	71,00	87,00	860,00	1400,00
4,60	120,00	119,00	80,00	93,00	940,00	1550,00
3,90	102,00	119,00	76,00	90,00	1040,00	1750,00
5,00	119,00	126,00	88,00	101,00	920,00	1560,00
4,50	118,00	116,00	84,00	95,00	950,00	1630,00
3,70	85,00	100,00	63,00	75,00	830,00	1540,00
3,70	81,00	107,00	59,00	75,00	820,00	1550,00
3,20	80,00	106,00	67,00	80,00	1000,00	1910,00
3,20	75,00	115,00	79,00	91,00	1120,00	2140,00
3,20	83,00	118,00	83,00	95,00	1180,00	2290,00
3,40	86,00	115,00	79,00	91,00	1140,00	2040,00
3,30	83,00	100,00	76,00	84,00	1090,00	1950,00
3,70	84,00	115,00	80,00	92,00	1050,00	1930,00
3,70	76,00	107,00	73,00	84,00	940,00	1740,00
2,70	67,00	91,00	62,00	72,00	1080,00	1980,00
3,50	102,00	103,00	68,00	80,00	950,00	1750,00
4,40	92,00	107,00	75,00	86,00	800,00	1500,00
3,90	91,00	107,00	79,00	88,00	900,00	1740,00
3,70	86,00	119,00	87,00	98,00	1030,00	2040,00
2,70	78,00	105,00	80,00	88,00	1440,00	2490,00
3,80	84,00	102,00	71,00	81,00	920,00	1610,00
3,10	71,00	95,00	65,00	75,00	1050,00	1850,00
3,90	76,00	109,00	84,00	92,00	1030,00	1840,00
3,00	69,00	123,00	84,00	97,00	1360,00	2470,00
3,40	78,00	103,00	71,00	82,00	1090,00	1860,00
3,60	86,00	107,00	77,00	87,00	1070,00	1820,00
4,20	92,00	106,00	81,00	89,00	950,00	1640,00
4,20	113,00	115,00	83,00	94,00	960,00	1710,00
4,00	95,00	119,00	83,00	95,00	1020,00	1840,00
5,00	126,00	131,00	89,00	103,00	800,00	1570,00
4,20	90,00	120,00	79,00	93,00	850,00	1710,00
4,50	94,00	113,00	80,00	91,00	750,00	1540,00
3,40	89,00	112,00	78,00	89,00	960,00	1980,00
3,50	100,00	124,00	85,00	98,00	1030,00	2150,00
3,40	72,00	102,00	63,00	76,00	980,00	1690,00
4,30	91,00	102,00	73,00	83,00	830,00	1460,00
4,10	84,00	103,00	72,00	82,00	840,00	1510,00
3,90	96,00	107,00	73,00	84,00	890,00	1640,00
3,40	82,00	104,00	73,00	83,00	1000,00	1840,00
3,90	85,00	98,00	66,00	77,00	850,00	1500,00
4,20	77,00	97,00	62,00	74,00	740,00	1320,00
3,60	64,00	96,00	61,00	73,00	860,00	1540,00
3,60	79,00	102,00	75,00	84,00	970,00	1770,00
3,90	92,00	97,00	79,00	85,00	910,00	1680,00

3,60	59,00	96,00	71,00	79,00	870,00	1690,00
3,80	70,00	95,00	66,00	76,00	760,00	1520,00
4,50	77,00	101,00	64,00	76,00	640,00	1280,00
3,60	62,00	101,00	69,00	80,00	840,00	1710,00
3,20	61,00	105,00	75,00	85,00	1000,00	2040,00
3,70	85,00	100,00	65,00	77,00	900,00	1570,00
3,70	80,00	92,00	61,00	71,00	870,00	1560,00
3,80	91,00	93,00	66,00	75,00	830,00	1490,00
3,40	85,00	99,00	62,00	74,00	890,00	1630,00
3,50	92,00	99,00	66,00	77,00	920,00	1690,00
4,90	88,00	115,00	64,00	81,00	610,00	1270,00
3,80	85,00	110,00	81,00	91,00	880,00	1850,00
4,00	86,00	105,00	69,00	81,00	720,00	1540,00
4,30	104,00	114,00	72,00	86,00	700,00	1520,00
4,00	118,00	107,00	66,00	80,00	690,00	1520,00
3,00	66,00	92,00	60,00	71,00	1020,00	1780,00
2,90	59,00	94,00	58,00	70,00	1050,00	1840,00
2,60	57,00	105,00	72,00	83,00	1370,00	2400,00
3,80	86,00	107,00	75,00	86,00	1030,00	1710,00
3,50	91,00	105,00	76,00	86,00	1110,00	1840,00
3,80	93,00	108,00	75,00	86,00	1020,00	1710,00
3,80	88,00	112,00	79,00	90,00	1080,00	1810,00
4,40	97,00	106,00	80,00	89,00	910,00	1550,00
3,00	79,00	91,00	67,00	75,00	1190,00	1900,00
2,50	70,00	93,00	59,00	70,00	1320,00	2140,00
3,10	91,00	92,00	66,00	75,00	1080,00	1810,00
3,30	94,00	95,00	67,00	76,00	1020,00	1730,00
4,00	84,00	111,00	72,00	85,00	880,00	1610,00
3,30	74,00	104,00	71,00	82,00	1010,00	1880,00
3,80	90,00	105,00	69,00	81,00	850,00	1600,00
4,10	87,00	110,00	77,00	88,00	860,00	1650,00
3,60	102,00	115,00	74,00	88,00	970,00	1880,00
4,80	103,00	107,00	73,00	84,00	690,00	1320,00
4,00	85,00	107,00	69,00	82,00	800,00	1560,00
4,60	104,00	108,00	79,00	89,00	760,00	1490,00
5,10	118,00	114,00	76,00	89,00	680,00	1340,00
4,70	102,00	117,00	84,00	95,00	790,00	1560,00
3,50	81,00	101,00	66,00	78,00	930,00	1670,00
3,40	86,00	108,00	67,00			
3,60	85,00	113,00	80,00	91,00	1010,00	1880,00
4,60	103,00	106,00	74,00	85,00	760,00	1410,00
4,00	104,00	104,00	77,00	86,00	860,00	1650,00
4,10	85,00	106,00	69,00	81,00	850,00	1510,00
4,00	82,00	109,00	75,00	86,00	910,00	1650,00
3,90	82,00	97,00	70,00	79,00	840,00	1540,00
4,50	95,00	107,00	72,00	84,00	760,00	1410,00
2,80	85,00	101,00	76,00	84,00	1210,00	2300,00
2,90	63,00	98,00	62,00	74,00	1180,00	1960,00
3,60	94,00	93,00	64,00	74,00	920,00	1540,00
3,60	91,00	99,00	68,00	78,00	980,00	1660,00

3,80	112,00	96,00	73,00	81,00	960,00	1640,00
3,80	75,00	105,00	65,00	78,00	880,00	1550,00
3,40	74,00	108,00	64,00	79,00	980,00	1740,00
3,20	64,00	109,00	70,00	83,00	1080,00	1950,00
4,00	79,00	115,00	74,00	88,00	920,00	1680,00
3,40	71,00	114,00	84,00	94,00	1130,00	2120,00
4,70	109,00	94,00	68,00	77,00	780,00	1240,00
3,70	81,00	89,00	60,00	70,00	880,00	1440,00
4,10	91,00	86,00	68,00	74,00	840,00	1370,00
4,40	104,00	91,00	69,00	76,00	770,00	1310,00
4,10	93,00	111,00	65,00	80,00	720,00	1470,00
3,90	82,00	112,00	60,00	77,00	730,00	1490,00
4,10	74,00	118,00	67,00	84,00	760,00	1570,00
4,20	83,00	123,00	76,00	92,00	800,00	1690,00
3,00	70,00	124,00	83,00	97,00	1540,00	2530,00
2,40	55,00	120,00	67,00	85,00	1620,00	2690,00
2,90	70,00	116,00	75,00	89,00	1370,00	2300,00
3,00	67,00	120,00	77,00	91,00	1390,00	2360,00
2,50	85,00	119,00	88,00	98,00	1730,00	2970,00
3,70	76,00	105,00	74,00	84,00	890,00	1740,00
3,50	96,00	105,00	78,00	87,00	970,00	1910,00
4,10	99,00	106,00	70,00	82,00	750,00	1490,00
3,60	93,00	101,00	79,00	86,00	920,00	1840,00
4,20	79,00	107,00	70,00	82,00	640,00	1470,00
4,40	84,00	109,00	72,00	84,00	620,00	1440,00
4,10	76,00	112,00	66,00	81,00	650,00	1520,00
3,60	74,00	98,00	68,00	78,00	710,00	1640,00
4,00	90,00					
4,30	82,00	100,00	67,00	78,00	680,00	1380,00
3,90	82,00	101,00	73,00	82,00	770,00	1590,00
4,00	96,00	98,00	68,00	78,00	700,00	1470,00
4,80	98,00	111,00	71,00	84,00	690,00	1340,00
4,20	89,00	111,00	77,00	88,00	810,00	1590,00
4,60	90,00	113,00	74,00	87,00	720,00	1430,00
3,90	74,00	111,00	78,00	89,00	850,00	1730,00
3,20	81,00	109,00	68,00	82,00	990,00	1970,00
4,20	94,00	134,00	89,00	104,00	940,00	1890,00
4,30	90,00	128,00	83,00	98,00	860,00	1740,00
3,40	82,00	115,00	75,00	88,00	950,00	1950,00
4,30	75,00	128,00	70,00	89,00	810,00	1570,00
3,50	68,00	107,00	70,00	82,00	910,00	1780,00
3,50	70,00	106,00	71,00	83,00	920,00	1810,00
3,50	77,00	109,00	74,00	86,00	920,00	1860,00
3,70	85,00	113,00	74,00	87,00	980,00	1810,00
4,10	92,00	117,00	77,00	90,00	880,00	1680,00
3,90	94,00	113,00	83,00	93,00	950,00	1860,00
3,70	92,00	111,00	80,00	90,00	930,00	1860,00
4,10	99,00	110,00	77,00	88,00	800,00	1640,00
3,30	71,00	111,00	81,00	91,00	1120,00	2090,00
3,50	80,00	99,00	74,00	82,00	930,00	1760,00

3,40	72,00	102,00	69,00	80,00	940,00	1800,00
3,60	73,00	105,00	75,00	85,00	940,00	1800,00
2,90	64,00	102,00	62,00	75,00	1130,00	2000,00
3,10	77,00	95,00	63,00	74,00	1030,00	1820,00
2,90	61,00	109,00	62,00	78,00	1120,00	2010,00
4,10	82,00	111,00	67,00	82,00	830,00	1510,00
3,50	75,00	104,00	65,00	78,00	930,00	1690,00
3,30	76,00	99,00	62,00	74,00	910,00	1670,00
4,30	95,00	104,00	73,00	83,00	790,00	1460,00
4,10	131,00	95,00	64,00	74,00	740,00	1370,00
3,80	108,00	106,00	73,00	84,00	890,00	1670,00
3,80	94,00	95,00	66,00	76,00	870,00	1510,00
4,30	97,00	92,00	67,00	75,00	750,00	1330,00
4,30	98,00	109,00	75,00	86,00	840,00	1520,00
4,60	110,00	97,00	70,00	79,00	710,00	1310,00
3,00	89,00	113,00	80,00	91,00	1240,00	2320,00
3,90	79,00	104,00	61,00	75,00	770,00	1450,00
3,80	82,00	98,00	63,00	75,00	800,00	1500,00
3,60	77,00	98,00	60,00	73,00	810,00	1550,00
4,30	101,00	100,00	64,00	76,00	710,00	1350,00
3,60	79,00	92,00	69,00	77,00	860,00	1640,00
3,70	86,00	105,00	73,00	84,00	950,00	1740,00
4,00	94,00	110,00	70,00	83,00	850,00	1570,00
3,80	86,00	109,00	77,00	88,00	950,00	1790,00
4,70	107,00	107,00	78,00	88,00	760,00	1440,00
2,70	78,00	96,00	67,00	77,00	1100,00	2190,00
4,10	77,00	99,00	68,00	78,00	720,00	1430,00
3,60	83,00	100,00	69,00	79,00	820,00	1650,00
5,90	118,00	114,00	77,00	89,00	570,00	1150,00
5,30	106,00	115,00	83,00	94,00	660,00	1370,00
4,00	84,00	97,00	64,00	75,00	680,00	1330,00
3,50	73,00	100,00	63,00	75,00	820,00	1630,00
3,80	80,00	103,00	68,00	80,00	790,00	1580,00
5,10	95,00	113,00	70,00	84,00	610,00	1250,00
3,50	73,00	110,00	72,00	85,00	900,00	1860,00
3,80	88,00	95,00	67,00	76,00	890,00	1520,00
2,70	67,00	89,00	57,00	68,00	1110,00	1890,00
3,10	76,00	95,00	58,00	70,00	990,00	1710,00
3,80	97,00	90,00	63,00	72,00	840,00	1450,00
3,40	101,00	91,00	63,00	72,00	920,00	1610,00
3,60	77,00	96,00	56,00	69,00	810,00	1460,00
3,50	91,00	99,00	58,00	72,00	860,00	1550,00
2,80	72,00	99,00	55,00	70,00	1060,00	1930,00
3,80	89,00	95,00	62,00	73,00	780,00	1450,00
3,90	81,00	103,00	62,00	76,00	770,00	1470,00
4,40	104,00	112,00	70,00	84,00	750,00	1450,00
3,60	87,00	93,00	60,00	71,00	750,00	1460,00
3,70	103,00	95,00	67,00	76,00	810,00	1600,00
3,20	75,00	113,00	76,00	88,00	1090,00	2050,00
3,50	82,00	114,00	75,00	88,00	1000,00	1900,00

3,60	87,00	108,00	81,00	90,00	990,00	1900,00
3,50	70,00	116,00	73,00	87,00	1000,00	1860,00
3,90	78,00	103,00	58,00	73,00	760,00	1440,00
4,50	89,00	105,00	73,00	84,00	740,00	1420,00
4,50	83,00	111,00	70,00	84,00	720,00	1420,00
3,60	69,00	109,00	64,00	79,00	860,00	1640,00
3,50	67,00	104,00	64,00	77,00	860,00	1670,00
4,10	92,00	115,00	78,00	90,00	850,00	1670,00
4,40	111,00	112,00	72,00	85,00	730,00	1470,00
3,90	90,00	115,00	74,00	88,00	970,00	1710,00
3,40	78,00	108,00	70,00	83,00	1050,00	1860,00
4,10	85,00	108,00	76,00	87,00	870,00	1590,00
3,40	85,00	115,00	80,00	92,00	1090,00	2050,00
4,30	92,00	118,00	86,00	97,00	900,00	1720,00
4,10	84,00	104,00	73,00	83,00	830,00	1520,00
4,50	90,00	110,00	78,00	89,00	810,00	1520,00
4,10	85,00	106,00	71,00	83,00	820,00	1550,00
4,30	108,00	103,00	81,00	88,00	800,00	1560,00
3,40	94,00	100,00	70,00	80,00	1150,00	1760,00
3,70	106,00	109,00	74,00	86,00	1130,00	1770,00
3,90	112,00	102,00	71,00	81,00	970,00	1560,00
3,90	113,00	110,00	78,00	89,00	1060,00	1760,00
3,50	73,00	105,00	67,00	80,00	950,00	1750,00
3,90	79,00	97,00	62,00	74,00	780,00	1440,00
4,50	98,00	100,00	65,00	77,00	680,00	1280,00
3,50	64,00	113,00	72,00	86,00	960,00	1850,00
3,30	71,00	89,00	55,00	66,00	920,00	1490,00
3,40	70,00	92,00	58,00	69,00	950,00	1530,00
4,00	78,00	96,00	59,00	71,00	820,00	1350,00
3,80	101,00	105,00	69,00	81,00	960,00	1600,00
3,30	74,00	97,00	63,00	74,00	1020,00	1720,00
2,80	89,00	94,00	63,00	73,00	1150,00	1950,00
3,30	126,00	99,00	69,00	79,00	1040,00	1790,00
3,20	118,00	94,00	71,00	79,00	1060,00	1870,00
4,00	134,00	99,00	67,00	78,00	850,00	1500,00
3,80	108,00	94,00	65,00	75,00	970,00	1480,00
4,20	101,00	102,00	66,00	78,00	890,00	1420,00
3,60	103,00	103,00	80,00	88,00	1130,00	1850,00
4,00	115,00	109,00	80,00	90,00	1030,00	1760,00
3,80	77,00	95,00	64,00	74,00	850,00	1450,00
2,70	73,00	88,00	64,00	72,00	1170,00	2020,00
2,80	66,00	95,00	70,00	78,00	1200,00	2120,00
3,20	75,00	106,00	69,00	81,00	1070,00	1910,00
3,80	98,00	97,00	68,00	78,00	880,00	1550,00
3,80	104,00	94,00	70,00	78,00	870,00	1560,00
3,70	100,00	98,00	73,00	81,00	930,00	1680,00
3,60	87,00	98,00	67,00	77,00	890,00	1610,00
4,30	103,00	114,00	83,00	93,00	930,00	1650,00
3,60	98,00	116,00	76,00	89,00	1070,00	1890,00
4,30	111,00	137,00	92,00	107,00	1060,00	1920,00

3,90	109,00	127,00	93,00	104,00	1130,00	2080,00
4,70	117,00	107,00	82,00	90,00	860,00	1470,00
4,60	123,00	112,00	89,00	97,00	930,00	1610,00
6,10	150,00	118,00	85,00	96,00	690,00	1230,00
3,70	123,00	112,00	84,00	93,00	1090,00	1990,00
3,60	84,00	102,00	65,00	77,00	960,00	1610,00
3,90	82,00	112,00	74,00	87,00	1010,00	1720,00
4,30	106,00	112,00	82,00	92,00	940,00	1620,00
4,20	91,00	100,00	73,00	82,00	860,00	1490,00
4,00	94,00	106,00	71,00	83,00	900,00	1600,00
3,00	89,00	100,00	76,00	84,00	1140,00	2130,00
3,10	102,00	98,00	70,00	79,00	1020,00	1940,00
2,20	106,00	111,00	82,00	92,00	1650,00	3210,00
4,00	81,00	104,00	70,00	81,00	790,00	1550,00
4,20	76,00	113,00	69,00	84,00	780,00	1540,00
5,30	95,00	120,00	82,00	95,00	680,00	1380,00
5,90	106,00	113,00	78,00	90,00	570,00	1170,00
3,70	88,00	100,00	72,00	81,00	1010,00	1670,00
3,40	92,00	93,00	67,00	76,00	1030,00	1720,00
3,60	99,00	95,00	75,00	82,00	1030,00	1700,00
3,50	91,00	95,00	70,00	78,00	1000,00	1690,00
4,10	79,00	94,00	54,00	67,00	690,00	1240,00
5,70	112,00	106,00	64,00	78,00	560,00	1030,00
4,70	79,00	101,00	50,00	67,00	580,00	1080,00
4,60	88,00	88,00	58,00	68,00	590,00	1110,00
4,70	85,00	96,00	60,00	72,00	600,00	1150,00
2,50	58,00	96,00	63,00	74,00	1420,00	2250,00
2,70	74,00	91,00	63,00	72,00	1240,00	2000,00
2,60	64,00	87,00	67,00	74,00	1310,00	2130,00
2,80	76,00	93,00	67,00	76,00	1240,00	2030,00
4,00	85,00	105,00	74,00	84,00	880,00	1600,00
3,20	82,00	101,00	69,00	80,00	1050,00	1920,00
3,50	74,00	96,00	60,00	72,00	850,00	1570,00
4,10	83,00	103,00	76,00	85,00	870,00	1590,00
3,50	77,00	101,00	63,00	76,00	920,00	1650,00
3,80	77,00	97,00	61,00	73,00	800,00	1440,00
3,30	80,00	93,00	68,00	76,00	950,00	1730,00
4,00	90,00	106,00	73,00	84,00	850,00	1590,00
4,20	92,00	106,00	78,00	87,00	830,00	1560,00
3,50	79,00	93,00	64,00	74,00	850,00	1600,00
3,50	81,00	89,00	57,00	68,00	750,00	1430,00
3,90	82,00	93,00	64,00	74,00	750,00	1440,00
4,10	82,00	95,00	63,00	74,00	700,00	1350,00
3,90	88,00	96,00	68,00	77,00	770,00	1490,00
3,70	81,00	93,00	67,00	76,00	880,00	1560,00
3,60	88,00	91,00	66,00	74,00	870,00	1570,00
2,80	72,00	96,00	69,00	78,00	1160,00	2150,00
2,70	66,00	97,00	76,00	83,00	1260,00	2370,00
4,60	74,00	122,00	60,00	81,00	640,00	1350,00
5,10	98,00	121,00	73,00	89,00	620,00	1320,00

5,30	87,00	124,00	79,00	94,00	630,00	1350,00
5,10	81,00	118,00	75,00	89,00	610,00	1350,00
4,30	89,00	126,00	83,00	97,00	770,00	1730,00
3,90	79,00	95,00	64,00	74,00	800,00	1430,00
4,40	77,00	101,00	64,00	76,00	720,00	1310,00
4,50	77,00	103,00	66,00	78,00	720,00	1320,00
4,30	82,00	98,00	66,00	77,00	740,00	1370,00
4,70	100,00	106,00	70,00	82,00	700,00	1310,00
3,70	80,00	101,00	70,00	80,00	960,00	1650,00
3,40	80,00	101,00	70,00	80,00	1020,00	1780,00
3,90	78,00	99,00	69,00	79,00	870,00	1540,00
3,40	83,00	112,00	81,00	91,00	1130,00	2050,00
2,90	71,00	113,00	81,00	92,00	1350,00	2470,00
3,60	87,00	111,00	70,00	84,00	1090,00	1750,00
2,90	73,00	104,00	73,00	83,00	1310,00	2150,00
3,00	88,00	122,00	76,00	91,00	1420,00	2340,00
2,50	64,00	110,00	72,00	85,00	1520,00	2560,00
2,60	61,00	107,00	83,00	91,00	1560,00	2660,00
3,60	64,00	92,00	56,00	68,00	840,00	1420,00
3,80	76,00	99,00	69,00	79,00	870,00	1500,00
3,70	77,00	97,00	58,00	71,00	830,00	1450,00
4,20	84,00	100,00	67,00	78,00	790,00	1400,00
5,30	107,00	114,00	80,00	91,00	710,00	1310,00
4,70	103,00	120,00	83,00	95,00	830,00	1570,00
4,30	90,00	101,00	70,00	80,00	760,00	1420,00
4,60	106,00	113,00	85,00	94,00	830,00	1560,00
3,80	94,00	118,00	88,00	98,00	1030,00	1970,00
3,60	73,00	105,00	67,00	80,00	980,00	1690,00
4,00	86,00	92,00	66,00	75,00	800,00	1400,00
4,00	85,00	105,00	73,00	84,00	900,00	1600,00
3,70	80,00	99,00	67,00	78,00	890,00	1600,00
3,50	82,00	97,00	75,00	82,00	970,00	1760,00
3,30	82,00	109,00	72,00	84,00	1160,00	1950,00
3,60	91,00	106,00	70,00	82,00	990,00	1710,00
3,60	102,00	108,00	74,00	85,00	1040,00	1800,00
3,60	78,00	105,00	70,00	82,00	990,00	1750,00
3,90	86,00	101,00	70,00	80,00	890,00	1580,00
4,40	88,00	98,00	69,00	79,00	760,00	1370,00
3,70	83,00	99,00	74,00	82,00	920,00	1670,00
3,30	74,00	106,00	80,00	89,00	1130,00	2090,00
4,30	86,00	110,00	73,00	85,00	790,00	1510,00
4,70	98,00	100,00	69,00	79,00	660,00	1270,00
4,20	92,00	105,00	80,00	88,00	790,00	1550,00
5,20	98,00	114,00	78,00	90,00	670,00	1320,00
5,00	113,00	112,00	83,00	93,00	710,00	1420,00
2,80	71,00	93,00	60,00	71,00	1150,00	1890,00
2,80	71,00	96,00	60,00	72,00	1180,00	1960,00
3,50	90,00	99,00	70,00	80,00	1010,00	1760,00
3,50	94,00	102,00	73,00	83,00	1030,00	1820,00
3,50	80,00	112,00	81,00	91,00	1100,00	1980,00

3,90	77,00	99,00	63,00	75,00	770,00	1440,00
3,40	70,00	95,00	63,00	74,00	880,00	1650,00
3,40	69,00	88,00	60,00	69,00	810,00	1550,00
3,60	72,00	103,00	67,00	79,00	880,00	1670,00
3,50	86,00	102,00	74,00	83,00	940,00	1800,00
3,30	81,00	103,00	74,00	84,00	1100,00	1930,00
4,20	90,00	95,00	66,00	76,00	780,00	1370,00
3,30	81,00	106,00	76,00	86,00	1100,00	1960,00
3,90	101,00	108,00	80,00	89,00	970,00	1740,00
3,30	97,00	105,00	79,00	88,00	1100,00	2030,00
3,10	64,00	110,00	66,00	81,00	1000,00	2000,00
2,90	60,00	105,00	63,00	77,00	990,00	1990,00
3,20	69,00	109,00	69,00	82,00	950,00	1920,00
2,80	65,00	99,00	65,00	76,00	1000,00	2040,00
2,80	69,00	107,00	72,00	84,00	1120,00	2310,00
4,30	75,00	111,00	67,00	82,00	660,00	1440,00
4,50	83,00	113,00	70,00	84,00	640,00	1420,00
4,70	86,00	110,00	72,00	85,00	610,00	1390,00
4,60	84,00	113,00	77,00	89,00	630,00	1460,00
4,50	85,00	116,00	80,00	92,00	670,00	1560,00
3,20	79,00	103,00	65,00	78,00	1040,00	1860,00
3,60	78,00	96,00	66,00	76,00	900,00	1610,00
3,00	76,00	86,00	57,00	67,00	910,00	1660,00
3,10	80,00	93,00	70,00	78,00	1030,00	1900,00
3,50	83,00	97,00	68,00	78,00	900,00	1690,00
3,40	106,00	102,00	65,00	77,00	1150,00	1710,00
3,10	102,00	101,00	66,00	78,00	1300,00	1970,00
3,10	104,00	85,00	59,00	68,00	1060,00	1630,00
3,80	111,00	96,00	76,00	83,00	1060,00	1640,00
2,80	126,00	106,00	77,00	87,00	1500,00	2370,00
2,60	81,00	96,00	59,00	71,00	1310,00	2050,00
2,90	81,00	95,00	61,00	72,00	1170,00	1860,00
2,50	76,00	99,00	65,00	76,00	1480,00	2360,00
2,60	75,00	94,00	66,00	75,00	1340,00	2180,00
2,50	69,00	103,00	71,00	82,00	1510,00	2450,00
2,80	74,00	91,00	59,00	70,00	1060,00	1880,00
4,30	89,00	102,00	65,00	77,00	770,00	1370,00
3,50	92,00	103,00	74,00	84,00	1020,00	1840,00
3,40	86,00	102,00	74,00	83,00	1010,00	1850,00
3,00	72,00	96,00	65,00	75,00	1110,00	1920,00
4,10	89,00	111,00	73,00	86,00	920,00	1620,00
3,20	85,00	106,00	74,00	85,00	1130,00	2020,00
2,80	79,00	110,00	74,00	86,00	1270,00	2310,00
3,20	67,00	96,00	62,00	73,00	960,00	1740,00
4,20	88,00	96,00	68,00	77,00	750,00	1380,00
3,90	93,00	92,00	69,00	77,00	820,00	1510,00
4,30	112,00	94,00	71,00	79,00	750,00	1410,00
4,20	107,00	84,00	59,00	67,00	630,00	1190,00
3,10	72,00	96,00	59,00	71,00	960,00	1700,00
4,10	88,00	92,00	65,00	74,00	730,00	1320,00

3,50	70,00	99,00	64,00	76,00	900,00	1660,00
3,90	102,00	94,00	69,00	77,00	800,00	1510,00
3,90	84,00	90,00	64,00	73,00	750,00	1410,00
3,30	83,00	95,00	58,00	70,00	890,00	1600,00
3,10	90,00	96,00	62,00	73,00	980,00	1760,00
3,80	101,00	92,00	63,00	73,00	810,00	1460,00
3,50	100,00	97,00	69,00	78,00	930,00	1700,00
2,80	92,00	103,00	72,00	82,00	1380,00	2230,00
3,60	109,00	93,00	65,00	74,00	970,00	1590,00
3,20	79,00	97,00	72,00	80,00	1140,00	1900,00
3,00	124,00	104,00	75,00	85,00	1290,00	2180,00
3,50	84,00	108,00	77,00	87,00	980,00	1880,00
3,80	87,00	106,00	71,00	83,00	860,00	1670,00
3,70	101,00	114,00	78,00	90,00	940,00	1840,00
3,60	103,00	104,00	76,00	85,00	900,00	1790,00
3,60	105,00	115,00	80,00	92,00	980,00	1950,00
3,50	77,00	108,00	76,00	87,00	1030,00	1900,00
3,50	72,00	93,00	69,00	77,00	900,00	1670,00
2,90	73,00	103,00	73,00	83,00	1180,00	2200,00
3,70	92,00	99,00	79,00	86,00	940,00	1780,00
3,30	78,00	93,00	56,00	68,00	930,00	1540,00
3,50	78,00	101,00	60,00	74,00	950,00	1610,00
3,30	84,00	92,00	62,00	72,00	970,00	1640,00
3,80	105,00	101,00	72,00	82,00	950,00	1640,00
2,90	81,00	97,00	71,00	80,00	1210,00	2110,00
3,10	71,00	113,00	73,00	86,00	1210,00	2080,00
3,00	69,00	102,00	68,00	79,00	1150,00	2000,00
3,60	91,00	110,00	80,00	90,00	1070,00	1900,00
3,20	79,00	106,00	79,00	88,00	1170,00	2100,00
3,10	98,00	127,00	95,00	106,00	1450,00	2630,00
3,30	84,00	111,00	78,00	89,00	1140,00	2040,00
2,90	78,00	117,00	81,00	93,00	1350,00	2420,00
3,60	89,00	122,00	91,00	101,00	1180,00	2150,00
3,30	65,00	103,00	65,00	78,00	1070,00	1820,00
3,00	60,00	107,00	72,00	84,00	1210,00	2090,00
3,50	83,00	114,00	67,00	83,00	1010,00	1780,00
3,30	71,00	113,00	75,00	88,00	1150,00	2040,00
3,10	68,00	121,00	71,00	88,00	1210,00	2160,00
3,80	88,00	99,00	67,00	78,00	940,00	1560,00
4,30	107,00	94,00	67,00	76,00	790,00	1320,00
4,30	106,00	95,00	65,00	75,00	770,00	1310,00
4,40	130,00	93,00	66,00	75,00	740,00	1290,00

SV_A	TFC_A	ACI_A	LCWI_A	PEP_A	LVET_A	VI_A
101,00	28,00	150,00	5,29	57,00	273,00	101,00
110,00	30,00	164,00	4,52	71,00	302,00	113,00
110,00	27,00	152,00	4,74	72,00	300,00	108,00
107,00	24,00	130,00	5,36	72,00	274,00	99,00
93,00	28,00	105,00	5,03	88,00	251,00	67,00
73,00	23,00	222,00	5,38	52,00	239,00	108,00
65,00	25,00	123,00	5,63	63,00	220,00	85,00
66,00	23,00	151,00	4,64	64,00	211,00	86,00
69,00	26,00	135,00	6,64	60,00	212,00	87,00
67,00	26,00	105,00	5,61	68,00	223,00	78,00
85,00	22,00	121,00	3,55	84,00	273,00	68,00
89,00	23,00	108,00	3,54	84,00	284,00	67,00
77,00	30,00	119,00	3,29	93,00	216,00	68,00
88,00	29,00	120,00	3,89	102,00	282,00	68,00
77,00	32,00	107,00	3,98	107,00	265,00	48,00
76,00	27,00	107,00	4,08	68,00	236,00	79,00
73,00	25,00	103,00	3,58	110,00	273,00	68,00
82,00	27,00	114,00	4,42	89,00	278,00	70,00
90,00	29,00	145,00	4,03	94,00	284,00	90,00
76,00	30,00	105,00	2,54	87,00	236,00	73,00
85,00	25,00	73,00	3,61	58,00	283,00	65,00
93,00	30,00	73,00	4,90	51,00	294,00	63,00
85,00	31,00	63,00	4,46	83,00	270,00	56,00
88,00	35,00	46,00	4,77	90,00	295,00	50,00
65,00	28,00	112,00	3,11	94,00	272,00	58,00
82,00	30,00	136,00	4,02	58,00	254,00	95,00
77,00	32,00	125,00	2,95	79,00	296,00	68,00
89,00	33,00	185,00	4,63	78,00	269,00	120,00
80,00	32,00	124,00	3,86	88,00	290,00	68,00
73,00	27,00	141,00	3,58	98,00	298,00	82,00
75,00	25,00	144,00	4,15	98,00	284,00	96,00
79,00	27,00	155,00	4,84	96,00	278,00	104,00
69,00	27,00	120,00	5,21	91,00	224,00	78,00
78,00	26,00	117,00	4,97	102,00	271,00	81,00
79,00	27,00	91,00	6,88	71,00	206,00	60,00
97,00	28,00	97,00	5,08	81,00	237,00	73,00
103,00	24,00	102,00	5,40	68,00	228,00	76,00
75,00	23,00	66,00	4,00	115,00	224,00	42,00
75,00	25,00	75,00	4,54	113,00	211,00	43,00
80,00	27,00	162,00	3,33	69,00	264,00	102,00
87,00	22,00	151,00	4,68	67,00	280,00	104,00
88,00	25,00	129,00	4,39	71,00	279,00	90,00
76,00	21,00	83,00	4,27	54,00	209,00	70,00
77,00	24,00	82,00	3,72	104,00	259,00	70,00
88,00	28,00	194,00	3,87	76,00	294,00	112,00
97,00	28,00	242,00	4,03	68,00	286,00	151,00
98,00	29,00	218,00	3,35	69,00	296,00	141,00
80,00	25,00	145,00	3,95	106,00	261,00	85,00
78,00	32,00	184,00	4,27	116,00	231,00	95,00

115,00	27,00	140,00	3,63	67,00	314,00	104,00
113,00	24,00	121,00	3,70	58,00	315,00	75,00
124,00	28,00	125,00	4,42	62,00	311,00	107,00
116,00	29,00	112,00	3,69	83,00	313,00	87,00
103,00	25,00	74,00	3,53	105,00	308,00	66,00
77,00	26,00	152,00	3,70	98,00	263,00	92,00
76,00	27,00	389,00	3,13	110,00	227,00	152,00
74,00	27,00	137,00	3,67	111,00	247,00	82,00
77,00	22,00	113,00	3,27	107,00	260,00	69,00
68,00	26,00	127,00	3,43	128,00	227,00	66,00
115,00	29,00	137,00	5,10	63,00	248,00	86,00
96,00	27,00	117,00	4,50	81,00	200,00	74,00
100,00	27,00	109,00	4,22	83,00	199,00	72,00
98,00	22,00	91,00	4,83	84,00	223,00	54,00
75,00	24,00	43,00	4,15	99,00	179,00	41,00
85,00	23,00	200,00	2,72	89,00	304,00	108,00
86,00	32,00	253,00	2,56	70,00	262,00	113,00
80,00	29,00	166,00	2,84	106,00	309,00	91,00
74,00	28,00	145,00	4,30	67,00	262,00	107,00
69,00	25,00	121,00	4,00	74,00	263,00	87,00
69,00	26,00	83,00	4,31	70,00	262,00	80,00
72,00	28,00	161,00	4,48	61,00	225,00	110,00
84,00	32,00	111,00	5,11	58,00	282,00	107,00
61,00	28,00	128,00	2,88	84,00	280,00	73,00
56,00	31,00	117,00	2,20	81,00	222,00	75,00
61,00	25,00	113,00	3,03	73,00	230,00	69,00
59,00	30,00	87,00	3,27	88,00	240,00	58,00
91,00	24,00	147,00	4,46	74,00	275,00	90,00
87,00	24,00	129,00	3,54	84,00	294,00	67,00
84,00	21,00	96,00	4,04	84,00	256,00	65,00
93,00	23,00	115,00	4,69	69,00	264,00	73,00
68,00	22,00	71,00	4,11	94,00	184,00	58,00
94,00	27,00	132,00	5,30	68,00	248,00	81,00
97,00	26,00	93,00	4,27	54,00	243,00	75,00
89,00	24,00	75,00	5,34	73,00	252,00	62,00
87,00	23,00	63,00	5,93	37,00	276,00	43,00
86,00	28,00	77,00	5,83	55,00	215,00	59,00
79,00	27,00	161,00	3,57	103,00	233,00	109,00
75,00	23,00	91,00		118,00	254,00	75,00
85,00	27,00	143,00	4,43	111,00	277,00	86,00
87,00	36,00	113,00	5,08	89,00	249,00	92,00
75,00	24,00	92,00	4,47	113,00	241,00	59,00
89,00	26,00	165,00	4,28	77,00	277,00	117,00
91,00	27,00	151,00	4,47	69,00	261,00	112,00
89,00	27,00	138,00	3,99	101,00	275,00	102,00
91,00	26,00	113,00	4,96	64,00	254,00	95,00
65,00	21,00	75,00	3,04	135,00	257,00	43,00
76,00	30,00	139,00	2,72	68,00	310,00	86,00
68,00	25,00	113,00	3,46	79,00	252,00	88,00
65,00	24,00	107,00	3,60	83,00	230,00	79,00

56,00	26,00	129,00	3,96	108,00	192,00	78,00
92,00	28,00	232,00	3,84	65,00	272,00	123,00
90,00	24,00	182,00	3,52	69,00	295,00	100,00
93,00	29,00	225,00	3,50	74,00	287,00	117,00
93,00	27,00	225,00	4,61	72,00	258,00	117,00
95,00	30,00	198,00	4,21	92,00	284,00	102,00
70,00	27,00	204,00	4,66	58,00	261,00	114,00
74,00	27,00	198,00	3,28	77,00	283,00	117,00
72,00	27,00	216,00	3,88	81,00	256,00	119,00
75,00	28,00	194,00	4,31	84,00	236,00	114,00
108,00	23,00	104,00	4,30	74,00	271,00	71,00
103,00	22,00	84,00	3,90	68,00	290,00	54,00
120,00	26,00	114,00	4,46	58,00	304,00	70,00
112,00	26,00	99,00	5,03	71,00	293,00	56,00
67,00	28,00	131,00	3,77	67,00	270,00	81,00
73,00	23,00	133,00	2,67	78,00	346,00	69,00
72,00	27,00	121,00	3,44	87,00	299,00	79,00
74,00	27,00	115,00	3,53	76,00	291,00	79,00
59,00	24,00	105,00	3,29	116,00	258,00	54,00
98,00	26,00	123,00	4,02	86,00	291,00	76,00
77,00	24,00	63,00	3,96	57,00	212,00	53,00
86,00	26,00	83,00	4,46	43,00	198,00	62,00
84,00	27,00	76,00	4,00	122,00	245,00	62,00
124,00	22,00	88,00	4,52	75,00	261,00	54,00
132,00	22,00	93,00	4,86	75,00	276,00	55,00
138,00	22,00	86,00	4,27	71,00	283,00	59,00
109,00	22,00	69,00	3,65	86,00	249,00	43,00
91,00	26,00	85,00		97,00	268,00	58,00
110,00	33,00	86,00	4,32	49,00	241,00	79,00
99,00	42,00	181,00	4,18	97,00	227,00	78,00
91,00	33,00	63,00	4,05	106,00	217,00	60,00
96,00	21,00	120,00	5,23	76,00	271,00	75,00
94,00	21,00	114,00	4,88	86,00	265,00	72,00
104,00	29,00	137,00	5,29	97,00	279,00	90,00
96,00	26,00	112,00	4,60	113,00	265,00	69,00
79,00	26,00	92,00	3,38	81,00	206,00	59,00
98,00	28,00	121,00	5,84	80,00	239,00	76,00
102,00	27,00	127,00	5,60	86,00	256,00	78,00
89,00	28,00	87,00	3,97	97,00	238,00	56,00
121,00	29,00	192,00	5,04	61,00	323,00	125,00
106,00	25,00	154,00	3,73	91,00	295,00	96,00
100,00	25,00	138,00	3,78	96,00	274,00	86,00
93,00	27,00	116,00	3,95	107,00	250,00	70,00
81,00	25,00	104,00	4,16	69,00	234,00	74,00
88,00	26,00	89,00	4,82	87,00	266,00	70,00
81,00	27,00	82,00	4,69	114,00	245,00	60,00
88,00	34,00	73,00	4,37	94,00	257,00	57,00
90,00	23,00	75,00	4,73	102,00	247,00	55,00
90,00	27,00	94,00	3,99	61,00	294,00	69,00
87,00	34,00	87,00	3,77	69,00	279,00	65,00

88,00	33,00	80,00	3,50	71,00	278,00	63,00
95,00	34,00	96,00	3,98	57,00	295,00	66,00
81,00	31,00	170,00	2,74	70,00	265,00	93,00
71,00	25,00	114,00	2,93	110,00	253,00	76,00
85,00	35,00	149,00	2,96	89,00	284,00	93,00
98,00	32,00	119,00	4,41	62,00	297,00	103,00
86,00	30,00	117,00	3,54	73,00	273,00	86,00
80,00	31,00	131,00	3,18	87,00	250,00	78,00
79,00	35,00	148,00	4,68	69,00	223,00	77,00
61,00	35,00	89,00	3,89	92,00	176,00	67,00
71,00	36,00	96,00	4,19	104,00	222,00	61,00
72,00	27,00	97,00	3,72	67,00	250,00	74,00
79,00	32,00	96,00	4,11	49,00	243,00	77,00
82,00	33,00	111,00	4,84	42,00	234,00	73,00
79,00	32,00	88,00	4,69	41,00	219,00	64,00
65,00	31,00	54,00	3,58	99,00	231,00	47,00
96,00	27,00	119,00	3,77	62,00	287,00	79,00
93,00	32,00	189,00	3,65	69,00	226,00	110,00
91,00	38,00	168,00	3,34	62,00	220,00	90,00
82,00	29,00	149,00	4,19	88,00	219,00	80,00
87,00	26,00	76,00	3,54	76,00	278,00	59,00
77,00	24,00	113,00	4,01	98,00	271,00	62,00
80,00	26,00	125,00	4,34	102,00	258,00	71,00
77,00	25,00	115,00	4,33	109,00	246,00	65,00
81,00	22,00	77,00	5,37	75,00	246,00	60,00
75,00	17,00	87,00	2,65	93,00	204,00	56,00
104,00	22,00	114,00	4,18	71,00	282,00	74,00
98,00	26,00	112,00	3,72	88,00	249,00	81,00
101,00	28,00	71,00	6,90	46,00	260,00	61,00
102,00	31,00	65,00	6,51	54,00	258,00	58,00
100,00	29,00	143,00	4,11	56,00	222,00	92,00
97,00	31,00	142,00	3,37	70,00	202,00	98,00
100,00	31,00	113,00	3,98	71,00	223,00	81,00
112,00	29,00	166,00	5,62	60,00	218,00	103,00
99,00	31,00	99,00	3,86	76,00	212,00	74,00
73,00	30,00	159,00	3,71	75,00	245,00	96,00
66,00	29,00	156,00	2,34	94,00	237,00	82,00
71,00	39,00	162,00	2,76	88,00	246,00	83,00
70,00	29,00	128,00	3,46	93,00	246,00	81,00
64,00	23,00	104,00	3,11	108,00	248,00	60,00
85,00	30,00	110,00	3,14	61,00	269,00	82,00
73,00	27,00	91,00	3,24	89,00	261,00	61,00
63,00	21,00	150,00	2,45	80,00	223,00	50,00
78,00	28,00	95,00	3,57	90,00	248,00	69,00
94,00	31,00	121,00	3,85	63,00	269,00	76,00
82,00	31,00	90,00	4,84	74,00	212,00	70,00
80,00	30,00	66,00	3,34	69,00	229,00	56,00
72,00	31,00	82,00	3,52	86,00	218,00	51,00
84,00	29,00	109,00	3,78	62,00	237,00	76,00
83,00	28,00	178,00	4,08	86,00	201,00	95,00

82,00	28,00	154,00	4,28	101,00	236,00	75,00
97,00	34,00	132,00	4,07	65,00	273,00	99,00
96,00	34,00	150,00	3,59	57,00	216,00	107,00
94,00	32,00	94,00	4,93	49,00	252,00	68,00
111,00	34,00	85,00	4,92	48,00	285,00	83,00
103,00	26,00	142,00	3,73	80,00	289,00	93,00
103,00	26,00	123,00	3,47	89,00	297,00	88,00
87,00	26,00	80,00	4,86	83,00	241,00	61,00
82,00	27,00	66,00	4,90	73,00	209,00	55,00
82,00	27,00	150,00	4,53	76,00	261,00	98,00
77,00	27,00	138,00	3,68	74,00	223,00	103,00
97,00	26,00	132,00	4,74	64,00	281,00	108,00
80,00	26,00	130,00	4,15	89,00	209,00	86,00
92,00	25,00	86,00	5,55	60,00	257,00	74,00
95,00	30,00	187,00	4,48	96,00	280,00	121,00
95,00	35,00	103,00	5,22	81,00	272,00	92,00
93,00	30,00	152,00	4,42	94,00	256,00	101,00
81,00	32,00	73,00	4,97	94,00	218,00	71,00
56,00	31,00	215,00	3,59	91,00	243,00	107,00
57,00	26,00	184,00	4,17	90,00	233,00	93,00
59,00	25,00	179,00	4,14	95,00	227,00	87,00
59,00	33,00	174,00	4,52	100,00	223,00	78,00
93,00	29,00	161,00	3,60	71,00	286,00	91,00
92,00	30,00	169,00	3,70	76,00	256,00	105,00
93,00	29,00	125,00	4,52	68,00	246,00	91,00
104,00	29,00	110,00	3,98	60,00	275,00	90,00
70,00	30,00	225,00	2,79	53,00	241,00	112,00
80,00	29,00	197,00	2,98	63,00	294,00	152,00
83,00	30,00	248,00	3,61	55,00	272,00	159,00
72,00	28,00	145,00	4,06	75,00	247,00	106,00
75,00	32,00	202,00	3,09	95,00	261,00	121,00
56,00	32,00	119,00	2,65	96,00	200,00	73,00
47,00	31,00	107,00	3,42	115,00	163,00	61,00
48,00	33,00	80,00	3,28	115,00	180,00	50,00
51,00	34,00	88,00	3,98	103,00	153,00	70,00
60,00	30,00	220,00	3,70	71,00	239,00	121,00
67,00	27,00	213,00	4,22	70,00	244,00	119,00
58,00	30,00	122,00	4,18	84,00	228,00	72,00
60,00	29,00	133,00	4,63	80,00	216,00	67,00
86,00	30,00	244,00	3,66	68,00	294,00	127,00
65,00	33,00	185,00	2,48	88,00	195,00	94,00
76,00	32,00	144,00	2,82	104,00	294,00	77,00
77,00	33,00	167,00	3,40	104,00	261,00	94,00
68,00	33,00	156,00	3,84	109,00	243,00	74,00
66,00	32,00	170,00	3,82	126,00	228,00	75,00
67,00	32,00	163,00	3,85	136,00	236,00	73,00
77,00	32,00	176,00	3,59	114,00	255,00	83,00
75,00	31,00	132,00	5,28	54,00	232,00	74,00
66,00	25,00	99,00	4,19	73,00	226,00	59,00
71,00	35,00	87,00	6,13	73,00	235,00	60,00

66,00	34,00	84,00	5,32	92,00	220,00	55,00
72,00	31,00	107,00	5,54	43,00	240,00	73,00
67,00	32,00	90,00	5,93	52,00	231,00	64,00
72,00	36,00	123,00	7,58	29,00	180,00	77,00
53,00	33,00	57,00	4,37	88,00	192,00	43,00
78,00	29,00	254,00	3,59	70,00	242,00	146,00
80,00	31,00	217,00	4,39	77,00	261,00	131,00
73,00	31,00	224,00	5,26	104,00	232,00	122,00
80,00	29,00	197,00	4,45	87,00	243,00	120,00
78,00	26,00	164,00	4,28	94,00	248,00	98,00
64,00	27,00	66,00	3,28	75,00	233,00	43,00
62,00	28,00	67,00	3,17	64,00	199,00	44,00
43,00	28,00	42,00	2,66	96,00	206,00	22,00
104,00	32,00	109,00	4,18	52,00	257,00	88,00
109,00	31,00	204,00	4,56	59,00	243,00	113,00
116,00	30,00	142,00	6,61	57,00	266,00	89,00
118,00	30,00	144,00	6,93	53,00	255,00	90,00
74,00	26,00	190,00	3,87	77,00	274,00	112,00
62,00	27,00	159,00	3,28	81,00	203,00	95,00
62,00	29,00	151,00	3,91	104,00	258,00	77,00
66,00	39,00	150,00	3,54	104,00	260,00	85,00
94,00	28,00	165,00	3,44	50,00	260,00	105,00
99,00	29,00	182,00	5,80	49,00	249,00	115,00
107,00	29,00	177,00	3,96	51,00	282,00	112,00
103,00	31,00	157,00	3,97	51,00	251,00	106,00
106,00	30,00	145,00	4,34	52,00	259,00	101,00
68,00	28,00	183,00	2,36	88,00	308,00	100,00
63,00	27,00	132,00	2,50	106,00	267,00	91,00
69,00	24,00	141,00	2,50	86,00	314,00	82,00
63,00	28,00	152,00	2,78	112,00	258,00	95,00
85,00	31,00	93,00	4,39	89,00	297,00	80,00
72,00	29,00	82,00	3,28	83,00	217,00	70,00
85,00	33,00	103,00	3,20	86,00	264,00	85,00
92,00	30,00	63,00	4,51	62,00	334,00	69,00
84,00	21,00	142,00	3,41	90,00	298,00	79,00
91,00	27,00	192,00	3,58	93,00	282,00	117,00
79,00	23,00	130,00	3,26	101,00	255,00	80,00
85,00	24,00	154,00	4,41	102,00	252,00	89,00
89,00	27,00	170,00	4,83	104,00	253,00	98,00
86,00	28,00	151,00	3,32	87,00	231,00	90,00
89,00	30,00	155,00	3,09	107,00	253,00	86,00
93,00	29,00	152,00	3,69	111,00	278,00	87,00
98,00	30,00	153,00	3,94	97,00	281,00	89,00
91,00	29,00	156,00	3,90	111,00	262,00	85,00
83,00	30,00	129,00	3,61	74,00	279,00	86,00
71,00	29,00	101,00	3,39	89,00	237,00	65,00
72,00	32,00	95,00	2,78	91,00	223,00	65,00
77,00	31,00	94,00	2,88	93,00	250,00	59,00
127,00	28,00	155,00	4,79	62,00	270,00	95,00
131,00	27,00	132,00	5,99	63,00	263,00	96,00

131,00	25,00	129,00	6,62	68,00	263,00	91,00
131,00	29,00	106,00	5,87	75,00	259,00	83,00
110,00	28,00	102,00	5,50	107,00	223,00	63,00
96,00	33,00	220,00	3,72	76,00	302,00	128,00
103,00	31,00	245,00	4,30	86,00	291,00	145,00
107,00	35,00	251,00	4,54	64,00	293,00	159,00
98,00	38,00	142,00	4,23	59,00	284,00	93,00
95,00	33,00	174,00	5,06	89,00	242,00	119,00
82,00	27,00	188,00	3,81	79,00	292,00	107,00
78,00	26,00	163,00	3,53	106,00	284,00	97,00
88,00	28,00	133,00	3,99	64,00	282,00	97,00
75,00	27,00	102,00	4,07	120,00	253,00	82,00
77,00	29,00	101,00	3,45	118,00	293,00	66,00
66,00	31,00	151,00	4,00	68,00	259,00	95,00
66,00	33,00	127,00	3,17	78,00	282,00	74,00
58,00	36,00	121,00	3,55	80,00	213,00	76,00
68,00	35,00	132,00	2,81	86,00	292,00	70,00
72,00	37,00	126,00	3,13	86,00	292,00	73,00
84,00	29,00	205,00	3,12	74,00	292,00	139,00
88,00	30,00	207,00	4,10	65,00	262,00	144,00
84,00	31,00	159,00	3,35	88,00	271,00	131,00
90,00	32,00	154,00	4,26	76,00	281,00	122,00
93,00	34,00	178,00	6,34	59,00	245,00	98,00
84,00	31,00	182,00	5,82	80,00	223,00	84,00
93,00	31,00	123,00	4,42	66,00	268,00	85,00
82,00	34,00	145,00	5,72	72,00	219,00	79,00
80,00	35,00	110,00	4,95	102,00	240,00	68,00
85,00	27,00	163,00	3,73	72,00	307,00	103,00
86,00	29,00	169,00	3,91	69,00	283,00	108,00
88,00	31,00	117,00	4,37	68,00	284,00	99,00
87,00	28,00	134,00	3,72	78,00	293,00	88,00
77,00	30,00	149,00	3,78	110,00	241,00	92,00
71,00	33,00	168,00	3,58	100,00	272,00	90,00
70,00	35,00	140,00	3,88	84,00	227,00	90,00
62,00	34,00	154,00	3,99	108,00	233,00	69,00
80,00	27,00	172,00	3,81	101,00	275,00	105,00
83,00	25,00	182,00	4,00	91,00	262,00	112,00
89,00	27,00	168,00	4,47	74,00	259,00	111,00
84,00	26,00	171,00	3,98	105,00	258,00	103,00
84,00	26,00	152,00	3,79	112,00	266,00	91,00
104,00	36,00	165,00	4,76	59,00	275,00	101,00
100,00	33,00	148,00	4,83	68,00	261,00	97,00
94,00	35,00	104,00	5,01	83,00	239,00	83,00
108,00	35,00	134,00	6,13	53,00	253,00	97,00
90,00	38,00	131,00	6,12	96,00	216,00	80,00
70,00	29,00	189,00	2,57	97,00	275,00	106,00
64,00	27,00	156,00	2,55	90,00	233,00	92,00
63,00	26,00	127,00	3,59	80,00	193,00	79,00
64,00	33,00	99,00	3,74	73,00	200,00	67,00
79,00	34,00	138,00	4,20	109,00	278,00	79,00

97,00	31,00	179,00	3,80	90,00	274,00	108,00
87,00	31,00	173,00	3,22	87,00	218,00	104,00
94,00	33,00	140,00	2,95	97,00	295,00	81,00
90,00	33,00	168,00	3,67	96,00	237,00	103,00
81,00	33,00	145,00	3,79	109,00	216,00	88,00
74,00	36,00	128,00	3,63	99,00	270,00	79,00
84,00	32,00	96,00	4,10	61,00	295,00	82,00
74,00	33,00	82,00	3,75	95,00	268,00	69,00
71,00	34,00	81,00	4,55	93,00	250,00	63,00
65,00	32,00	88,00	3,82	109,00	239,00	53,00
97,00	35,00	116,00	3,24	80,00	241,00	81,00
99,00	33,00	134,00	2,92	93,00	246,00	84,00
93,00	35,00	116,00	3,46	105,00	241,00	71,00
91,00	36,00	115,00	2,76	108,00	245,00	66,00
87,00	35,00	89,00	3,03	115,00	239,00	56,00
128,00	29,00	117,00	4,61	100,00	303,00	77,00
125,00	30,00	95,00	4,92	105,00	281,00	69,00
126,00	30,00	54,00	5,16	95,00	276,00	60,00
126,00	34,00	84,00	5,44	101,00	278,00	58,00
126,00	32,00	64,00	5,47	96,00	310,00	47,00
76,00	31,00	154,00	3,20	78,00	245,00	79,00
77,00	26,00	131,00	3,50	90,00	271,00	73,00
77,00	33,00	125,00	2,58	116,00	287,00	67,00
74,00	36,00	108,00	3,14	121,00	271,00	63,00
83,00	35,00	137,00	3,53	108,00	272,00	76,00
52,00	32,00	236,00	3,40	62,00	192,00	120,00
47,00	33,00	148,00	3,03	71,00	181,00	88,00
47,00	32,00	144,00	2,72	70,00	178,00	86,00
55,00	33,00	144,00	4,17	81,00	240,00	83,00
39,00	33,00	116,00	3,19	108,00	154,00	67,00
52,00	31,00	161,00	2,37	74,00	196,00	86,00
59,00	32,00	147,00	2,69	70,00	211,00	94,00
55,00	31,00	143,00	2,39	107,00	263,00	68,00
55,00	34,00	150,00	2,52	99,00	213,00	81,00
60,00	34,00	160,00	2,72	114,00	264,00	78,00
68,00	29,00	135,00	2,51	89,00	205,00	84,00
87,00	31,00	162,00	4,23	59,00	229,00	109,00
69,00	32,00	118,00	3,81	77,00	189,00	81,00
73,00	34,00	93,00	3,70	100,00	232,00	70,00
71,00	32,00	173,00	2,85	94,00	237,00	90,00
77,00	31,00	161,00	4,54	81,00	260,00	84,00
73,00	29,00	144,00	3,55	91,00	231,00	78,00
70,00	34,00	96,00	3,18	101,00	240,00	63,00
83,00	28,00	173,00	2,97	92,00	259,00	96,00
89,00	29,00	186,00	4,21	92,00	269,00	103,00
79,00	29,00	171,00	3,85	96,00	236,00	85,00
74,00	30,00	164,00	4,37	104,00	209,00	76,00
74,00	31,00	165,00	3,60	118,00	206,00	85,00
79,00	31,00	154,00	2,85	89,00	254,00	96,00
89,00	38,00	156,00	4,02	60,00	235,00	108,00

93,00	36,00	134,00	3,39	79,00	282,00	97,00
77,00	34,00	80,00	3,85	107,00	262,00	61,00
88,00	34,00	146,00	3,66	102,00	261,00	89,00
69,00	28,00	90,00	2,95	56,00	190,00	71,00
63,00	29,00	83,00	2,93	85,00	189,00	66,00
67,00	30,00	104,00	3,54	64,00	190,00	67,00
65,00	29,00	65,00	3,51	76,00	212,00	52,00
51,00	29,00	137,00	2,98	76,00	197,00	69,00
54,00	30,00	162,00	3,35	90,00	194,00	83,00
72,00	32,00	149,00	3,32	66,00	258,00	92,00
43,00	32,00	104,00	3,29	109,00	183,00	46,00
81,00	26,00	61,00	4,03	110,00	258,00	65,00
84,00	29,00	79,00	4,10	111,00	266,00	64,00
74,00	27,00	56,00	4,43	82,00	257,00	41,00
70,00	28,00	45,00	4,01	68,00	227,00	38,00
73,00	28,00	49,00	4,37	84,00	228,00	41,00
83,00	35,00	137,00	3,98	78,00	257,00	74,00
87,00	32,00	145,00	3,48	85,00	281,00	79,00
76,00	32,00	125,00	3,10	99,00	238,00	69,00
81,00	35,00	106,00	4,14	105,00	252,00	68,00
71,00	37,00	254,00	2,87	83,00	231,00	123,00
77,00	35,00	160,00	3,31	66,00	266,00	105,00
68,00	35,00	148,00	3,06	83,00	217,00	97,00
66,00	36,00	135,00	4,06	98,00	228,00	78,00
65,00	33,00	116,00	2,99	105,00	227,00	75,00
77,00	28,00	127,00	3,53	104,00	306,00	87,00
77,00	30,00	118,00	3,06	108,00	312,00	79,00
72,00	27,00	112,00	4,28	106,00	252,00	74,00
75,00	29,00	134,00	3,69	130,00	264,00	80,00
57,00	28,00	112,00	4,38	139,00	218,00	50,00
72,00	31,00	131,00	3,89	53,00	194,00	83,00
69,00	28,00	85,00	3,60	79,00	223,00	70,00
75,00	31,00	68,00	4,84	87,00	241,00	70,00
89,00	33,00	233,00	3,29	61,00	311,00	130,00
86,00	34,00	183,00	3,34	62,00	297,00	106,00
81,00	31,00	151,00	3,84	76,00	248,00	103,00
89,00	35,00	177,00	3,80	75,00	290,00	104,00
90,00	33,00	167,00	3,59	73,00	307,00	96,00
72,00	33,00	160,00	3,83	79,00	273,00	100,00
68,00	33,00	157,00	4,26	91,00	233,00	103,00
72,00	34,00	127,00	4,17	98,00	254,00	91,00
61,00	32,00	85,00	4,26	86,00	195,00	76,00

STR_A	CO_B	CI_B	HR_B	BPS_B	BPD_B	MAP_B
21,00	7,00	3,80	79,00	109,00	66,00	80,00
24,00	7,30	3,80	68,00	113,00	73,00	86,00
24,00	7,20	3,70	72,00	111,00	71,00	84,00
26,00	7,00	3,60	76,00	109,00	76,00	87,00
36,00	8,40	4,20	74,00	118,00	84,00	95,00
22,00	7,40	4,50	109,00	114,00	72,00	86,00
29,00	5,80	3,50	115,00	112,00	74,00	87,00
30,00	6,50	3,90	104,00	109,00	70,00	83,00
29,00	7,30	4,30	108,00	118,00	83,00	95,00
35,00	6,30	3,70	113,00	107,00	77,00	87,00
31,00	6,70	3,60	80,00	104,00	62,00	76,00
29,00	6,50	3,40	87,00	109,00	67,00	81,00
44,00	6,70	3,50	80,00	95,00	67,00	76,00
36,00	5,70	3,00	70,00	116,00	75,00	89,00
40,00	6,40	3,30	76,00	120,00	83,00	95,00
29,00	6,00	3,30	83,00	104,00	72,00	83,00
40,00	6,00	3,40	85,00	106,00	66,00	79,00
32,00	6,00	3,30	88,00	103,00	72,00	82,00
33,00	6,80	3,70	77,00	103,00	73,00	83,00
37,00	5,40	3,00	67,00	86,00	58,00	67,00
21,00	6,70	3,60	79,00	100,00	65,00	77,00
17,00	7,60	4,00	82,00	110,00	74,00	86,00
32,00	7,70	4,00	100,00	107,00	80,00	89,00
32,00	8,80	4,40	96,00	116,00	82,00	93,00
35,00	4,40	2,60	73,00	98,00	70,00	79,00
23,00	5,00	2,80	72,00	99,00	68,00	78,00
27,00	6,10	3,40	72,00	93,00	64,00	74,00
32,00	5,60	3,10	77,00	103,00	74,00	84,00
31,00	4,90	2,70	68,00	118,00	87,00	97,00
33,00	5,10	3,00	85,00	106,00	74,00	85,00
35,00	5,80	3,40	84,00	107,00	70,00	82,00
35,00	7,20	4,20	88,00	112,00	75,00	87,00
43,00	7,50	4,20	101,00	106,00	73,00	84,00
38,00	5,90	3,30	87,00	110,00	77,00	88,00
36,00	8,80	4,50	113,00	130,00	83,00	99,00
35,00	9,20	4,60	95,00	120,00	81,00	94,00
30,00	9,00	4,40	94,00	108,00	77,00	87,00
53,00	8,30	4,00	93,00	108,00	70,00	83,00
55,00	8,90	4,20	97,00	118,00	77,00	91,00
27,00	5,30	3,10	72,00	95,00	60,00	72,00
24,00	6,90	3,90	83,00	99,00	65,00	76,00
26,00	6,70	3,70	84,00	100,00	65,00	77,00
26,00	6,80	3,70	95,00	96,00	64,00	75,00
40,00	6,70	3,60	78,00	107,00	67,00	80,00
26,00	6,70	3,80	76,00	101,00	65,00	77,00
24,00	7,00	3,90	82,00	93,00	63,00	73,00
24,00	6,50	3,60	68,00	99,00	63,00	75,00
41,00	5,90	3,20	76,00	108,00	75,00	86,00
50,00	7,00	3,80	77,00	106,00	76,00	86,00

22,00	7,00	3,60	60,00	105,00	62,00	76,00
19,00	6,70	3,40	65,00	98,00	59,00	72,00
20,00	8,40	4,20	66,00	99,00	64,00	76,00
27,00	6,70	3,30	60,00	102,00	66,00	78,00
34,00	6,20	3,00	60,00	102,00	72,00	82,00
37,00	6,50	3,70	79,00	91,00	57,00	68,00
49,00	6,10	3,40	73,00	93,00	57,00	69,00
45,00	6,00	3,30	89,00	96,00	63,00	74,00
41,00	5,80	3,20	85,00	87,00	57,00	67,00
57,00	6,10	3,30	88,00	91,00	59,00	70,00
26,00	9,80	4,70	77,00	109,00	68,00	82,00
42,00	8,40	4,00	69,00	115,00	64,00	81,00
42,00	9,20	4,30	81,00	109,00	65,00	80,00
38,00	9,60	4,40	89,00	108,00	70,00	83,00
57,00	9,00	4,10	86,00	102,00	67,00	79,00
29,00	5,10	2,90	65,00	96,00	56,00	69,00
28,00	5,60	3,20	63,00	100,00	57,00	71,00
34,00	4,30	2,50	58,00	114,00	74,00	87,00
26,00	5,80	3,50	79,00	102,00	71,00	81,00
29,00	5,50	3,30	92,00	93,00	63,00	73,00
27,00	5,70	3,40	94,00	100,00	73,00	82,00
28,00	5,50	3,30	81,00	101,00	73,00	82,00
21,00	6,10	3,50	80,00	111,00	70,00	84,00
30,00	4,70	2,90	79,00	96,00	66,00	76,00
37,00	4,70	2,90	73,00	94,00	64,00	74,00
32,00	5,30	3,20	82,00	96,00	61,00	73,00
38,00	5,00	3,00	87,00	94,00	62,00	73,00
27,00	7,40	4,10	80,00	105,00	66,00	79,00
29,00	6,50	3,50	73,00	100,00	67,00	78,00
33,00	7,20	3,80	82,00	98,00	61,00	73,00
27,00	7,10	3,70	87,00	106,00	72,00	83,00
52,00	7,80	4,00	83,00	107,00	70,00	82,00
28,00	8,50	4,40	102,00	107,00	73,00	84,00
23,00	7,60	3,90	84,00	103,00	70,00	81,00
31,00	6,30	3,20	114,00	103,00	71,00	82,00
14,00	8,40	4,30	105,00	104,00	69,00	81,00
26,00	7,70	3,90	91,00	111,00	76,00	88,00
45,00	7,30	4,00	80,00	100,00	62,00	75,00
48,00	6,60	3,60	85,00			
40,00	7,20	3,90	82,00	105,00	71,00	82,00
36,00	6,60	3,60	101,00	98,00	67,00	77,00
47,00	7,40	3,90	96,00	102,00	73,00	83,00
28,00	6,20	3,50	71,00	99,00	60,00	73,00
27,00	6,60	3,60	80,00	104,00	64,00	77,00
37,00	6,40	3,50	82,00	96,00	64,00	75,00
26,00	7,00	3,80	96,00	102,00	68,00	79,00
53,00	5,40	2,80	77,00	97,00	69,00	78,00
22,00	5,00	3,00	68,00	90,00	56,00	67,00
31,00	5,50	3,30	83,00	94,00	54,00	67,00
36,00	5,80	3,40	83,00	90,00	62,00	71,00

57,00	5,90	3,50	109,00	93,00	65,00	74,00
24,00	6,60	3,70	72,00	95,00	58,00	70,00
23,00	6,20	3,50	75,00	100,00	57,00	71,00
26,00	6,00	3,40	63,00	106,00	64,00	78,00
28,00	7,20	3,90	80,00	116,00	71,00	86,00
32,00	6,00	3,20	68,00	117,00	75,00	89,00
22,00	6,50	4,10	100,00	90,00	59,00	69,00
27,00	5,90	3,60	81,00	85,00	54,00	64,00
32,00	5,70	3,50	83,00	84,00	56,00	65,00
36,00	6,70	3,90	105,00	88,00	60,00	69,00
28,00	8,50	4,10	80,00	108,00	63,00	78,00
24,00	7,50	3,60	85,00	102,00	61,00	75,00
19,00	7,90	3,80	72,00	109,00	63,00	78,00
25,00	8,30	3,90	77,00	124,00	74,00	91,00
26,00	4,80	3,00	66,00	104,00	68,00	80,00
23,00	4,50	2,70	58,00	110,00	69,00	83,00
29,00	4,90	2,90	65,00	111,00	64,00	80,00
27,00	4,80	2,80	69,00	114,00	75,00	88,00
46,00	4,00	2,30	71,00	118,00	76,00	90,00
31,00	7,70	3,90	80,00	108,00	74,00	85,00
27,00	7,40	3,80	87,00	100,00	69,00	79,00
22,00	9,10	4,50	96,00	102,00	70,00	81,00
50,00	6,60	3,30	92,00	100,00	73,00	82,00
29,00	10,20	4,40	77,00	108,00	62,00	77,00
27,00	10,40	4,50	73,00	102,00	62,00	75,00
26,00	9,80	4,20	71,00	102,00	64,00	77,00
35,00	9,10	3,90	83,00	103,00	64,00	77,00
36,00	8,30	4,10	86,00			
21,00	7,80	3,80	79,00	98,00	66,00	77,00
45,00	8,10	3,90	77,00	97,00	69,00	78,00
49,00	8,20	3,90	104,00	99,00	69,00	79,00
28,00	8,30	4,30	85,00	106,00	64,00	78,00
33,00	8,20	4,20	85,00	114,00	73,00	87,00
35,00	8,30	4,20	88,00	113,00	70,00	84,00
43,00	7,90	3,90	80,00	111,00	74,00	86,00
40,00	6,50	3,30	81,00	100,00	64,00	76,00
34,00	8,20	4,10	94,00	131,00	86,00	101,00
34,00	7,90	3,90	86,00	121,00	74,00	90,00
42,00	7,80	3,80	78,00	117,00	73,00	88,00
19,00	7,50	3,90	68,00	112,00	66,00	81,00
31,00	7,30	3,70	67,00	111,00	68,00	82,00
35,00	7,00	3,60	68,00	109,00	69,00	82,00
43,00	6,80	3,30	68,00	112,00	72,00	85,00
30,00	6,60	3,50	78,00	105,00	66,00	79,00
33,00	6,60	3,50	85,00	105,00	73,00	84,00
47,00	6,90	3,50	89,00	115,00	75,00	88,00
37,00	7,10	3,50	84,00	109,00	79,00	89,00
41,00	6,50	3,20	94,00	103,00	72,00	82,00
22,00	5,80	3,10	69,00	97,00	67,00	77,00
25,00	5,90	3,10	75,00	104,00	70,00	81,00

26,00	6,00	3,20	71,00	100,00	68,00	79,00
19,00	6,40	3,30	72,00	103,00	68,00	80,00
28,00	5,90	3,30	66,00	101,00	54,00	70,00
44,00	5,20	2,90	61,00	95,00	56,00	69,00
32,00	5,20	2,90	66,00	98,00	63,00	75,00
21,00	6,70	3,70	109,00	99,00	67,00	78,00
27,00	6,20	3,40	70,00	100,00	66,00	77,00
35,00						
31,00	7,10	3,80	103,00	103,00	66,00	78,00
56,00	6,80	3,70	119,00	93,00	66,00	75,00
47,00	7,00	3,70	107,00	101,00	69,00	80,00
27,00	6,40	3,70	93,00	92,00	61,00	71,00
20,00	7,70	4,30	95,00	93,00	64,00	74,00
18,00	8,80	4,90	97,00	104,00	74,00	84,00
19,00	8,00	4,30	109,00	98,00	66,00	77,00
46,00	5,70	3,10	86,00	102,00	76,00	85,00
22,00	7,10	3,80	79,00	97,00	59,00	72,00
31,00	5,90	3,10	79,00	98,00	64,00	75,00
28,00	6,10	3,20	75,00	101,00	60,00	74,00
41,00	7,80	4,10	91,00	94,00	61,00	72,00
28,00	5,10	2,60	68,00	93,00	63,00	73,00
36,00	7,20	3,90	84,00	99,00	71,00	80,00
40,00	7,40	4,00	89,00	100,00	70,00	80,00
45,00	7,20	3,80	90,00	109,00	69,00	82,00
31,00	8,00	4,20	103,00	107,00	69,00	82,00
46,00	6,50	3,30	74,00	91,00	60,00	70,00
25,00	7,70	3,90	81,00	100,00	63,00	75,00
37,00	7,20	3,60	73,00	98,00	66,00	77,00
18,00	8,40	4,20	111,00	100,00	72,00	81,00
21,00	6,80	3,30	103,00	111,00	77,00	88,00
27,00	8,90	4,50	84,00	95,00	62,00	73,00
35,00	8,50	4,30	76,00	96,00	62,00	73,00
32,00	7,40	3,70	79,00	100,00	65,00	77,00
28,00	8,70	4,30	96,00	100,00	66,00	77,00
36,00	8,50	4,10	81,00	104,00	68,00	80,00
31,00			85,00	93,00	60,00	71,00
40,00	5,00	2,90	72,00	90,00	55,00	67,00
36,00	6,20	3,60	81,00	89,00	60,00	70,00
39,00	6,20	3,60	88,00	90,00	57,00	68,00
44,00	6,30	3,60	85,00	93,00	58,00	70,00
23,00	7,20	4,00	79,00	95,00	58,00	70,00
34,00	5,70	3,20	84,00	93,00	59,00	70,00
36,00	6,30	3,50	77,00	101,00	59,00	73,00
37,00	4,90	2,60	92,00	94,00	62,00	73,00
24,00	7,60	4,00	83,00	97,00	65,00	76,00
36,00	7,70	4,00	102,00	98,00	65,00	76,00
33,00	7,70	4,00	85,00	103,00	63,00	76,00
42,00	6,10	3,10	98,00	93,00	67,00	76,00
27,00	5,70	3,10	76,00	107,00	63,00	78,00
43,00	6,70	3,50	84,00	107,00	72,00	84,00

43,00	8,10	4,20	93,00	118,00	81,00	93,00
24,00	6,70	3,60	68,00	108,00	66,00	80,00
27,00	7,30	3,90	76,00	100,00	60,00	73,00
19,00	8,40	4,30	86,00	109,00	68,00	82,00
17,00	8,20	4,20	85,00	104,00	69,00	81,00
28,00	6,20	3,20	66,00	105,00	61,00	76,00
30,00	6,00	3,10	63,00	100,00	61,00	74,00
35,00	7,50	3,80	88,00	108,00	72,00	84,00
36,00	7,00	3,50	92,00	108,00	72,00	84,00
29,00	6,30	3,60	85,00	111,00	71,00	84,00
34,00	6,50	3,70	88,00	104,00	66,00	79,00
23,00	7,40	4,10	83,00	117,00	71,00	86,00
44,00	6,70	3,60	82,00	118,00	78,00	91,00
24,00	7,50	4,00	93,00	122,00	84,00	97,00
34,00	7,50	4,10	80,00	107,00	71,00	83,00
30,00	7,70	4,10	89,00	104,00	72,00	83,00
37,00	7,00	3,70	85,00	103,00	72,00	82,00
44,00	8,60	4,40	105,00	108,00	75,00	86,00
38,00	5,40	3,60	84,00	95,00	59,00	71,00
39,00	5,60	3,60	97,00	99,00	68,00	78,00
42,00	6,20	3,80	98,00	100,00	66,00	77,00
45,00	6,20	3,70	98,00	105,00	78,00	87,00
25,00	6,30	3,40	69,00	100,00	58,00	72,00
30,00	7,10	3,90	82,00	96,00	61,00	73,00
28,00	7,60	4,00	85,00	98,00	64,00	75,00
22,00	5,90	3,00	68,00	104,00	65,00	78,00
23,00	5,10	3,10	68,00	91,00	55,00	67,00
22,00	4,70	2,90	64,00	95,00	56,00	69,00
21,00	6,00	3,70	79,00	97,00	59,00	72,00
32,00	5,50	3,30	69,00	105,00	67,00	80,00
36,00	5,60	3,30	76,00	98,00	60,00	73,00
49,00	5,50	3,20	80,00	97,00	60,00	72,00
71,00	5,00	2,90	110,00	94,00	65,00	75,00
64,00	5,50	3,10	116,00	98,00	69,00	79,00
73,00	5,70	3,20	122,00	103,00	73,00	83,00
30,00	5,80	3,80	96,00	88,00	59,00	69,00
29,00	5,70	3,60	99,00	95,00	65,00	75,00
37,00	5,30	3,20	95,00	103,00	69,00	80,00
37,00	5,80	3,40	101,00	111,00	77,00	88,00
23,00	6,00	3,50	77,00	91,00	57,00	68,00
46,00	6,10	3,50	72,00	89,00	56,00	67,00
35,00	5,30	3,00	66,00	95,00	67,00	76,00
41,00	5,40	3,00	70,00	103,00	68,00	80,00
45,00	6,30	3,60	95,00	92,00	59,00	70,00
55,00	5,30	3,00	105,00	94,00	67,00	76,00
58,00	6,20	3,40	91,00	93,00	64,00	74,00
45,00	6,40	3,50	91,00	96,00	66,00	76,00
24,00	7,60	4,30	97,00	111,00	76,00	88,00
33,00	6,60	3,70	87,00	107,00	73,00	84,00
33,00	7,30	4,10	106,00	131,00	90,00	104,00

43,00	6,90	3,80	108,00	121,00	88,00	99,00
18,00	7,60	4,50	111,00	101,00	72,00	82,00
23,00	7,70	4,40	126,00	108,00	81,00	90,00
17,00	10,10	5,70	133,00	118,00	81,00	93,00
53,00	7,90	4,30	128,00	108,00	81,00	90,00
29,00	6,30	3,80	86,00	97,00	62,00	74,00
30,00	6,20	3,70	74,00	104,00	66,00	79,00
45,00	7,00	4,10	93,00	108,00	73,00	85,00
36,00	6,90	4,00	95,00	98,00	66,00	77,00
38,00	7,10	4,00	76,00	108,00	67,00	81,00
33,00	6,40	3,40	82,00	107,00	70,00	82,00
34,00	6,40	3,40	89,00	100,00	68,00	79,00
48,00	5,00	2,60	102,00	116,00	81,00	93,00
21,00	7,70	3,90	73,00	97,00	67,00	77,00
25,00	8,80	4,50	74,00	108,00	70,00	83,00
22,00	10,90	5,40	93,00	113,00	76,00	88,00
21,00	11,00	5,40	101,00	107,00	74,00	85,00
29,00	5,10	3,10	92,00	97,00	66,00	76,00
41,00	5,30	3,20	91,00	93,00	62,00	72,00
40,00	4,30	2,60	82,00	96,00	62,00	73,00
40,00	5,10	3,00	96,00	95,00	63,00	74,00
20,00	8,00	4,40	81,00	91,00	53,00	66,00
20,00	9,50	5,10	107,00	100,00	57,00	71,00
18,00	7,50	4,00	85,00	101,00	49,00	66,00
21,00	7,90	4,20	86,00	95,00	57,00	70,00
21,00	8,90	4,60	91,00	90,00	62,00	71,00
29,00	4,00	2,50	58,00	87,00	57,00	67,00
40,00	4,30	2,60	68,00	92,00	60,00	71,00
27,00	4,20	2,60	60,00	89,00	58,00	68,00
44,00	4,50	2,80	80,00	90,00	61,00	71,00
30,00	5,80	3,20	89,00	100,00	67,00	78,00
39,00	5,70	3,10	83,00	92,00	58,00	69,00
34,00	5,50	3,00	74,00	95,00	59,00	71,00
20,00	6,00	3,30	69,00	96,00	63,00	74,00
30,00	6,40	3,60	77,00	95,00	58,00	70,00
34,00	6,30	3,50	76,00	93,00	57,00	69,00
40,00	6,30	3,40	76,00	93,00	59,00	70,00
41,00	7,30	3,90	82,00	105,00	67,00	80,00
41,00	7,60	4,10	87,00	109,00	75,00	86,00
38,00	7,40	4,00	83,00	93,00	53,00	66,00
43,00	7,20	3,70	80,00	90,00	57,00	68,00
40,00	7,40	3,90	76,00	97,00	60,00	72,00
35,00	7,90	4,10	81,00	90,00	62,00	71,00
42,00	7,70	4,00	85,00	98,00	66,00	77,00
27,00	6,30	3,50	80,00	91,00	62,00	72,00
38,00	6,60	3,70	85,00	92,00	64,00	73,00
41,00	6,40	3,40	69,00	93,00	65,00	74,00
38,00	5,60	3,00	64,00	98,00	71,00	80,00
24,00	10,10	4,80	87,00	109,00	57,00	74,00
25,00	10,00	4,70	81,00	120,00	72,00	88,00

26,00	10,30	4,80	86,00	119,00	72,00	88,00
30,00	9,60	4,40	90,00	120,00	70,00	87,00
48,00	9,70	4,30	91,00	124,00	83,00	97,00
25,00	6,90	3,80	72,00	94,00	59,00	71,00
23,00	8,00	4,40	80,00	94,00	62,00	73,00
22,00	7,70	4,20	74,00	101,00	65,00	77,00
21,00	5,70	3,00	75,00	104,00	62,00	76,00
37,00	7,70	4,10	83,00	103,00	71,00	82,00
27,00	6,30	3,60	75,00	96,00	60,00	72,00
38,00	5,90	3,40	72,00	97,00	61,00	73,00
23,00	6,20	3,50	82,00	100,00	66,00	77,00
48,00	5,70	3,10	88,00	107,00	79,00	88,00
41,00	5,90	3,20	74,00	115,00	81,00	92,00
27,00	5,20	3,30	87,00	101,00	65,00	77,00
28,00	4,60	2,80	74,00	97,00	67,00	77,00
39,00	5,00	3,00	80,00	99,00	71,00	80,00
30,00	4,70	2,80	60,00	100,00	67,00	78,00
30,00	4,30	2,50	59,00	98,00	68,00	78,00
26,00	4,90	2,90	70,00	91,00	52,00	65,00
26,00	6,00	3,50	73,00	106,00	61,00	76,00
33,00	5,70	3,30	72,00	96,00	58,00	71,00
27,00	6,90	3,90	82,00	98,00	65,00	76,00
24,00	10,10	5,50	104,00	103,00	73,00	83,00
36,00	9,40	5,00	96,00	110,00	74,00	86,00
25,00	7,70	4,10	84,00	102,00	68,00	79,00
33,00	9,50	5,10	100,00	116,00	82,00	93,00
43,00	9,00	4,70	89,00	118,00	82,00	94,00
23,00	6,10	3,50	69,00	97,00	61,00	73,00
25,00	6,90	4,00	73,00	96,00	64,00	75,00
24,00	7,20	4,10	77,00	97,00	68,00	78,00
27,00	6,50	3,70	75,00	99,00	61,00	74,00
46,00	5,50	3,10	79,00	104,00	69,00	81,00
37,00	5,80	3,40	78,00	108,00	67,00	81,00
38,00	6,40	3,70	89,00	103,00	69,00	80,00
46,00	6,40	3,70	89,00	107,00	73,00	84,00
37,00	6,30	3,60	73,00	105,00	66,00	79,00
35,00	5,80	3,30	80,00	102,00	62,00	75,00
29,00	6,80	3,80	85,00	100,00	63,00	75,00
41,00	6,20	3,40	73,00	100,00	67,00	78,00
42,00	5,80	3,20	82,00	100,00	74,00	83,00
22,00	8,10	4,30	77,00	105,00	65,00	78,00
26,00	8,70	4,50	86,00	97,00	66,00	76,00
36,00	7,40	3,80	108,00	104,00	76,00	85,00
21,00	8,90	4,50	115,00	96,00	66,00	76,00
44,00	8,20	4,10	112,00	106,00	75,00	85,00
36,00	4,90	3,00	63,00	89,00	57,00	68,00
40,00	4,50	2,70	65,00	91,00	59,00	70,00
42,00	5,70	3,30	99,00	92,00	65,00	74,00
37,00	5,80	3,30	95,00	97,00	69,00	78,00
39,00	5,60	3,10	85,00	107,00	74,00	85,00

34,00	6,30	3,30	81,00	102,00	64,00	77,00
41,00	6,10	3,30	73,00	96,00	59,00	71,00
33,00	6,30	3,30	68,00	95,00	60,00	72,00
42,00	6,50	3,40	73,00	106,00	65,00	79,00
51,00	6,50	3,40	80,00	104,00	70,00	81,00
37,00	5,00	2,90	83,00	104,00	73,00	83,00
21,00	6,90	3,90	88,00	95,00	64,00	74,00
36,00	6,10	3,40	82,00	103,00	73,00	83,00
38,00	7,70	4,30	103,00	105,00	75,00	85,00
46,00	5,80	3,20	83,00	107,00	77,00	87,00
34,00	6,20	3,10	66,00	108,00	60,00	76,00
38,00	6,70	3,30	65,00	99,00	61,00	74,00
44,00	6,90	3,40	64,00	104,00	65,00	78,00
45,00	6,30	3,10	61,00	101,00	64,00	76,00
49,00	6,20	3,00	63,00	105,00	67,00	80,00
33,00	9,20	4,20	63,00	109,00	66,00	80,00
37,00	10,00	4,50	83,00	110,00	66,00	81,00
36,00	10,40	4,60	84,00	115,00	76,00	89,00
37,00	8,60	3,80	91,00	113,00	79,00	90,00
34,00	8,40	3,60	84,00	116,00	76,00	89,00
34,00	5,70	3,20	72,00	99,00	59,00	72,00
33,00	5,70	3,20	73,00	98,00	62,00	74,00
40,00	5,10	2,80	64,00	87,00	53,00	64,00
45,00	4,90	2,70	75,00	91,00	62,00	72,00
40,00	5,70	3,00	80,00	94,00	64,00	74,00
33,00	5,90	4,00	98,00	92,00	58,00	69,00
41,00	4,80	3,20	85,00	98,00	60,00	73,00
40,00	5,10	3,30	98,00	93,00	64,00	74,00
34,00	5,00	3,20	84,00	99,00	65,00	76,00
71,00	4,70	3,00	89,00	107,00	68,00	81,00
39,00	4,10	2,60	79,00	93,00	55,00	68,00
34,00	5,40	3,40	85,00	91,00	56,00	68,00
41,00	4,00	2,50	68,00	92,00	59,00	70,00
47,00	4,90	3,00	73,00	101,00	59,00	73,00
43,00	4,10	2,50	71,00	99,00	67,00	78,00
44,00	5,80	3,30	69,00	91,00	58,00	69,00
26,00	7,40	4,10	90,00	100,00	64,00	76,00
41,00	7,40	4,10	89,00	100,00	70,00	80,00
44,00	6,60	3,60	74,00	105,00	66,00	79,00
41,00	5,40	3,10	74,00	93,00	62,00	72,00
31,00	6,90	3,90	91,00	106,00	71,00	83,00
40,00	5,60	3,10	77,00	105,00	70,00	82,00
43,00	5,10	2,80	72,00	108,00	75,00	86,00
36,00	6,20	3,40	73,00	92,00	60,00	71,00
34,00	7,70	4,20	89,00	93,00	62,00	72,00
41,00	7,30	4,00	110,00	87,00	61,00	70,00
50,00	7,40	4,00	82,00	94,00	68,00	77,00
58,00	6,20	3,30	93,00	59,00	41,00	47,00
36,00	5,80	3,30	69,00	92,00	54,00	67,00
26,00	7,20	3,90	89,00	98,00	65,00	76,00

28,00	5,80	3,10	70,00	92,00	61,00	71,00
41,00	6,90	3,70	95,00	95,00	65,00	75,00
39,00	5,80	3,10	88,00	84,00	57,00	66,00
30,00	6,60	3,70	81,00	93,00	56,00	68,00
46,00	5,80	3,20	86,00	98,00	61,00	73,00
34,00	6,00	3,30	77,00	106,00	66,00	79,00
37,00	5,80	3,20	89,00	100,00	69,00	79,00
39,00	4,60	2,90	89,00	95,00	58,00	70,00
47,00	4,70	2,90	102,00	99,00	66,00	77,00
26,00	4,50	2,70	103,00	92,00	67,00	75,00
60,00						
43,00	6,90	3,60	81,00	110,00	72,00	85,00
42,00	8,20	4,20	81,00	99,00	67,00	78,00
35,00	6,60	3,40	94,00	111,00	71,00	84,00
30,00	7,30	3,70	103,00	101,00	73,00	82,00
36,00	6,90	3,50	98,00	107,00	74,00	85,00
31,00	7,40	4,00	76,00	103,00	67,00	79,00
31,00	7,20	3,90	79,00	97,00	62,00	74,00
42,00	5,80	3,10	75,00	98,00	70,00	79,00
42,00	6,70	3,50	79,00	107,00	73,00	84,00
36,00	5,50	3,30	76,00	91,00	58,00	69,00
26,00	5,80	3,40	75,00	91,00	59,00	70,00
39,00	5,50	3,20	78,00	93,00	58,00	70,00
43,00	6,10	3,50	94,00	93,00	66,00	75,00
47,00	4,40	2,50	77,00	97,00	66,00	76,00
34,00	6,00	3,50	77,00	106,00	67,00	80,00
35,00	5,50	3,20	76,00	103,00	69,00	80,00
43,00	6,10	3,40	75,00	115,00	75,00	88,00
49,00	5,50	3,10	71,00	102,00	70,00	81,00
64,00	6,00	3,30	83,00	133,00	93,00	106,00
27,00	7,00	3,90	84,00	115,00	78,00	90,00
37,00	6,80	3,80	83,00	112,00	76,00	88,00
37,00	7,00	3,80	89,00	131,00	100,00	110,00
20,00	5,40	3,20	65,00	102,00	58,00	73,00
21,00	5,30	3,10	67,00	108,00	60,00	76,00
31,00	5,50	3,20	78,00	115,00	70,00	85,00
26,00	5,30	3,00	76,00	109,00	73,00	85,00
24,00	5,00	2,80	76,00	109,00	76,00	87,00
29,00	5,80	3,50	88,00	101,00	62,00	75,00
40,00	7,10	4,20	106,00	93,00	60,00	71,00
39,00	6,90	4,00	97,00	90,00	61,00	71,00
47,00	7,10	4,10	110,00	92,00	62,00	72,00

SVR_B	SVRI_B	SV_B	TFC_B	ACI_B	LCWI_B	PEP_B
860,00	1620,00	100,00	28,00	160,00	3,90	62,00
900,00	1720,00	107,00	31,00	161,00	4,30	72,00
890,00	1710,00	102,00	28,00	129,00	4,11	74,00
960,00	1870,00	91,00	30,00	82,00	4,05	73,00
870,00	1730,00	108,00	32,00	117,00	5,28	81,00
890,00	1460,00	68,00	25,00	183,00	5,04	53,00
1140,00	1880,00	52,00	26,00	117,00	4,02	62,00
970,00	1630,00	67,00	25,00	130,00	4,19	56,00
1010,00	1720,00	69,00	28,00	129,00	5,31	66,00
1060,00	1820,00	56,00	26,00	61,00	4,16	85,00
860,00	1610,00	83,00	23,00	116,00	3,50	81,00
950,00	1800,00	82,00	24,00	98,00	3,60	79,00
870,00	1660,00	85,00	32,00	112,00	3,39	93,00
1180,00	2270,00	88,00	30,00	122,00	3,50	100,00
1140,00	2210,00	85,00	32,00	113,00	4,13	99,00
1060,00	1890,00	75,00	26,00	141,00	3,60	73,00
1010,00	1810,00	69,00	27,00	106,00	3,40	110,00
1040,00	1900,00	72,00	26,00	112,00	3,49	91,00
930,00	1730,00	90,00	31,00	134,00	3,95	87,00
930,00	1700,00	81,00	31,00	92,00	2,52	74,00
870,00	1610,00	86,00	26,00	80,00	3,59	64,00
860,00	1630,00	91,00	30,00	76,00	4,52	54,00
880,00	1710,00	80,00	33,00	46,00	4,63	68,00
810,00	1610,00	96,00	36,00	101,00	5,42	43,00
1360,00	2350,00	64,00	30,00	97,00	2,61	91,00
1190,00	2090,00	69,00	33,00	102,00	2,86	76,00
940,00	1650,00	77,00	33,00	117,00	3,22	71,00
1140,00	2040,00	76,00	34,00	97,00	3,44	68,00
1520,00	2760,00	71,00	33,00	97,00	3,45	104,00
1280,00	2180,00	64,00	28,00	161,00	3,30	81,00
1070,00	1820,00	73,00	26,00	139,00	3,64	93,00
930,00	1600,00	77,00	27,00	157,00	4,72	96,00
860,00	1520,00	74,00	26,00	113,00	4,61	89,00
1130,00	2030,00	72,00	31,00	136,00	3,81	75,00
860,00	1700,00	80,00	27,00	99,00	5,86	89,00
790,00	1580,00	98,00	29,00	91,00	5,64	90,00
730,00	1500,00	99,00	35,00	104,00	5,02	90,00
760,00	1570,00	91,00	24,00	83,00	4,35	111,00
780,00	1640,00	90,00	26,00	86,00	5,07	108,00
1030,00	1770,00	73,00	27,00	162,00	2,82	57,00
830,00	1460,00	85,00	24,00	153,00	3,86	70,00
880,00	1580,00	82,00	29,00	142,00	3,66	72,00
840,00	1540,00	74,00	22,00	81,00	3,55	68,00
910,00	1680,00	88,00	25,00	115,00	3,76	83,00
870,00	1520,00	88,00	28,00	175,00	3,81	69,00
790,00	1410,00	87,00	29,00	194,00	3,68	84,00
880,00	1570,00	93,00	31,00	187,00	3,49	74,00
1110,00	2030,00	79,00	26,00	131,00	3,63	109,00
930,00	1710,00	93,00	35,00	118,00	4,31	68,00

820,00	1610,00	113,00	27,00	165,00	3,51	61,00
810,00	1620,00	103,00	20,00	92,00	3,09	65,00
690,00	1380,00	123,00	30,00	134,00	4,09	57,00
880,00	1780,00	114,00	30,00	138,00	3,35	74,00
1010,00	2050,00	104,00	26,00	110,00	3,25	93,00
780,00	1370,00	83,00	26,00	161,00	3,22	73,00
870,00	1560,00	80,00	29,00	241,00	2,93	98,00
940,00	1690,00	68,00	26,00	136,00	3,15	102,00
860,00	1580,00	70,00	23,00	111,00	2,71	113,00
870,00	1600,00	68,00	26,00	120,00	2,94	126,00
640,00	1330,00	122,00	29,00	148,00	5,02	64,00
730,00	1540,00	125,00	28,00	132,00	4,21	69,00
660,00	1410,00	120,00	28,00	141,00	4,46	76,00
660,00	1420,00	113,00	24,00	104,00	4,80	82,00
670,00	1470,00	104,00	24,00	87,00	4,17	94,00
1010,00	1770,00	80,00	25,00	161,00	2,59	81,00
950,00	1670,00	87,00	28,00	146,00	2,91	71,00
1540,00	2700,00	77,00	30,00	101,00	2,80	94,00
1070,00	1770,00	73,00	31,00	158,00	3,65	70,00
1000,00	1660,00	69,00	27,00	144,00	3,11	78,00
1090,00	1810,00	66,00	27,00	124,00	3,67	83,00
1140,00	1910,00	69,00	28,00	141,00	3,48	99,00
1050,00	1800,00	79,00	34,00	165,00	3,88	89,00
1230,00	1970,00	59,00	30,00	158,00	2,87	82,00
1180,00	1910,00	64,00	32,00	141,00	2,78	74,00
1040,00	1730,00	67,00	28,00	134,00	2,99	68,00
1110,00	1870,00	57,00	31,00	96,00	2,76	73,00
810,00	1480,00	92,00	25,00	151,00	4,15	62,00
910,00	1690,00	91,00	24,00	113,00	3,52	79,00
770,00	1460,00	88,00	23,00	114,00	3,54	77,00
890,00	1720,00	83,00	23,00	113,00	3,97	87,00
800,00	1550,00	94,00	28,00	117,00	4,28	78,00
750,00	1440,00	85,00	29,00	81,00	4,86	53,00
810,00	1570,00	91,00	26,00	81,00	4,12	72,00
1000,00	1970,00	56,00	25,00	41,00	3,38	120,00
730,00	1450,00	81,00	24,00	63,00	4,47	90,00
870,00	1720,00	89,00	28,00	57,00	4,49	82,00
780,00	1410,00	93,00	29,00	202,00	3,88	82,00
		78,00	21,00	97,00		92,00
860,00	1610,00	84,00	28,00	141,00	4,12	92,00
880,00	1640,00	68,00	26,00	72,00	3,53	117,00
860,00	1650,00	78,00	26,00	103,00	4,14	123,00
900,00	1590,00	86,00	26,00	159,00	3,26	79,00
890,00	1610,00	86,00	29,00	163,00	3,61	76,00
880,00	1620,00	79,00	27,00	115,00	3,39	110,00
850,00	1570,00	80,00	27,00	116,00	3,90	99,00
1100,00	2100,00	69,00	25,00	81,00	2,85	132,00
1020,00	1700,00	78,00	30,00	142,00	2,53	59,00
920,00	1530,00	70,00	26,00	110,00	2,80	86,00
930,00	1570,00	67,00	24,00	99,00	3,11	78,00

950,00	1620,00	57,00	26,00	85,00	3,29	102,00
800,00	1420,00	93,00	29,00	211,00	3,31	58,00
870,00	1550,00	91,00	26,00	158,00	3,14	60,00
980,00	1770,00	96,00	30,00	203,00	3,38	64,00
920,00	1670,00	94,00	28,00	204,00	4,42	65,00
1130,00	2110,00	90,00	30,00	169,00	3,76	95,00
800,00	1270,00	66,00	25,00	187,00	3,59	57,00
820,00	1330,00	73,00	27,00	211,00	2,91	76,00
850,00	1400,00	70,00	29,00	207,00	2,86	79,00
780,00	1330,00	68,00	26,00	145,00	3,45	97,00
700,00	1430,00	109,00	24,00	96,00	4,17	62,00
750,00	1550,00	92,00	23,00	90,00	3,55	53,00
750,00	1560,00	110,00	27,00	99,00	3,83	53,00
840,00	1790,00	106,00	26,00	72,00	4,65	68,00
1260,00	2060,00	75,00	29,00	150,00	3,06	60,00
1390,00	2310,00	77,00	24,00	141,00	2,95	64,00
1240,00	2080,00	77,00	28,00	128,00	3,03	72,00
1400,00	2370,00	69,00	27,00	111,00	3,27	91,00
1720,00	2960,00	65,00	22,00	78,00	2,75	102,00
850,00	1650,00	97,00	28,00	112,00	4,37	71,00
800,00	1570,00	90,00	27,00	62,00	3,91	56,00
680,00	1370,00	96,00	27,00	92,00	4,74	30,00
940,00	1880,00	76,00	27,00	80,00	3,53	120,00
570,00	1320,00	139,00	22,00	81,00	4,41	63,00
540,00	1260,00	146,00	23,00	97,00	4,33	64,00
600,00	1380,00	140,00	23,00	101,00	4,20	80,00
640,00	1480,00	113,00	22,00	80,00	3,91	91,00
		95,00	27,00	86,00		72,00
740,00	1510,00	104,00	34,00	96,00	3,84	61,00
730,00	1510,00	106,00	32,00	95,00	3,94	94,00
730,00	1540,00	83,00	33,00	67,00	3,98	102,00
710,00	1380,00	100,00	22,00	130,00	4,32	74,00
800,00	1560,00	92,00	21,00	114,00	4,83	82,00
770,00	1520,00	95,00	27,00	137,00	4,60	77,00
840,00	1700,00	93,00	27,00	102,00	4,34	107,00
880,00	1750,00	84,00	27,00	89,00	3,22	73,00
950,00	1910,00	94,00	30,00	116,00	5,45	78,00
880,00	1770,00	94,00	28,00	105,00	4,59	89,00
870,00	1800,00	99,00	28,00	100,00	4,30	92,00
820,00	1590,00	111,00	30,00	160,00	4,07	56,00
850,00	1670,00	111,00	27,00	153,00	3,98	81,00
890,00	1750,00	103,00	26,00	124,00	3,80	92,00
960,00	1940,00	99,00	29,00	107,00	3,71	104,00
910,00	1690,00	86,00	25,00	114,00	3,64	86,00
970,00	1840,00	80,00	26,00	80,00	3,80	102,00
970,00	1900,00	78,00	28,00	80,00	4,08	112,00
960,00	1920,00	83,00	29,00	79,00	4,12	99,00
960,00	1960,00	68,00	19,00	41,00	3,39	96,00
1010,00	1890,00	85,00	27,00	73,00	3,08	61,00
1050,00	1980,00	79,00	36,00	78,00	3,27	46,00

1000,00	1900,00	85,00	34,00	72,00	3,23	64,00
950,00	1810,00	89,00	36,00	83,00	3,47	46,00
910,00	1600,00	89,00	30,00	154,00	2,95	62,00
1010,00	1800,00	82,00	25,00	133,00	2,54	94,00
1090,00	1950,00	81,00	33,00	165,00	2,81	109,00
880,00	1610,00	63,00	32,00	101,00	3,71	118,00
940,00	1720,00	89,00	31,00	112,00	3,38	59,00
840,00	1550,00	72,00	36,00	83,00	3,86	55,00
840,00	1550,00	59,00	36,00	68,00	3,53	89,00
870,00	1630,00	65,00	37,00	50,00	3,86	73,00
840,00	1460,00	72,00	27,00	71,00	3,34	57,00
730,00	1290,00	83,00	32,00	91,00	4,12	58,00
720,00	1310,00	95,00	31,00	124,00	5,34	43,00
730,00	1350,00	77,00	33,00	85,00	4,30	34,00
1130,00	2110,00	68,00	31,00	60,00	3,40	118,00
770,00	1440,00	92,00	30,00	97,00	3,49	54,00
950,00	1790,00	75,00	31,00	89,00	3,06	53,00
920,00	1740,00	82,00	37,00	85,00	3,06	53,00
690,00	1310,00	88,00	32,00	126,00	3,83	66,00
1080,00	2070,00	76,00	27,00	48,00	2,50	89,00
850,00	1550,00	86,00	24,00	136,00	4,06	79,00
820,00	1530,00	83,00	27,00	147,00	4,12	93,00
860,00	1630,00	80,00	25,00	117,00	4,09	102,00
780,00	1490,00	80,00	23,00	97,00	4,48	91,00
820,00	1640,00	85,00	23,00	97,00	2,88	62,00
730,00	1460,00	99,00	22,00	122,00	3,75	66,00
810,00	1610,00	100,00	26,00	117,00	3,60	77,00
730,00	1470,00	77,00	27,00	72,00	4,41	84,00
990,00	2040,00	67,00	31,00	42,00	3,80	92,00
620,00	1220,00	106,00	34,00	168,00	4,25	68,00
650,00	1280,00	116,00	32,00	154,00	4,04	67,00
790,00	1590,00	99,00	32,00	103,00	3,66	74,00
670,00	1370,00	94,00	29,00	124,00	4,23	73,00
710,00	1480,00	111,00	32,00	108,00	4,26	71,00
1010,00	1730,00	71,00	29,00	148,00	2,47	82,00
850,00	1470,00	76,00	42,00	139,00	3,20	64,00
830,00	1430,00	73,00	31,00	132,00	3,10	89,00
840,00	1480,00	69,00	24,00	94,00	3,19	87,00
730,00	1310,00	92,00	30,00	124,00	3,59	69,00
930,00	1670,00	70,00	27,00	79,00	2,82	96,00
870,00	1590,00	85,00	28,00	112,00	3,25	76,00
1130,00	2090,00	55,00	28,00	80,00	2,47	108,00
760,00	1440,00	93,00	31,00	107,00	3,91	66,00
740,00	1430,00	78,00	31,00	73,00	3,96	87,00
750,00	1460,00	89,00	30,00	83,00	3,87	69,00
920,00	1810,00	65,00	31,00	54,00	3,11	93,00
1040,00	1950,00	76,00	30,00	123,00	3,07	59,00
940,00	1800,00	82,00	29,00	134,00	3,89	90,00

880,00	1680,00	91,00	31,00	143,00	5,20	82,00
910,00	1680,00	98,00	35,00	144,00	3,74	68,00
750,00	1430,00	97,00	35,00	118,00	3,62	50,00
740,00	1440,00	105,00	34,00	114,00	4,62	52,00
750,00	1470,00	100,00	35,00	89,00	4,40	65,00
930,00	1780,00	95,00	28,00	96,00	3,16	56,00
940,00	1830,00	97,00	28,00	116,00	2,91	89,00
860,00	1680,00	89,00	28,00	81,00	4,16	83,00
920,00	1840,00	78,00	28,00	63,00	3,80	81,00
1020,00	1800,00	77,00	28,00	155,00	3,89	62,00
920,00	1630,00	78,00	34,00	165,00	3,76	57,00
880,00	1610,00	89,00	27,00	158,00	4,59	65,00
1030,00	1950,00	82,00	28,00	121,00	4,28	72,00
990,00	1890,00	82,00	25,00	81,00	5,04	58,00
840,00	1550,00	96,00	33,00	168,00	4,39	83,00
820,00	1550,00	91,00	34,00	105,00	4,41	79,00
890,00	1690,00	85,00	31,00	112,00	3,93	102,00
770,00	1490,00	81,00	33,00	76,00	4,94	71,00
1000,00	1520,00	65,00	32,00	226,00	3,20	67,00
1050,00	1650,00	59,00	27,00	172,00	3,63	84,00
940,00	1520,00	64,00	29,00	165,00	3,81	86,00
1070,00	1780,00	61,00	35,00	134,00	4,24	93,00
860,00	1570,00	91,00	30,00	134,00	3,19	66,00
770,00	1420,00	90,00	31,00	137,00	3,64	76,00
750,00	1420,00	90,00	32,00	129,00	3,87	79,00
1010,00	1950,00	87,00	30,00	115,00	3,07	93,00
990,00	1600,00	77,00	29,00	199,00	2,67	61,00
1100,00	1780,00	77,00	32,00	181,00	2,57	61,00
900,00	1480,00	77,00	31,00	192,00	3,39	60,00
1110,00	1840,00	83,00	31,00	172,00	3,42	66,00
980,00	1650,00	77,00	34,00	158,00	3,13	81,00
1000,00	1680,00	69,00	33,00	113,00	2,98	80,00
1130,00	1960,00	47,00	32,00	91,00	2,80	113,00
1100,00	1930,00	47,00	37,00	75,00	3,17	114,00
1070,00	1890,00	47,00	35,00	95,00	3,61	118,00
900,00	1370,00	62,00	31,00	161,00	3,34	59,00
1000,00	1590,00	55,00	28,00	165,00	3,44	73,00
1150,00	1890,00	57,00	30,00	111,00	3,34	104,00
1160,00	1970,00	60,00	30,00	95,00	3,93	82,00
860,00	1470,00	78,00	32,00	216,00	3,00	61,00
830,00	1440,00	85,00	34,00	185,00	2,96	73,00
1090,00	1910,00	82,00	33,00	138,00	2,96	88,00
1130,00	2000,00	76,00	34,00	133,00	3,15	96,00
840,00	1480,00	66,00	34,00	143,00	3,18	107,00
1100,00	1970,00	50,00	33,00	138,00	2,86	133,00
900,00	1630,00	70,00	33,00	162,00	3,28	124,00
900,00	1640,00	72,00	32,00	161,00	3,44	118,00
890,00	1560,00	78,00	33,00	160,00	4,95	48,00
980,00	1740,00	73,00	26,00	110,00	4,02	86,00
1100,00	1980,00	69,00	37,00	65,00	5,60	67,00

1090,00	2010,00	66,00	36,00	79,00	4,96	84,00
820,00	1390,00	72,00	31,00	85,00	4,77	35,00
890,00	1540,00	61,00	31,00	88,00	5,27	46,00
710,00	1270,00	77,00	39,00	96,00	6,88	25,00
870,00	1600,00	64,00	33,00	96,00	5,09	70,00
880,00	1480,00	82,00	35,00	272,00	3,60	56,00
960,00	1630,00	84,00	28,00	197,00	3,76	67,00
920,00	1580,00	76,00	33,00	208,00	4,53	99,00
840,00	1470,00	76,00	30,00	187,00	3,95	104,00
870,00	1540,00	94,00	28,00	175,00	4,21	67,00
960,00	1800,00	77,00	29,00	70,00	3,69	55,00
950,00	1800,00	73,00	29,00	80,00	3,40	62,00
1410,00	2740,00	51,00	29,00	55,00	3,18	99,00
760,00	1470,00	108,00	33,00	122,00	3,94	58,00
710,00	1400,00	117,00	33,00	188,00	4,88	54,00
620,00	1250,00	116,00	32,00	138,00	6,19	59,00
590,00	1210,00	112,00	32,00	115,00	5,96	56,00
1130,00	1860,00	61,00	28,00	163,00	3,04	64,00
1020,00	1700,00	58,00	29,00	142,00	2,96	62,00
1270,00	2110,00	53,00	29,00	121,00	2,44	85,00
1110,00	1860,00	58,00	37,00	96,00	2,86	110,00
620,00	1130,00	94,00	30,00	144,00	3,67	44,00
560,00	1040,00	90,00	31,00	171,00	4,68	41,00
660,00	1230,00	93,00	31,00	169,00	3,37	47,00
670,00	1260,00	92,00	32,00	129,00	3,73	45,00
610,00	1160,00	99,00	31,00	105,00	4,19	49,00
1270,00	2010,00	70,00	29,00	159,00	2,13	78,00
1260,00	2030,00	65,00	28,00	131,00	2,39	92,00
1210,00	1970,00	70,00	25,00	132,00	2,25	79,00
1190,00	1950,00	58,00	29,00	106,00	2,50	114,00
1030,00	1850,00	69,00	31,00	124,00	3,22	85,00
900,00	1640,00	71,00	32,00	123,00	2,79	76,00
970,00	1770,00	78,00	32,00	125,00	2,74	82,00
940,00	1720,00	85,00	31,00	89,00	3,10	96,00
820,00	1480,00	86,00	22,00	138,00	3,19	81,00
830,00	1500,00	85,00	28,00	176,00	3,05	66,00
840,00	1530,00	85,00	24,00	134,00	3,07	99,00
830,00	1550,00	88,00	26,00	157,00	4,07	98,00
860,00	1620,00	83,00	27,00	138,00	4,55	108,00
670,00	1260,00	97,00	31,00	144,00	3,30	72,00
710,00	1370,00	91,00	31,00	135,00	3,23	107,00
730,00	1400,00	98,00	30,00	142,00	3,58	92,00
680,00	1310,00	96,00	31,00	140,00	3,71	96,00
760,00	1470,00	88,00	29,00	116,00	3,96	102,00
860,00	1540,00	79,00	31,00	122,00	3,27	67,00
840,00	1520,00	80,00	32,00	114,00	3,40	77,00
880,00	1630,00	93,00	33,00	105,00	3,26	68,00
1090,00	2050,00	86,00	32,00	95,00	3,07	78,00
550,00	1150,00	122,00	29,00	157,00	4,64	62,00
670,00	1440,00	128,00	27,00	117,00	5,37	76,00

660,00	1420,00	120,00	26,00	104,00	5,47	82,00
690,00	1530,00	110,00	29,00	86,00	4,94	99,00
770,00	1740,00	104,00	28,00	100,00	5,49	111,00
780,00	1400,00	95,00	34,00	228,00	3,48	71,00
690,00	1260,00	102,00	33,00	233,00	4,11	59,00
760,00	1390,00	103,00	34,00	207,00	4,17	67,00
1020,00	1890,00	77,00	35,00	82,00	2,99	58,00
810,00	1530,00	94,00	36,00	127,00	4,35	81,00
880,00	1510,00	84,00	27,00	212,00	3,33	71,00
930,00	1620,00	83,00	27,00	181,00	3,19	86,00
940,00	1670,00	79,00	28,00	141,00	3,48	71,00
1200,00	2170,00	67,00	28,00	92,00	3,56	115,00
1200,00	2200,00	81,00	30,00	105,00	3,87	104,00
1110,00	1790,00	62,00	34,00	158,00	3,25	77,00
1260,00	2070,00	66,00	34,00	123,00	2,80	77,00
1210,00	1990,00	62,00	36,00	135,00	3,16	88,00
1260,00	2110,00	75,00	36,00	121,00	2,83	80,00
1390,00	2370,00	72,00	37,00	108,00	2,51	84,00
980,00	1650,00	72,00	30,00	215,00	2,42	62,00
970,00	1680,00	78,00	27,00	185,00	3,36	78,00
940,00	1630,00	80,00	31,00	158,00	2,98	96,00
840,00	1480,00	83,00	33,00	137,00	3,80	89,00
620,00	1160,00	100,00	30,00	193,00	5,91	51,00
700,00	1320,00	101,00	32,00	175,00	5,58	53,00
780,00	1460,00	92,00	32,00	113,00	4,20	71,00
750,00	1400,00	97,00	35,00	145,00	6,23	52,00
800,00	1530,00	102,00	36,00	109,00	5,84	31,00
910,00	1570,00	87,00	31,00	193,00	3,30	64,00
820,00	1440,00	92,00	31,00	177,00	3,81	58,00
820,00	1460,00	94,00	31,00	146,00	4,09	58,00
860,00	1530,00	90,00	28,00	147,00	3,47	67,00
1120,00	2020,00	70,00	31,00	63,00	3,20	108,00
1070,00	1810,00	74,00	34,00	154,00	3,59	86,00
950,00	1640,00	75,00	35,00	147,00	3,84	85,00
1000,00	1720,00	75,00	36,00	136,00	4,08	88,00
950,00	1670,00	83,00	27,00	162,00	3,67	83,00
970,00	1730,00	74,00	26,00	170,00	3,17	73,00
840,00	1510,00	82,00	28,00	127,00	3,64	85,00
950,00	1730,00	84,00	27,00	138,00	3,44	96,00
1080,00	2000,00	73,00	26,00	122,00	3,41	125,00
730,00	1400,00	106,00	37,00	176,00	4,27	59,00
650,00	1250,00	106,00	40,00	127,00	4,50	54,00
870,00	1720,00	71,00	36,00	90,00	4,17	105,00
640,00	1270,00	80,00	35,00	101,00	4,44	66,00
800,00	1600,00	73,00	38,00	76,00	4,50	106,00
1040,00	1720,00	78,00	30,00	193,00	2,58	82,00
1170,00	1940,00	66,00	28,00	151,00	2,43	92,00
990,00	1710,00	60,00	30,00	115,00	3,10	86,00
1000,00	1780,00	64,00	27,00	88,00	3,36	89,00
1140,00	2070,00	69,00	35,00	118,00	3,48	130,00

940,00	1780,00	78,00	33,00	102,00	3,27	83,00
870,00	1630,00	88,00	33,00	122,00	2,98	78,00
870,00	1660,00	87,00	34,00	84,00	3,03	84,00
920,00	1750,00	91,00	35,00	101,00	3,50	80,00
940,00	1810,00	83,00	34,00	131,00	3,58	107,00
1290,00	2260,00	60,00	39,00	75,00	3,02	92,00
820,00	1430,00	78,00	34,00	73,00	3,72	62,00
1050,00	1870,00	73,00	34,00	63,00	3,65	85,00
850,00	1540,00	75,00	35,00	50,00	4,67	71,00
1150,00	2100,00	70,00	34,00	73,00	3,59	100,00
920,00	1840,00	93,00	36,00	145,00	3,07	61,00
830,00	1670,00	104,00	34,00	136,00	3,18	77,00
850,00	1720,00	110,00	37,00	103,00	3,47	86,00
910,00	1860,00	104,00	38,00	115,00	3,03	88,00
990,00	2060,00	97,00	38,00	73,00	3,06	95,00
660,00	1450,00	130,00	29,00	124,00	4,36	78,00
610,00	1360,00	123,00	31,00	100,00	4,75	96,00
650,00	1490,00	125,00	31,00	86,00	5,33	101,00
800,00	1850,00	97,00	33,00	56,00	4,39	123,00
810,00	1880,00	106,00	33,00	59,00	4,23	116,00
950,00	1710,00	80,00	36,00	81,00	2,93	75,00
980,00	1750,00	84,00	28,00	109,00	3,04	78,00
940,00	1710,00	81,00	34,00	91,00	2,27	87,00
1100,00	2030,00	69,00	37,00	79,00	2,47	111,00
980,00	1850,00	69,00	34,00	102,00	2,88	105,00
880,00	1300,00	61,00	33,00	230,00	3,52	55,00
1140,00	1740,00	58,00	34,00	148,00	2,97	60,00
1110,00	1690,00	52,00	32,00	99,00	3,14	80,00
1150,00	1780,00	58,00	35,00	106,00	3,18	64,00
1300,00	2050,00	55,00	35,00	101,00	3,15	83,00
1240,00	1940,00	53,00	32,00	152,00	2,28	58,00
940,00	1500,00	68,00	76,00	412,00	2,95	103,00
1330,00	2120,00	60,00	32,00	108,00	2,22	85,00
1140,00	1860,00	64,00	35,00	144,00	2,78	77,00
1450,00	2360,00	58,00	34,00	139,00	2,53	114,00
890,00	1570,00	87,00	31,00	124,00	2,90	69,00
770,00	1390,00	83,00	33,00	136,00	4,07	60,00
830,00	1490,00	84,00	33,00	114,00	4,24	77,00
910,00	1670,00	89,00	35,00	126,00	3,67	71,00
1010,00	1750,00	76,00	33,00	152,00	2,87	78,00
930,00	1640,00	78,00	32,00	130,00	4,16	74,00
1120,00	2020,00	72,00	32,00	128,00	3,30	87,00
1300,00	2360,00	73,00	35,00	86,00	3,12	93,00
870,00	1580,00	86,00	29,00	169,00	3,08	72,00
700,00	1290,00	90,00	31,00	169,00	3,88	90,00
720,00	1330,00	70,00	29,00	163,00	3,55	103,00
790,00	1480,00	87,00	32,00	173,00	3,91	97,00
560,00	1050,00	69,00	32,00	121,00	1,84	117,00
870,00	1550,00	84,00	32,00	134,00	2,76	84,00
790,00	1440,00	82,00	39,00	128,00	3,91	35,00

930,00	1710,00	85,00	36,00	127,00	2,84	74,00
820,00	1540,00	72,00	35,00	69,00	3,55	112,00
850,00	1600,00	73,00	35,00	89,00	2,60	120,00
780,00	1400,00	79,00	29,00	85,00	3,16	49,00
950,00	1700,00	71,00	30,00	65,00	3,03	73,00
1000,00	1810,00	73,00	30,00	74,00	3,39	80,00
1030,00	1890,00	67,00	30,00	90,00	3,24	100,00
1150,00	1850,00	56,00	28,00	147,00	2,55	76,00
1250,00	2040,00	51,00	30,00	126,00	2,84	87,00
1290,00	2150,00	44,00	32,00	94,00	2,54	81,00
940,00	1790,00	85,00	22,00	87,00	4,02	79,00
730,00	1400,00	99,00	30,00	99,00	4,27	71,00
960,00	1890,00	71,00	27,00	51,00	3,71	122,00
850,00	1700,00	74,00	29,00	36,00	3,92	106,00
940,00	1870,00	74,00	28,00	45,00	3,85	105,00
810,00	1510,00	97,00	36,00	116,00	4,07	62,00
770,00	1430,00	93,00	33,00	132,00	3,73	61,00
1030,00	1920,00	77,00	33,00	115,00	3,19	118,00
960,00	1820,00	81,00	36,00	107,00	3,85	99,00
940,00	1560,00	72,00	36,00	150,00	2,92	76,00
910,00	1540,00	78,00	36,00	167,00	3,06	71,00
960,00	1630,00	70,00	35,00	123,00	2,89	84,00
920,00	1600,00	68,00	37,00	110,00	3,43	91,00
1300,00	2270,00	59,00	34,00	77,00	2,48	92,00
1020,00	1750,00	75,00	29,00	128,00	3,60	92,00
1100,00	1910,00	77,00	33,00	156,00	3,29	111,00
1110,00	1970,00	81,00	28,00	137,00	3,93	91,00
1110,00	2000,00	82,00	30,00	150,00	3,23	112,00
1360,00	2460,00	74,00	29,00	130,00	4,69	114,00
980,00	1750,00	85,00	32,00	128,00	4,63	70,00
990,00	1780,00	86,00	28,00	145,00	4,35	83,00
1210,00	2210,00	78,00	30,00	93,00	5,63	66,00
1020,00	1730,00	87,00	34,00	191,00	2,98	56,00
1070,00	1850,00	88,00	33,00	193,00	3,04	62,00
1170,00	2050,00	77,00	32,00	149,00	3,50	90,00
1220,00	2170,00	76,00	35,00	148,00	3,32	86,00
1310,00	2340,00	68,00	34,00	132,00	3,23	106,00
980,00	1620,00	65,00	34,00	111,00	3,37	70,00
760,00	1280,00	70,00	35,00	115,00	3,80	82,00
770,00	1330,00	71,00	35,00	120,00	3,66	100,00
760,00	1330,00	66,00	34,00	109,00	3,78	106,00

LVET_B	VI_B	STR_B	Gestational age_weeks	Birth weight
296,00	89,00	21,00	42,00	3705,00
311,00	97,00	23,00		
270,00	95,00	28,00		
267,00	63,00	29,00		
288,00	83,00	29,00		
230,00	90,00	23,00	41,00	4160,00
168,00	70,00	37,00		
226,00	78,00	26,00		
239,00	76,00	28,00		
195,00	64,00	45,00		
289,00	58,00	28,00	38,00	3060,00
293,00	51,00	27,00		
276,00	61,00	34,00		
299,00	60,00	34,00		
287,00	53,00	34,00		
242,00	76,00	31,00	36,00	2830,00
262,00	60,00	42,00		
245,00	61,00	37,00		
296,00	78,00	29,00		
294,00	60,00	26,00	40,00	4250,00
286,00	69,00	23,00		
275,00	67,00	20,00		
251,00	50,00	29,00		
235,00	69,00	18,00		
290,00	51,00	31,00	39,00	2930,00
268,00	58,00	28,00		
275,00	77,00	27,00		
257,00	66,00	27,00		
281,00	54,00	37,00		
221,00	78,00	38,00	40,00	3778,00
282,00	85,00	34,00		
280,00	86,00	34,00		
261,00	71,00	34,00		
218,00	79,00	37,00		
209,00	64,00	43,00	40,00	3952,00
248,00	77,00	38,00		
224,00	80,00	41,00		
253,00	53,00	44,00		
246,00	51,00	44,00		
217,00	89,00	27,00	40,00	3255,00
283,00	100,00	25,00		
247,00	88,00	30,00		
244,00	59,00	28,00		
291,00	78,00	29,00		
303,00	102,00	23,00	41,00	4180,00
269,00	116,00	31,00		
284,00	123,00	26,00		
273,00	76,00	40,00		
294,00	83,00	24,00		

310,00	94,00	20,00	41,00	3950,00
317,00	59,00	21,00		
316,00	96,00	18,00		
318,00	78,00	23,00		
323,00	59,00	29,00		
291,00	86,00	25,00	38,00	3334,00
249,00	103,00	39,00		
247,00	60,00	42,00		
249,00	60,00	46,00		
240,00	60,00	52,00		
267,00	91,00	24,00	39,00	3564,00
305,00	79,00	23,00		
244,00	86,00	32,00		
258,00	62,00	32,00		
233,00	58,00	40,00		
302,00	84,00	27,00	38,00	2898,00
319,00	90,00	22,00		
328,00	66,00	29,00		
295,00	82,00	24,00	40,00	3728,00
281,00	80,00	28,00		
253,00	83,00	33,00		
269,00	89,00	37,00		
287,00	102,00	31,00		
252,00	77,00	33,00	40,00	3814,00
293,00	70,00	26,00		
262,00	72,00	26,00		
202,00	66,00	38,00		
273,00	88,00	23,00	41,00	3566,00
296,00	75,00	27,00		
275,00	64,00	28,00		
261,00	58,00	34,00		
256,00	75,00	31,00		
228,00	64,00	24,00	40,00	4360,00
264,00	62,00	27,00		
189,00	39,00	68,00		
236,00	55,00	39,00		
249,00	59,00	34,00		
299,00	109,00	28,00	39,00	3914,00
257,00	74,00	36,00		
249,00	84,00	38,00		
235,00	56,00	50,00		
242,00	69,00	51,00		
281,00	101,00	29,00	40,00	3118,00
256,00	98,00	31,00		
272,00	74,00	41,00		
259,00	74,00	38,00		
260,00	48,00	51,00		
319,00	88,00	19,00	41,00	3496,00
277,00	89,00	31,00		
248,00	76,00	32,00		

200,00	72,00	51,00		
296,00	109,00	20,00	39,00	2820,00
309,00	90,00	20,00		
310,00	107,00	21,00		
270,00	108,00	24,00		
286,00	84,00	33,00		
248,00	98,00	23,00	40,00	3320,00
268,00	117,00	29,00		
239,00	120,00	34,00		
234,00	89,00	41,00		
280,00	65,00	23,00	39,00	3930,00
221,00	56,00	25,00		
262,00	66,00	20,00		
276,00	52,00	25,00		
334,00	82,00	18,00	41,00	2988,00
354,00	75,00	18,00		
314,00	83,00	23,00		
299,00	67,00	30,00		
301,00	55,00	34,00		
284,00	69,00	26,00	42,00	3788,00
283,00	52,00	21,00		
224,00	64,00	14,00		
244,00	47,00	50,00		
294,00	58,00	22,00	41,00	3650,00
294,00	63,00	22,00		
288,00	62,00	28,00		
261,00	43,00	35,00		
266,00	59,00	28,00	40,00	4110,00
248,00	72,00	26,00		
279,00	66,00	34,00		
206,00	53,00	51,00		
275,00	83,00	28,00	41,00	3740,00
254,00	72,00	33,00		
214,00	91,00	37,00		
262,00	62,00	42,00		
241,00	53,00	31,00	39,00	3614,00
236,00	70,00	35,00		
268,00	58,00	33,00		
270,00	60,00	35,00		
311,00	89,00	19,00	40,00	3294,00
311,00	99,00	26,00		
298,00	81,00	31,00		
280,00	69,00	37,00		
295,00	67,00	29,00	40,00	3240,00
273,00	56,00	37,00		
255,00	50,00	44,00		
254,00	51,00	40,00		
234,00	32,00	41,00		
312,00	53,00	20,00	39,00	3430,00
262,00	50,00	18,00		

287,00	53,00	23,00		
271,00	60,00	17,00		
300,00	99,00	22,00	39,00	3110,00
303,00	82,00	31,00		
277,00	95,00	40,00		
196,00	68,00	60,00		
289,00	76,00	20,00	42,00	4066,00
209,00	62,00	27,00		
212,00	46,00	47,00		
219,00	46,00	38,00		
253,00	66,00	23,00	41,00	3230,00
266,00	82,00	22,00		
282,00	99,00	16,00		
220,00	58,00	16,00		
247,00	49,00	48,00		
294,00	65,00	19,00	39,00	3560,00
240,00	51,00	23,00		
299,00	46,00	18,00		
228,00	83,00	29,00		
277,00	45,00	32,00		
289,00	70,00	27,00	37,00	3650,00
274,00	70,00	34,00		
270,00	60,00	38,00		
246,00	61,00	38,00		
226,00	55,00	28,00	39,00	3270,00
267,00	68,00	25,00		
255,00	77,00	31,00		
196,00	55,00	44,00		
174,00	44,00	56,00		
263,00	92,00	26,00	40,00	4164,00
300,00	97,00	22,00		
246,00	71,00	31,00		
189,00	80,00	39,00		
264,00	71,00	27,00		
			42,00	3560,00
267,00	78,00	31,00		
291,00	70,00	23,00		
261,00	85,00	35,00		
258,00	66,00	34,00		
303,00	96,00	23,00	40,00	3760,00
262,00	55,00	37,00		
270,00	81,00	29,00		
181,00	52,00	60,00		
282,00	68,00	24,00	41,00	4550,00
241,00	55,00	36,00		
263,00	59,00	27,00		
233,00	36,00	42,00		
205,00	68,00	29,00	38,00	3480,00
211,00	86,00	43,00		

270,00	75,00	31,00		
293,00	94,00	23,00	40,00	4128,00
245,00	89,00	21,00		
287,00	81,00	19,00		
258,00	76,00	26,00		
279,00	67,00	20,00	40,00	3150,00
293,00	73,00	30,00		
252,00	62,00	33,00		
212,00	52,00	39,00		
204,00	106,00	31,00	40,00	2800,00
207,00	98,00	28,00		
240,00	106,00	29,00		
205,00	87,00	37,00		
214,00	67,00	28,00		
294,00	103,00	28,00	41,00	4002,00
274,00	77,00	29,00		
259,00	75,00	40,00		
224,00	58,00	33,00		
287,00	115,00	24,00	39,00	3100,00
248,00	92,00	34,00		
252,00	92,00	34,00		
236,00	75,00	40,00		
279,00	85,00	24,00	41,00	3910,00
270,00	88,00	28,00		
257,00	82,00	31,00		
247,00	73,00	39,00		
297,00	119,00	21,00	40,00	3758,00
321,00	107,00	19,00		
273,00	117,00	22,00		
302,00	128,00	22,00		
277,00	108,00	30,00		
265,00	77,00	31,00	40,00	3726,00
171,00	56,00	66,00		
171,00	51,00	68,00		
151,00	64,00	81,00		
261,00	105,00	23,00	38,00	3190,00
176,00	106,00	41,00		
242,00	72,00	43,00		
217,00	67,00	39,00		
251,00	105,00	25,00	39,00	3060,00
306,00	99,00	24,00		
312,00	84,00	28,00		
273,00	79,00	36,00		
251,00	64,00	43,00	40,00	2812,00
193,00	50,00	72,00		
250,00	70,00	50,00		
257,00	69,00	46,00		
212,00	89,00	23,00	38,00	3082,00
268,00	65,00	32,00		
252,00	51,00	27,00		

233,00	51,00	38,00		
238,00	72,00	15,00	40,00	3610,00
198,00	63,00	26,00		
235,00	63,00	11,00		
185,00	61,00	41,00		
261,00	133,00	22,00	39,00	3252,00
287,00	123,00	23,00		
243,00	131,00	41,00		
237,00	121,00	44,00		
301,00	117,00	22,00		
249,00	52,00	22,00	38,00	2915,00
232,00	51,00	28,00		
193,00	31,00	53,00		
310,00	78,00	19,00	38,00	3410,00
280,00	113,00	20,00		
258,00	94,00	23,00		
247,00	82,00	23,00		
203,00	83,00	32,00	39,00	3680,00
190,00	75,00	33,00		
187,00	71,00	46,00		
247,00	64,00	45,00		
286,00	85,00	15,00	40,00	3670,00
210,00	98,00	20,00		
213,00	101,00	23,00		
193,00	101,00	23,00		
236,00	92,00	21,00		
320,00	99,00	24,00	39,00	2905,00
274,00	90,00	34,00		
323,00	79,00	25,00		
242,00	78,00	47,00		
189,00	84,00	45,00	40,00	3670,00
190,00	79,00	41,00		
222,00	84,00	38,00		
283,00	83,00	34,00		
314,00	77,00	26,00	42,00	3270,00
250,00	88,00	27,00		
291,00	81,00	35,00		
249,00	102,00	40,00		
240,00	88,00	45,00		
285,00	86,00	25,00	42,00	3988,00
274,00	81,00	39,00		
295,00	84,00	31,00		
276,00	82,00	35,00		
272,00	71,00	39,00		
253,00	81,00	28,00	40,00	3285,00
257,00	79,00	31,00		
311,00	71,00	22,00		
293,00	59,00	27,00		
257,00	91,00	25,00	41,00	3810,00
289,00	84,00	26,00		

262,00	77,00	31,00		
248,00	62,00	40,00		
223,00	56,00	50,00		
310,00	111,00	23,00	41,00	4180,00
295,00	127,00	20,00		
298,00	130,00	23,00		
275,00	49,00	22,00		
262,00	95,00	31,00		
285,00	115,00	25,00	41,00	3330,00
299,00	99,00	29,00		
237,00	92,00	31,00		
233,00	68,00	52,00		
315,00	63,00	33,00		
249,00	90,00	32,00	38,00	2780,00
298,00	67,00	26,00		
252,00	75,00	36,00		
323,00	80,00	25,00		
309,00	67,00	27,00		
213,00	114,00	30,00	38,00	3610,00
249,00	111,00	32,00		
258,00	123,00	38,00		
271,00	102,00	33,00		
266,00	105,00	19,00	40,00	4040,00
256,00	99,00	21,00		
266,00	84,00	27,00		
264,00	88,00	20,00		
298,00	71,00	11,00		
298,00	110,00	22,00	41,00	3586,00
296,00	118,00	20,00		
293,00	118,00	20,00		
291,00	93,00	23,00		
238,00	67,00	46,00		
283,00	91,00	30,00	33,00	2251,00
258,00	92,00	34,00		
275,00	83,00	32,00		
280,00	102,00	30,00	39,00	3530,00
192,00	105,00	38,00		
247,00	98,00	35,00		
277,00	87,00	34,00		
248,00	71,00	50,00		
290,00	102,00	21,00	41,00	3396,00
279,00	97,00	20,00		
195,00	59,00	55,00		
181,00	70,00	37,00		
196,00	58,00	55,00		
321,00	107,00	26,00	41,00	3680,00
262,00	86,00	37,00		
192,00	73,00	45,00		
224,00	61,00	40,00		
259,00	65,00	50,00		

244,00	63,00	35,00	41,00	3895,00
273,00	72,00	29,00		
304,00	58,00	28,00		
293,00	66,00	28,00		
260,00	66,00	42,00		
271,00	45,00	34,00	39,00	3762,00
292,00	68,00	22,00		
271,00	63,00	32,00		
278,00	59,00	28,00		
265,00	53,00	38,00		
212,00	77,00	29,00	41,00	3720,00
275,00	75,00	29,00		
312,00	72,00	28,00		
281,00	69,00	32,00		
298,00	49,00	32,00		
277,00	81,00	29,00	41,00	4484,00
262,00	70,00	37,00		
259,00	66,00	40,00		
235,00	42,00	53,00		
250,00	44,00	47,00		
303,00	65,00	25,00	41,00	4450,00
307,00	77,00	25,00		
315,00	61,00	28,00		
286,00	47,00	39,00		
261,00	47,00	41,00		
263,00	118,00	21,00	40,00	3690,00
254,00	90,00	24,00		
255,00	68,00	32,00		
261,00	76,00	25,00		
245,00	72,00	35,00		
196,00	80,00	30,00	41,00	2930,00
241,00	189,00	42,00		
283,00	69,00	30,00		
255,00	84,00	31,00		
267,00	70,00	43,00		
312,00	84,00	22,00	39,00	3240,00
242,00	88,00	25,00		
273,00	81,00	28,00		
278,00	83,00	26,00		
268,00	83,00	30,00	38,00	3180,00
279,00	75,00	27,00		
247,00	66,00	37,00		
258,00	60,00	36,00		
262,00	91,00	28,00	41,00	4292,00
265,00	108,00	34,00		
212,00	76,00	49,00		
249,00	92,00	39,00		
221,00	63,00	53,00		
298,00	89,00	28,00	38,00	3835,00
239,00	70,00	15,00		

238,00	90,00	32,00		
253,00	54,00	44,00		
241,00	63,00	51,00		
270,00	62,00	18,00	38,00	2980,00
271,00	50,00	27,00		
262,00	57,00	31,00		
250,00	50,00	40,00		
204,00	81,00	38,00	38,00	3740,00
195,00	67,00	45,00		
175,00	50,00	48,00		
256,00	65,00	31,00	39,00	3290,00
302,00	71,00	24,00		
241,00	46,00	51,00		
236,00	44,00	46,00		
242,00	43,00	44,00		
306,00	84,00	21,00	38,00	3175,00
284,00	84,00	22,00		
269,00	66,00	44,00		
256,00	66,00	39,00		
274,00	92,00	28,00	40,00	3180,00
281,00	103,00	26,00		
272,00	76,00	31,00		
249,00	70,00	37,00		
249,00	47,00	37,00		
303,00	76,00	31,00	39,00	3382,00
302,00	85,00	37,00		
273,00	87,00	33,00		
295,00	84,00	38,00		
276,00	64,00	42,00		
285,00	87,00	25,00	32,00	1815,00
294,00	87,00	28,00		
232,00	74,00	29,00		
313,00	110,00	18,00	39,00	3300,00
312,00	100,00	20,00		
254,00	91,00	35,00		
240,00	88,00	37,00		
238,00	68,00	44,00		
256,00	73,00	28,00	39,00	3880,00
259,00	90,00	32,00		
259,00	87,00	39,00		
224,00	84,00	47,00		

Placenta weight	5-minute Apgar s	Umbilical artery pH	Umbilical artery base excess
560,00	9,00	7,21	7,20
630,00	10,00	7,14	-8,50
600,00	10,00		
460,00	8,00		
620,00	10,00	7,41	-4,30
420,00	10,00	7,24	-2,50
560,00	10,00		
830,00	10,00	7,33	1,30
	10,00		
870,00	9,00		

600,00	10,00	7,14	-7,20
460,00	10,00	7,23	-8,80
640,00	10,00	7,29	-3,10
620,00	9,00	7,32	-3,80
650,00	10,00		
600,00	9,00		
600,00	9,00		
1000,00	7,00	7,17	-4,30
620,00	10,00	7,41	-4,30
580,00	10,00		
460,00	10,00		

600,00	9,00		
500,00	9,00		
640,00	8,00	7,27	-,80
460,00	8,00		
680,00	10,00	7,28	-,60
520,00	10,00		
790,00	10,00		
	10,00		
650,00	10,00	7,27	,00
500,00	10,00		
475,00	9,00		
660,00	10,00	7,29	-,90

610,00	10,00		
600,00	10,00	7,35	-2,80
580,00	10,00	7,19	-10,20
600,00	10,00		
560,00	10,00	7,24	1,40
600,00	10,00		
600,00	10,00		
700,00	10,00		
800,00	10,00		
920,00	9,00		
480,00	10,00		

700,00	9,00	7,18	-3,20
580,00	10,00	7,22	-2,80
400,00	9,00		
750,00	9,00		
520,00	10,00	7,26	-4,20
545,00			
600,00	10,00		
650,00	10,00	7,31	1,80
540,00	10,00		
620,00	10,00		
760,00	6,00	7,10	-10,20
690,00	10,00		

700,00	9,00	7,32	-10,10
600,00	10,00	7,30	-5,10
590,00	10,00	7,27	-2,70
540,00	10,00	7,35	-1,10
550,00	10,00	7,26	-1,10
540,00	10,00		
400,00	10,00		
600,00	10,00		
580,00	10,00		
600,00	10,00	7,33	-,70
850,00	10,00		
500,00	10,00		

710,00	10,00	7,17	-1,90
680,00	10,00	7,21	-3,50
420,00	10,00		
450,00	8,00		
850,00	10,00	7,19	-7,30
575,00	10,00	7,32	-1,30
480,00	9,00	7,28	-1,10
600,00	10,00		
	9,00		
610,00	10,00	7,14	-6,60

500,00	10,00	7,33	-4,80
600,00	10,00	7,38	-1,20
	10,00		
700,00	10,00	7,18	-5,10
740,00	10,00		
770,00	10,00	7,15	-7,50
524,00	8,00	7,08	-12,10
640,00	9,00	7,18	-2,70
425,00	9,00	7,21	-4,40
720,00	10,00	7,18	-8,10
740,00	10,00	7,32	-3,50

420,00	9,00		
400,00	9,00	7,03	-9,30
660,00	10,00		
540,00	10,00		
540,00	9,00	7,14	-9,10
650,00	9,00		
600,00	2,00		
500,00	10,00	7,28	-3,40
680,00	9,00	7,20	-2,60
