# Supplementary material for: Maternal Functional Hemodynamics in the Second Half of Pregnancy: A Longitudinal Study
Source: PLoS One. 2015 Aug 10;10(8):e0135300. doi: 10.1371/journal.pone.0135300 (PMC4530890; doi:10.1371/journal.pone.0135300)
Supplement: S2 Table — (DOCX) [file pone.0135300.s002.docx]

**Table S 2.** **Longitudinal reference ranges** **for the maternal stroke volume (ml) during second half of pregnancy.**

| Gestation  (weeks) | 2.5th  percentile | 5th  percentile | 10th  percentile | 50th  percentile | 90th  percentile | 95th  percentile | 97.5th  percentile |
| --- | --- | --- | --- | --- | --- | --- | --- |
| 20 | 55 | 59 | 63 | 83 | 106 | 113 | 120 |
| 21 | 54 | 58 | 63 | 83 | 106 | 113 | 120 |
| 22 | 54 | 58 | 63 | 83 | 106 | 113 | 120 |
| 23 | 54 | 58 | 63 | 82 | 106 | 113 | 120 |
| 24 | 54 | 58 | 63 | 82 | 105 | 113 | 119 |
| 25 | 54 | 58 | 63 | 82 | 105 | 113 | 119 |
| 26 | 54 | 58 | 63 | 82 | 105 | 112 | 119 |
| 27 | 54 | 58 | 63 | 82 | 105 | 112 | 119 |
| 28 | 54 | 58 | 62 | 82 | 105 | 112 | 118 |
| 29 | 54 | 57 | 62 | 81 | 104 | 112 | 118 |
| 30 | 53 | 57 | 62 | 81 | 104 | 111 | 118 |
| 31 | 53 | 57 | 62 | 81 | 104 | 111 | 118 |
| 32 | 53 | 57 | 62 | 81 | 104 | 111 | 117 |
| 33 | 53 | 56 | 61 | 80 | 103 | 111 | 117 |
| 34 | 52 | 56 | 61 | 80 | 103 | 110 | 117 |
| 35 | 52 | 56 | 61 | 80 | 103 | 110 | 117 |
| 36 | 51 | 55 | 60 | 79 | 103 | 110 | 117 |
| 37 | 51 | 55 | 60 | 79 | 102 | 110 | 117 |
| 38 | 51 | 54 | 59 | 79 | 102 | 110 | 117 |
| 39 | 50 | 54 | 59 | 78 | 102 | 110 | 116 |
| 40 | 49 | 53 | 58 | 78 | 102 | 110 | 116 |
