# Supplementary material for: Maternal Functional Hemodynamics in the Second Half of Pregnancy: A Longitudinal Study
Source: PLoS One. 2015 Aug 10;10(8):e0135300. doi: 10.1371/journal.pone.0135300 (PMC4530890; doi:10.1371/journal.pone.0135300)
Supplement: S3 Table — (DOCX) [file pone.0135300.s003.docx]

**Table S 3.** **Longitudinal reference ranges** **for the maternal heart rate (beats/min) during second half of pregnancy.**

| Gestation  (weeks) | 2.5th  percentile | 5th  percentile | 10th  percentile | 50th  percentile | 90th  percentile | 95th  percentile | 97.5th  percentile |
| --- | --- | --- | --- | --- | --- | --- | --- |
| 20 | 60 | 63 | 66 | 79 | 96 | 102 | 107 |
| 21 | 61 | 64 | 67 | 80 | 97 | 103 | 108 |
| 22 | 62 | 64 | 67 | 81 | 98 | 104 | 109 |
| 23 | 62 | 65 | 68 | 81 | 99 | 105 | 110 |
| 24 | 62 | 65 | 68 | 82 | 100 | 106 | 111 |
| 25 | 63 | 65 | 69 | 83 | 101 | 107 | 112 |
| 26 | 63 | 66 | 69 | 83 | 102 | 108 | 114 |
| 27 | 63 | 66 | 69 | 84 | 103 | 109 | 115 |
| 28 | 63 | 66 | 70 | 84 | 104 | 110 | 117 |
| 29 | 64 | 67 | 70 | 85 | 105 | 112 | 118 |
| 30 | 64 | 67 | 70 | 86 | 106 | 113 | 119 |
| 31 | 64 | 67 | 71 | 86 | 107 | 114 | 121 |
| 32 | 64 | 67 | 71 | 87 | 108 | 116 | 123 |
| 33 | 64 | 67 | 71 | 87 | 109 | 117 | 124 |
| 34 | 64 | 67 | 71 | 88 | 110 | 118 | 126 |
| 35 | 64 | 67 | 71 | 88 | 111 | 120 | 127 |
| 36 | 64 | 67 | 71 | 89 | 113 | 121 | 129 |
| 37 | 64 | 67 | 72 | 89 | 114 | 122 | 131 |
| 38 | 64 | 67 | 72 | 90 | 115 | 124 | 132 |
| 39 | 64 | 67 | 72 | 90 | 116 | 125 | 134 |
| 40 | 64 | 67 | 72 | 91 | 117 | 127 | 136 |
