# Supplementary material for: Maternal Functional Hemodynamics in the Second Half of Pregnancy: A Longitudinal Study
Source: PLoS One. 2015 Aug 10;10(8):e0135300. doi: 10.1371/journal.pone.0135300 (PMC4530890; doi:10.1371/journal.pone.0135300)
Supplement: S4 Table — (DOCX) [file pone.0135300.s004.docx]

**Table S 4.** **Longitudinal reference ranges** **for the maternal cardiac output (L/min) during second half of pregnancy.**

| Gestation  (weeks) | 2.5th  percentile | 5th  percentile | 10th  percentile | 50th  percentile | 90th  percentile | 95th  percentile | 97.5th  percentile |
| --- | --- | --- | --- | --- | --- | --- | --- |
| 20 | 4.2 | 4.5 | 4.8 | 6.3 | 8.2 | 8.8 | 9.4 |
| 21 | 4.3 | 4.5 | 4.9 | 6.4 | 8.3 | 8.9 | 9.4 |
| 22 | 4.3 | 4.6 | 5.0 | 6.5 | 8.3 | 9.0 | 9.5 |
| 23 | 4.4 | 4.7 | 5.0 | 6.5 | 8.4 | 9.0 | 9.6 |
| 24 | 4.4 | 4.7 | 5.1 | 6.6 | 8.5 | 9.1 | 9.7 |
| 25 | 4.4 | 4.7 | 5.1 | 6.6 | 8.6 | 9.2 | 9.8 |
| 26 | 4.5 | 4.8 | 5.1 | 6.7 | 8.6 | 9.3 | 9.8 |
| 27 | 4.5 | 4.8 | 5.2 | 6.7 | 8.7 | 9.3 | 9.9 |
| 28 | 4.5 | 4.8 | 5.2 | 6.8 | 8.7 | 9.4 | 10.0 |
| 29 | 4.5 | 4.8 | 5.2 | 6.8 | 8.8 | 9.5 | 10.1 |
| 30 | 4.5 | 4.8 | 5.2 | 6.8 | 8.8 | 9.5 | 10.1 |
| 31 | 4.5 | 4.8 | 5.2 | 6.8 | 8.9 | 9.6 | 10.2 |
| 32 | 4.5 | 4.8 | 5.2 | 6.9 | 8.9 | 9.6 | 10.3 |
| 33 | 4.5 | 4.8 | 5.2 | 6.9 | 9.0 | 9.7 | 10.3 |
| 34 | 4.5 | 4.8 | 5.2 | 6.9 | 9.0 | 9.7 | 10.4 |
| 35 | 4.5 | 4.8 | 5.3 | 6.9 | 9.1 | 9.8 | 10.4 |
| 36 | 4.5 | 4.9 | 5.3 | 6.9 | 9.1 | 9.8 | 10.5 |
| 37 | 4.5 | 4.9 | 5.3 | 7.0 | 9.1 | 9.8 | 10.5 |
| 38 | 4.5 | 4.9 | 5.3 | 7.0 | 9.2 | 9.9 | 10.6 |
| 39 | 4.5 | 4.9 | 5.3 | 7.0 | 9.2 | 9.9 | 10.6 |
| 40 | 4.5 | 4.9 | 5.3 | 7.0 | 9.2 | 9.9 | 10.6 |
