# Supplementary material for: Maternal Functional Hemodynamics in the Second Half of Pregnancy: A Longitudinal Study
Source: PLoS One. 2015 Aug 10;10(8):e0135300. doi: 10.1371/journal.pone.0135300 (PMC4530890; doi:10.1371/journal.pone.0135300)
Supplement: S5 Table — (DOCX) [file pone.0135300.s005.docx]

**Table S 5.** **Longitudinal reference ranges** **for the maternal mean arterial pressure (mmHg) during second half of pregnancy.**

| Gestation  (weeks) | 2.5th  percentile | 5th  percentile | 10th  percentile | 50th  percentile | 90th  percentile | 95th  percentile | 97.5th  percentile |
| --- | --- | --- | --- | --- | --- | --- | --- |
| 20 | 70 | 72 | 74 | 82 | 93 | 96 | 99 |
| 21 | 69 | 70 | 73 | 81 | 91 | 94 | 97 |
| 22 | 68 | 70 | 72 | 80 | 89 | 93 | 95 |
| 23 | 67 | 69 | 71 | 79 | 89 | 92 | 95 |
| 24 | 67 | 69 | 71 | 79 | 88 | 91 | 94 |
| 25 | 67 | 69 | 71 | 79 | 88 | 91 | 94 |
| 26 | 67 | 69 | 71 | 79 | 89 | 92 | 94 |
| 27 | 68 | 69 | 71 | 79 | 89 | 92 | 95 |
| 28 | 68 | 70 | 72 | 80 | 89 | 92 | 95 |
| 29 | 68 | 70 | 72 | 80 | 90 | 93 | 96 |
| 30 | 69 | 70 | 73 | 81 | 91 | 94 | 97 |
| 31 | 69 | 71 | 73 | 81 | 91 | 95 | 97 |
| 32 | 70 | 71 | 74 | 82 | 92 | 95 | 98 |
| 33 | 70 | 72 | 74 | 83 | 93 | 96 | 99 |
| 34 | 71 | 72 | 75 | 83 | 94 | 97 | 100 |
| 35 | 71 | 73 | 75 | 84 | 94 | 98 | 101 |
| 36 | 72 | 74 | 76 | 85 | 95 | 99 | 102 |
| 37 | 72 | 74 | 76 | 85 | 96 | 100 | 103 |
| 38 | 73 | 75 | 77 | 86 | 97 | 100 | 104 |
| 39 | 73 | 75 | 77 | 87 | 98 | 101 | 105 |
| 40 | 74 | 76 | 78 | 87 | 99 | 102 | 105 |
