# Supplementary material for: Maternal Functional Hemodynamics in the Second Half of Pregnancy: A Longitudinal Study
Source: PLoS One. 2015 Aug 10;10(8):e0135300. doi: 10.1371/journal.pone.0135300 (PMC4530890; doi:10.1371/journal.pone.0135300)
Supplement: S6 Table — (DOCX) [file pone.0135300.s006.docx]

**Table S 6.** **Longitudinal reference ranges** **for the maternal systemic vascular resistance (dyne s/cm^5^) during second half of pregnancy.**

| Gestation  (weeks) | 2.5th  percentile | 5th  percentile | 10th  percentile | 50th  percentile | 90th  percentile | 95th  percentile | 97.5th  percentile |
| --- | --- | --- | --- | --- | --- | --- | --- |
| 20 | 634 | 671 | 717 | 918 | 1199 | 1298 | 1393 |
| 21 | 634 | 671 | 717 | 918 | 1199 | 1298 | 1393 |
| 22 | 633 | 671 | 717 | 918 | 1198 | 1298 | 1393 |
| 23 | 633 | 671 | 717 | 918 | 1198 | 1298 | 1393 |
| 24 | 633 | 671 | 717 | 918 | 1198 | 1298 | 1393 |
| 25 | 633 | 670 | 717 | 918 | 1198 | 1298 | 1393 |
| 26 | 633 | 670 | 717 | 918 | 1198 | 1298 | 1393 |
| 27 | 633 | 670 | 717 | 918 | 1198 | 1298 | 1393 |
| 28 | 633 | 670 | 717 | 918 | 1198 | 1298 | 1393 |
| 29 | 633 | 670 | 717 | 918 | 1198 | 1298 | 1393 |
| 30 | 633 | 670 | 717 | 918 | 1198 | 1298 | 1393 |
| 31 | 633 | 670 | 717 | 918 | 1198 | 1298 | 1393 |
| 32 | 633 | 670 | 717 | 918 | 1198 | 1297 | 1393 |
| 33 | 633 | 670 | 717 | 918 | 1198 | 1297 | 1392 |
| 34 | 633 | 670 | 717 | 918 | 1198 | 1297 | 1392 |
| 35 | 633 | 670 | 717 | 917 | 1198 | 1297 | 1392 |
| 36 | 633 | 670 | 717 | 917 | 1198 | 1297 | 1392 |
| 37 | 633 | 670 | 717 | 917 | 1198 | 1297 | 1392 |
| 38 | 633 | 670 | 717 | 917 | 1198 | 1297 | 1392 |
| 39 | 633 | 670 | 717 | 917 | 1198 | 1297 | 1392 |
| 40 | 633 | 670 | 717 | 917 | 1198 | 1297 | 1392 |
