# Supplementary material for: Maternal Functional Hemodynamics in the Second Half of Pregnancy: A Longitudinal Study
Source: PLoS One. 2015 Aug 10;10(8):e0135300. doi: 10.1371/journal.pone.0135300 (PMC4530890; doi:10.1371/journal.pone.0135300)
Supplement: S7 Table — (DOCX) [file pone.0135300.s007.docx]

**Table S 7.** **Longitudinal reference ranges** **for the maternal thoracic fluid content (1/kOhm) during second half of pregnancy.**

| Gestation  (weeks) | 2.5th  percentile | 5th  percentile | 10th  percentile | 50th  percentile | 90th  percentile | 95th  percentile | 97.5th  percentile |
| --- | --- | --- | --- | --- | --- | --- | --- |
| 20 | 21.0 | 22.1 | 23.5 | 28.3 | 33.2 | 34.6 | 35.8 |
| 21 | 21.1 | 22.2 | 23.6 | 28.4 | 33.3 | 34.7 | 35.9 |
| 22 | 21.2 | 22.3 | 23.7 | 28.5 | 33.4 | 34.8 | 36.0 |
| 23 | 21.2 | 22.4 | 23.8 | 28.6 | 33.5 | 34.9 | 36.1 |
| 24 | 21.3 | 22.5 | 23.9 | 28.7 | 33.6 | 35.0 | 36.2 |
| 25 | 21.4 | 22.6 | 23.9 | 28.8 | 33.7 | 35.1 | 36.3 |
| 26 | 21.5 | 22.7 | 24.0 | 28.9 | 33.8 | 35.2 | 36.4 |
| 27 | 21.6 | 22.8 | 24.1 | 29.0 | 33.9 | 35.3 | 36.5 |
| 28 | 21.7 | 22.9 | 24.2 | 29.1 | 34.0 | 35.4 | 36.6 |
| 29 | 21.8 | 23.0 | 24.3 | 29.2 | 34.1 | 35.5 | 36.7 |
| 30 | 21.9 | 23.1 | 24.4 | 29.3 | 34.2 | 35.6 | 36.8 |
| 31 | 22.0 | 23.2 | 24.5 | 29.4 | 34.3 | 35.7 | 36.9 |
| 32 | 22.1 | 23.3 | 24.6 | 29.5 | 34.4 | 35.8 | 37.0 |
| 33 | 22.2 | 23.4 | 24.7 | 29.5 | 34.5 | 35.9 | 37.1 |
| 34 | 22.3 | 23.5 | 24.8 | 29.6 | 34.6 | 36.0 | 37.2 |
| 35 | 22.4 | 23.6 | 24.9 | 29.7 | 34.7 | 36.1 | 37.3 |
| 36 | 22.5 | 23.7 | 25.0 | 29.8 | 34.8 | 36.2 | 37.4 |
| 37 | 22.6 | 23.8 | 25.1 | 29.9 | 34.9 | 36.3 | 37.5 |
| 38 | 22.7 | 23.8 | 25.2 | 30.0 | 35.0 | 36.4 | 37.6 |
| 39 | 22.8 | 23.9 | 25.3 | 30.1 | 35.1 | 36.5 | 37.7 |
| 40 | 22.9 | 24.0 | 25.4 | 30.2 | 35.2 | 36.6 | 37.8 |
