# Supplementary material for: Maternal Functional Hemodynamics in the Second Half of Pregnancy: A Longitudinal Study
Source: PLoS One. 2015 Aug 10;10(8):e0135300. doi: 10.1371/journal.pone.0135300 (PMC4530890; doi:10.1371/journal.pone.0135300)
Supplement: S8 Table — (DOCX) [file pone.0135300.s008.docx]

**Table S 8.** **Longitudinal reference ranges** **for the maternal acceleration index (1 /100 s^2^) during second half of pregnancy.**

| Gestation  (weeks) | 2.5th  percentile | 5th  percentile | 10th  percentile | 50th  percentile | 90th  percentile | 95th  percentile | 97.5th  percentile |
| --- | --- | --- | --- | --- | --- | --- | --- |
| 20 | 79 | 89 | 101 | 153 | 222 | 245 | 266 |
| 21 | 78 | 87 | 99 | 151 | 218 | 241 | 262 |
| 22 | 76 | 85 | 97 | 148 | 214 | 237 | 257 |
| 23 | 74 | 83 | 95 | 145 | 211 | 233 | 253 |
| 24 | 72 | 81 | 93 | 142 | 207 | 229 | 249 |
| 25 | 71 | 79 | 91 | 139 | 203 | 225 | 245 |
| 26 | 69 | 78 | 89 | 136 | 200 | 221 | 241 |
| 27 | 67 | 76 | 87 | 134 | 196 | 217 | 237 |
| 28 | 66 | 74 | 85 | 131 | 193 | 214 | 233 |
| 29 | 64 | 72 | 83 | 128 | 189 | 210 | 229 |
| 30 | 62 | 71 | 81 | 126 | 186 | 206 | 225 |
| 31 | 61 | 69 | 79 | 123 | 183 | 203 | 221 |
| 32 | 59 | 67 | 77 | 121 | 179 | 199 | 217 |
| 33 | 58 | 66 | 75 | 118 | 176 | 196 | 214 |
| 34 | 56 | 64 | 74 | 116 | 173 | 192 | 210 |
| 35 | 55 | 62 | 72 | 114 | 170 | 189 | 206 |
| 36 | 53 | 61 | 70 | 111 | 167 | 185 | 203 |
| 37 | 52 | 59 | 68 | 109 | 163 | 182 | 199 |
| 38 | 51 | 58 | 67 | 107 | 160 | 179 | 196 |
| 39 | 49 | 56 | 65 | 104 | 157 | 175 | 192 |
| 40 | 48 | 55 | 64 | 102 | 154 | 172 | 189 |
