# Supplementary material for: Maternal Functional Hemodynamics in the Second Half of Pregnancy: A Longitudinal Study
Source: PLoS One. 2015 Aug 10;10(8):e0135300. doi: 10.1371/journal.pone.0135300 (PMC4530890; doi:10.1371/journal.pone.0135300)
Supplement: S9 Table — (DOCX) [file pone.0135300.s009.docx]

**Table S 9.** **Longitudinal reference ranges** **for the maternal velocity index (1/1000s) during second half of pregnancy.**

| Gestation  (weeks) | 2.5th  percentile | 5th  percentile | 10th  percentile | 50th  percentile | 90th  percentile | 95th  percentile | 97.5th  percentile |
| --- | --- | --- | --- | --- | --- | --- | --- |
| 20 | 56 | 61 | 67 | 93 | 126 | 136 | 146 |
| 21 | 55 | 60 | 67 | 92 | 124 | 135 | 144 |
| 22 | 54 | 59 | 66 | 91 | 123 | 133 | 143 |
| 23 | 54 | 59 | 65 | 90 | 122 | 132 | 141 |
| 24 | 53 | 58 | 64 | 89 | 120 | 130 | 140 |
| 25 | 52 | 57 | 63 | 88 | 119 | 129 | 138 |
| 26 | 51 | 56 | 62 | 87 | 117 | 127 | 136 |
| 27 | 50 | 55 | 61 | 85 | 116 | 125 | 134 |
| 28 | 49 | 54 | 60 | 84 | 114 | 124 | 133 |
| 29 | 48 | 53 | 59 | 83 | 112 | 122 | 131 |
| 30 | 47 | 52 | 58 | 81 | 111 | 120 | 129 |
| 31 | 47 | 51 | 57 | 80 | 109 | 118 | 127 |
| 32 | 46 | 50 | 56 | 78 | 107 | 116 | 125 |
| 33 | 45 | 49 | 54 | 77 | 105 | 115 | 123 |
| 34 | 44 | 48 | 53 | 75 | 103 | 113 | 121 |
| 35 | 43 | 47 | 52 | 74 | 102 | 111 | 119 |
| 36 | 41 | 46 | 51 | 72 | 100 | 109 | 117 |
| 37 | 40 | 45 | 50 | 71 | 98 | 106 | 114 |
| 38 | 39 | 43 | 48 | 69 | 96 | 104 | 112 |
| 39 | 38 | 42 | 47 | 68 | 94 | 102 | 110 |
| 40 | 37 | 41 | 46 | 66 | 92 | 100 | 108 |
