# Supplementary material for: Maternal Functional Hemodynamics in the Second Half of Pregnancy: A Longitudinal Study
Source: PLoS One. 2015 Aug 10;10(8):e0135300. doi: 10.1371/journal.pone.0135300 (PMC4530890; doi:10.1371/journal.pone.0135300)
Supplement: S10 Table — (DOCX) [file pone.0135300.s010.docx]

**Table S 10.** **Longitudinal reference ranges** **for the maternal pre-ejection period (ms) during second half of pregnancy.**

| Gestation  (weeks) | 2.5th  percentile | 5th  percentile | 10th  percentile | 50th  percentile | 90th  percentile | 95th  percentile | 97.5th  percentile |
| --- | --- | --- | --- | --- | --- | --- | --- |
| 20 | 40 | 46 | 52 | 74 | 97 | 104 | 110 |
| 21 | 41 | 46 | 52 | 75 | 98 | 104 | 110 |
| 22 | 41 | 47 | 53 | 75 | 98 | 105 | 111 |
| 23 | 42 | 47 | 54 | 76 | 99 | 106 | 112 |
| 24 | 43 | 48 | 54 | 77 | 100 | 106 | 112 |
| 25 | 43 | 49 | 55 | 77 | 101 | 107 | 113 |
| 26 | 44 | 50 | 56 | 78 | 101 | 108 | 114 |
| 27 | 45 | 50 | 57 | 79 | 102 | 109 | 115 |
| 28 | 46 | 51 | 58 | 80 | 103 | 110 | 116 |
| 29 | 47 | 52 | 58 | 81 | 104 | 111 | 117 |
| 30 | 48 | 53 | 60 | 82 | 105 | 112 | 118 |
| 31 | 49 | 54 | 61 | 83 | 107 | 113 | 119 |
| 32 | 50 | 56 | 62 | 85 | 108 | 115 | 121 |
| 33 | 51 | 57 | 63 | 86 | 109 | 116 | 122 |
| 34 | 53 | 58 | 65 | 87 | 111 | 117 | 123 |
| 35 | 54 | 60 | 66 | 89 | 112 | 119 | 125 |
| 36 | 56 | 61 | 68 | 90 | 114 | 121 | 127 |
| 37 | 57 | 63 | 69 | 92 | 116 | 122 | 128 |
| 38 | 59 | 65 | 71 | 94 | 118 | 124 | 130 |
| 39 | 61 | 66 | 73 | 96 | 119 | 126 | 132 |
| 40 | 63 | 68 | 75 | 98 | 121 | 128 | 134 |
