# Supplementary material for: Maternal Functional Hemodynamics in the Second Half of Pregnancy: A Longitudinal Study
Source: PLoS One. 2015 Aug 10;10(8):e0135300. doi: 10.1371/journal.pone.0135300 (PMC4530890; doi:10.1371/journal.pone.0135300)
Supplement: S11 Table — (DOCX) [file pone.0135300.s011.docx]

**Table S 11.** **Longitudinal reference ranges** **for the maternal left ventricular ejection time (ms) during second half of pregnancy.**

| Gestation  (weeks) | 2.5th  percentile | 5th  percentile | 10th  percentile | 50th  percentile | 90th  percentile | 95th  percentile | 97.5th  percentile |
| --- | --- | --- | --- | --- | --- | --- | --- |
| 20 | 198 | 210 | 223 | 265 | 302 | 312 | 321 |
| 21 | 196 | 208 | 221 | 263 | 301 | 311 | 319 |
| 22 | 194 | 206 | 219 | 261 | 299 | 309 | 318 |
| 23 | 193 | 204 | 217 | 260 | 298 | 308 | 317 |
| 24 | 191 | 203 | 216 | 258 | 297 | 307 | 315 |
| 25 | 189 | 201 | 215 | 257 | 295 | 306 | 314 |
| 26 | 188 | 200 | 213 | 256 | 294 | 305 | 313 |
| 27 | 187 | 199 | 212 | 255 | 293 | 304 | 312 |
| 28 | 185 | 197 | 211 | 254 | 293 | 303 | 312 |
| 29 | 184 | 196 | 210 | 253 | 292 | 302 | 311 |
| 30 | 183 | 195 | 209 | 252 | 291 | 301 | 310 |
| 31 | 182 | 194 | 208 | 251 | 290 | 301 | 309 |
| 32 | 181 | 193 | 207 | 251 | 290 | 300 | 309 |
| 33 | 180 | 193 | 206 | 250 | 289 | 299 | 308 |
| 34 | 179 | 192 | 205 | 249 | 288 | 299 | 307 |
| 35 | 179 | 191 | 205 | 249 | 288 | 298 | 307 |
| 36 | 178 | 190 | 204 | 248 | 287 | 298 | 306 |
| 37 | 177 | 190 | 203 | 247 | 287 | 297 | 306 |
| 38 | 176 | 189 | 203 | 247 | 286 | 297 | 305 |
| 39 | 176 | 188 | 202 | 246 | 286 | 296 | 305 |
| 40 | 175 | 188 | 202 | 246 | 285 | 296 | 305 |
