# Supplementary material for: Maternal Functional Hemodynamics in the Second Half of Pregnancy: A Longitudinal Study
Source: PLoS One. 2015 Aug 10;10(8):e0135300. doi: 10.1371/journal.pone.0135300 (PMC4530890; doi:10.1371/journal.pone.0135300)
Supplement: S12 Table — (DOCX) [file pone.0135300.s012.docx]

**Table S 12.** **Longitudinal reference ranges** **for the maternal systolic time ratio (%) during second half of pregnancy.**

| Gestation  (weeks) | 2.5th  percentile | 5th  percentile | 10th  percentile | 50th  percentile | 90th  percentile | 95th  percentile | 97.5th  percentile |
| --- | --- | --- | --- | --- | --- | --- | --- |
| 20 | 16.9 | 18.6 | 20.6 | 29.0 | 39.5 | 43.0 | 46.1 |
| 21 | 17.1 | 18.7 | 20.8 | 29.2 | 39.9 | 43.3 | 46.5 |
| 22 | 17.2 | 18.9 | 21.0 | 29.5 | 40.2 | 43.7 | 46.9 |
| 23 | 17.4 | 19.1 | 21.2 | 29.8 | 40.7 | 44.2 | 47.4 |
| 24 | 17.6 | 19.3 | 21.4 | 30.2 | 41.1 | 44.7 | 47.9 |
| 25 | 17.8 | 19.5 | 21.7 | 30.5 | 41.6 | 45.2 | 48.5 |
| 26 | 18.0 | 19.8 | 21.9 | 30.9 | 42.2 | 45.8 | 49.2 |
| 27 | 18.3 | 20.0 | 22.2 | 31.3 | 42.8 | 46.5 | 49.9 |
| 28 | 18.5 | 20.3 | 22.5 | 31.8 | 43.5 | 47.2 | 50.7 |
| 29 | 18.8 | 20.6 | 22.9 | 32.3 | 44.2 | 48.1 | 51.6 |
| 30 | 19.0 | 20.9 | 23.2 | 32.9 | 45.0 | 49.0 | 52.6 |
| 31 | 19.3 | 21.2 | 23.6 | 33.5 | 45.9 | 50.0 | 53.7 |
| 32 | 19.6 | 21.6 | 24.0 | 34.1 | 46.9 | 51.1 | 54.9 |
| 33 | 19.9 | 21.9 | 24.4 | 34.8 | 48.0 | 52.3 | 56.2 |
| 34 | 20.2 | 22.3 | 24.8 | 35.6 | 49.2 | 53.6 | 57.7 |
| 35 | 20.5 | 22.6 | 25.3 | 36.4 | 50.5 | 55.1 | 59.3 |
| 36 | 20.8 | 23.0 | 25.8 | 37.3 | 51.9 | 56.7 | 61.1 |
| 37 | 21.1 | 23.4 | 26.3 | 38.2 | 53.5 | 58.5 | 63.1 |
| 38 | 21.5 | 23.8 | 26.8 | 39.2 | 55.2 | 60.4 | 65.3 |
| 39 | 21.8 | 24.2 | 27.3 | 40.3 | 57.1 | 62.6 | 67.7 |
| 40 | 22.1 | 24.7 | 27.9 | 41.4 | 59.1 | 64.9 | 70.3 |
