# Supplementary material for: Maternal Functional Hemodynamics in the Second Half of Pregnancy: A Longitudinal Study
Source: PLoS One. 2015 Aug 10;10(8):e0135300. doi: 10.1371/journal.pone.0135300 (PMC4530890; doi:10.1371/journal.pone.0135300)
Supplement: S13 Table — (DOCX) [file pone.0135300.s013.docx]

**Table S 13.** **Longitudinal reference ranges** **for the maternal left ventricular work index (Kg m/m^2^) during second half of pregnancy.**

| Gestation  (weeks) | 2.5th  percentile | 5th  percentile | 10th  percentile | 50th  percentile | 90th  percentile | 95th  percentile | 97.5th  percentile |
| --- | --- | --- | --- | --- | --- | --- | --- |
| 20 | 2.4 | 2.6 | 2.8 | 3.7 | 4.9 | 5.3 | 5.7 |
| 21 | 2.4 | 2.6 | 2.8 | 3.7 | 4.9 | 5.3 | 5.7 |
| 22 | 2.4 | 2.6 | 2.8 | 3.7 | 4.9 | 5.3 | 5.7 |
| 23 | 2.5 | 2.6 | 2.8 | 3.7 | 4.9 | 5.3 | 5.7 |
| 24 | 2.5 | 2.7 | 2.9 | 3.8 | 4.9 | 5.4 | 5.7 |
| 25 | 2.5 | 2.7 | 2.9 | 3.8 | 5.0 | 5.4 | 5.8 |
| 26 | 2.5 | 2.7 | 2.9 | 3.8 | 5.0 | 5.4 | 5.8 |
| 27 | 2.6 | 2.7 | 2.9 | 3.8 | 5.0 | 5.4 | 5.8 |
| 28 | 2.6 | 2.7 | 3.0 | 3.9 | 5.1 | 5.5 | 5.9 |
| 29 | 2.6 | 2.8 | 3.0 | 3.9 | 5.1 | 5.5 | 5.9 |
| 30 | 2.6 | 2.8 | 3.0 | 3.9 | 5.1 | 5.6 | 5.9 |
| 31 | 2.6 | 2.8 | 3.0 | 3.9 | 5.2 | 5.6 | 6.0 |
| 32 | 2.6 | 2.8 | 3.0 | 4.0 | 5.2 | 5.6 | 6.0 |
| 33 | 2.7 | 2.8 | 3.1 | 4.0 | 5.2 | 5.7 | 6.1 |
| 34 | 2.7 | 2.9 | 3.1 | 4.0 | 5.3 | 5.7 | 6.1 |
| 35 | 2.7 | 2.9 | 3.1 | 4.0 | 5.3 | 5.8 | 6.2 |
| 36 | 2.7 | 2.9 | 3.1 | 4.1 | 5.4 | 5.8 | 6.3 |
| 37 | 2.7 | 2.9 | 3.1 | 4.1 | 5.4 | 5.9 | 6.3 |
| 38 | 2.7 | 2.9 | 3.1 | 4.1 | 5.5 | 5.9 | 6.4 |
| 39 | 2.7 | 2.9 | 3.2 | 4.2 | 5.5 | 6.0 | 6.4 |
| 40 | 2.7 | 2.9 | 3.2 | 4.2 | 5.6 | 6.1 | 6.5 |
