# Supplementary material for: Maternal Functional Hemodynamics in the Second Half of Pregnancy: A Longitudinal Study
Source: PLoS One. 2015 Aug 10;10(8):e0135300. doi: 10.1371/journal.pone.0135300 (PMC4530890; doi:10.1371/journal.pone.0135300)
Supplement: S14 Table — Data are presented as mean values for baseline and PLR (± standard deviation). GA, gestational age; SV, stroke volume (ml); HR, heart rate (beats/min); CO, cardiac output (L/min); MAP, mean arterial pressure (mmHg); SVR, systemic vascular resistance (dyne s/cm5); TFC, thoracic fluid content (1/kOhm); ACI, acceleration index (1/100 s2); VI, velocity index (1/1000s); PEP, pre-ejection period (ms); LVET, left ventricular ejection time (ms); STR, systolic time ratio (%) and LCWI, left ventricular work index (Kg m/m2). (DOCX) [file pone.0135300.s014.docx]

**Table S 14. Hemodynamic variables measured by impedance cardiography at baseline and 90 seconds after passive leg raising during the second half of pregnancy.**

| **Variables** | **GA (weeks)** | **p-value** | **GA (weeks)** | **p-value** | **GA (weeks)** | **p-value** | **GA (weeks)** | **p-value** | **GA (weeks)** | **p-value** |
| --- | --- | --- | --- | --- | --- | --- | --- | --- | --- | --- |
|  | **20-24**  **Baseline - PLR** |  | **24-28**  **Baseline - PLR** |  | **28-32**  **Baseline - PLR** |  | **32-36**  **Baseline - PLR** |  | **˃ 36**  **Baseline - PLR** |  |
| SV | 82.97 (±15.83) -  84.11 (16.40) | 0.121 | 83.64 (±17.03) -  84.37 (±16.97) | 0.390 | 84.56 (±16.74) -  83.54 (±16.24) | 0.272 | 80.03 (±16.24) –  78.23 (±15.76) | 0.139 | 81.68 (±16.62) –  80.68 (±15.02) | 0.418 |
| HR | 82.13 (±12.77) -  79.59 (±11.28) | <0.001 | 83.75 (±12.47) -  81.23 (±11.41) | <0.001 | 87.17 (±12.24) -  83.54 (±11.41) | <0.001 | 90.60 (±16.97) – 87.79 (±15.32) | 0.008 | 90.37 (±16.01) –  85.32 (13.93) | <0.001 |
| CO | 6.58 (±1.34) -  6.54(±1.31) | 0.496 | 6.78 (±1.40) -  6.62 (±1.32) | 0.017 | 7.14 (±1.46) -  6.83 (±1.28) | <0.001 | 7.03 (±1.57 ) –  6.63 (±1.43) | <0.001 | 7.11 (±1.46) –  6.73 (±1.34) | <0.001 |
| MAP | 80.10 (±7.07) -  75.52 (±6.30) | <0.001 | 79.65 (±6.90 -­  76.45 ± 6.11) | <0.001 | 81.88 (±7.33) -  78.46 (±6.78) | <0.001 | 83.41 (±7.17) –  79.64 (±7.27) | <0.001 | 86.56 (±7.92) –  83.29 (±8.33) | <0.001 |
| SVR | 956.73 (±178.75) -  906.22 (±163.82) | <0.001 | 925.00 (±185.19) -  901.28 (±161.56) | 0.036 | 898.78 (±166.13) -895.33 (±149.52) | <0.727 | 944.13 (±209.74) –  948.63 (±196.45) | 0.714 | 971.22 (±231.25) -976.59 (±210.91) | 0.664 |
| TFC | 28.63 (±3.44) -  29.67 (±3.59) | <0.001 | 28.41 (±3.75) -  30.00 (±6.39) | 0.003 | 29.23 (±4.06) -  30.01 (±3.75) | <0.001 | 29.85 (±4.09) –  30.85 (±3.90) | <0.001 | 30.16 (±3.83) –  30.94 (±4.16) | <0.001 |
| ACI | 149.02 (±42.79) -  142.94 (±40.98) | 0.018 | 138.49 (±48.69) -  132.89 (±47.31) | 0.205 | 133.90 (±41.62) -  124.11 (±35.21) | <0.001 | 124.13 (±37.24) –  110.74(±33.91) | <0.001 | 111.56 (±40.10) -100.85 (±32.41) | 0.001 |
| VI | 90.82 (±20.18) ­-  84.15 (±18.06) | <0.001 | 87.54 (±22.55) -  80.84 (±21.41) | 0.001 | 85.81 (±22.30) -  80.21 (±19.72) | <0.001 | 79.20 (±19.66) –  71.05 (±18.17) | <0.001 | 71.45 (±19.17) –  65.83 (±16.53) | <0.001 |
| PEP | 76.17 (±15.10) -  68.37(±12.02) | <0.001 | 80.25 (±17.11) -  75.60 (±17.03) | <0.001 | 81.18 (±17.54) -  80.83 (±17.69) | 0.813 | 85.83 (±21.60) –  87.58 ±20.98) | 0.340 | 94.66 (±19.46) –  91.41 (±20.59) | 0.127 |
| LVET | 260.13 (±31.41) -  270.93 (±34.75) | 0.005 | 254.41 (±32.09) - 268.16 (±32.81) | <0.001 | 252.98 (±30.14) -  258.37 (±31.53) | 0.140 | 246.48 (±32.96) –  253.15 (±34.77) | 0.080 | 247.44 (±33.63) –  252.78 (±33.89) | 0.151 |
| STR | 30.07 (±6.66) -  26.01(±5.71) | <0.001 | 32.43 (±7.89) -  29.08 (±8.12) | <0.001 | 33.08 (±8.65) -  32.44 (±9.38) | 0.488 | 36.05 (±10.56) –  35.98 (±10.93) | 0.947 | 39.94 (±10.85) –  37.52 (±10.49) | 0.053 |
| LCWI | 3.81 (±0.85) -  3.53 (±0.72) | <0.001 | 3.83 (±0.81) -  3.57 (±0.72) | <0.001 | 4.13 (±0.89) -  3.76 (±0.74) | <0.001 | 4.14 (±0.97) –  3.71 (±0.84) | <0.001 | 4.21 (±0.81) –  3.84 (±0.81) | <0.001 |

Data are presented as mean values for baseline and PLR (± standard deviation). GA, gestational age; SV, stroke volume (ml); HR, heart rate (beats/min); CO, cardiac output (L/min); MAP, mean arterial pressure (mm Hg); SVR, systemic vascular resistance (dyne s/cm^5^); TFC, thoracic fluid content (1/kOhm); ACI, acceleration index (1/100 s^2^); VI, velocity index (1/1000s); PEP, pre-ejection period (ms); LVET, left ventricular ejection time (ms); STR, systolic time ratio (%) and LCWI, left ventricular work index (Kg m/m^2^).
